# Recent advances on natural depsidones: sources, biosynthesis, structure-activity relationship, and bioactivities

**DOI:** 10.7717/peerj.15394

**Published:** 2023-05-12

**Authors:** Maan T. Khayat, Kholoud F. Ghazawi, Waad A. Samman, Aisha A. Alhaddad, Gamal A. Mohamed, Sabrin RM Ibrahim

**Affiliations:** 1 Department of Pharmaceutical Chemistry, Faculty of Pharmacy, King Abdulaziz University, Jeddah, Saudi Arabia; 2 Clinical Pharmacy Department, College of Pharmacy, Umm Al-Qura University, Makkah, Saudi Arabia; 3 Department of Pharmacology and Toxicology, College of Pharmacy, Taibah University, Al-Madinah Al-Munawwarah, Saudi Arabia; 4 Department of Natural Products and Alternative Medicine, Faculty of Pharmacy, King Abdulaziz University, Jeddah, Saudi Arabia; 5 Department of Pharmacognosy, Faculty of Pharmacy, Assiut University, Assiut, Egypt; 6 Department of Chemistry, Batterjee Medical College, Jeddah, Saudi Arabia

**Keywords:** Depsidones, Lichens, Fungi, Biosynthesis, Life on land, Bioactivities, Polyketides, Drug discovery

## Abstract

Depsidones are a class of polyphenolic polyketides that have been proposed to be biosynthesized from oxidative coupling of esters of two polyketidic benzoic acid derivatives. They are principally encountered in fungi and lichens. In addition to their diversified structural features, they revealed varied bioactivities such as antimicrobial, antimalarial, cytotoxic, anti-inflammatory, anti-*Helicobacter pylori*, antimycobacterial, antihypertensive, anti-diarrheal, antidiabetic, phytotoxic, anti-HIV, anti-osteoclastogenic, and butyrylcholinesterase, tyrosinase, hyaluronidase, and acetylcholinesterase inhibition. The current work was targeted to provide an overview on the naturally reported depsidones from various sources in the period from 2018 to the end of 2022 including their structures, biosynthesis, sources, and bioactivities, as well as the reported structure-activity relationship and semisynthetic derivatives. A total of 172 metabolites with 87 references were reviewed. The reported findings unambiguously demonstrated that these derivatives could be promising leads for therapeutic agents. However, further *in-vivo* evaluation of their potential biological properties and mechanistic investigations are needed.

## Introduction

Nature affords unlimited riches of novel biomolecules that are derived from living organisms, including animals, plants, and microorganisms (Abdel-Razek et al., 2020). These metabolites have played a fundamental role for thousands of years as remediation for various human illnesses because of their availability and low cost, particularly in developing countries. Also, their chemical diversity with broad bioactivities makes them invaluable sources of drug development and discovery (Abdel-Razek et al., 2020; Shen, 2015).

**Figure 1:**
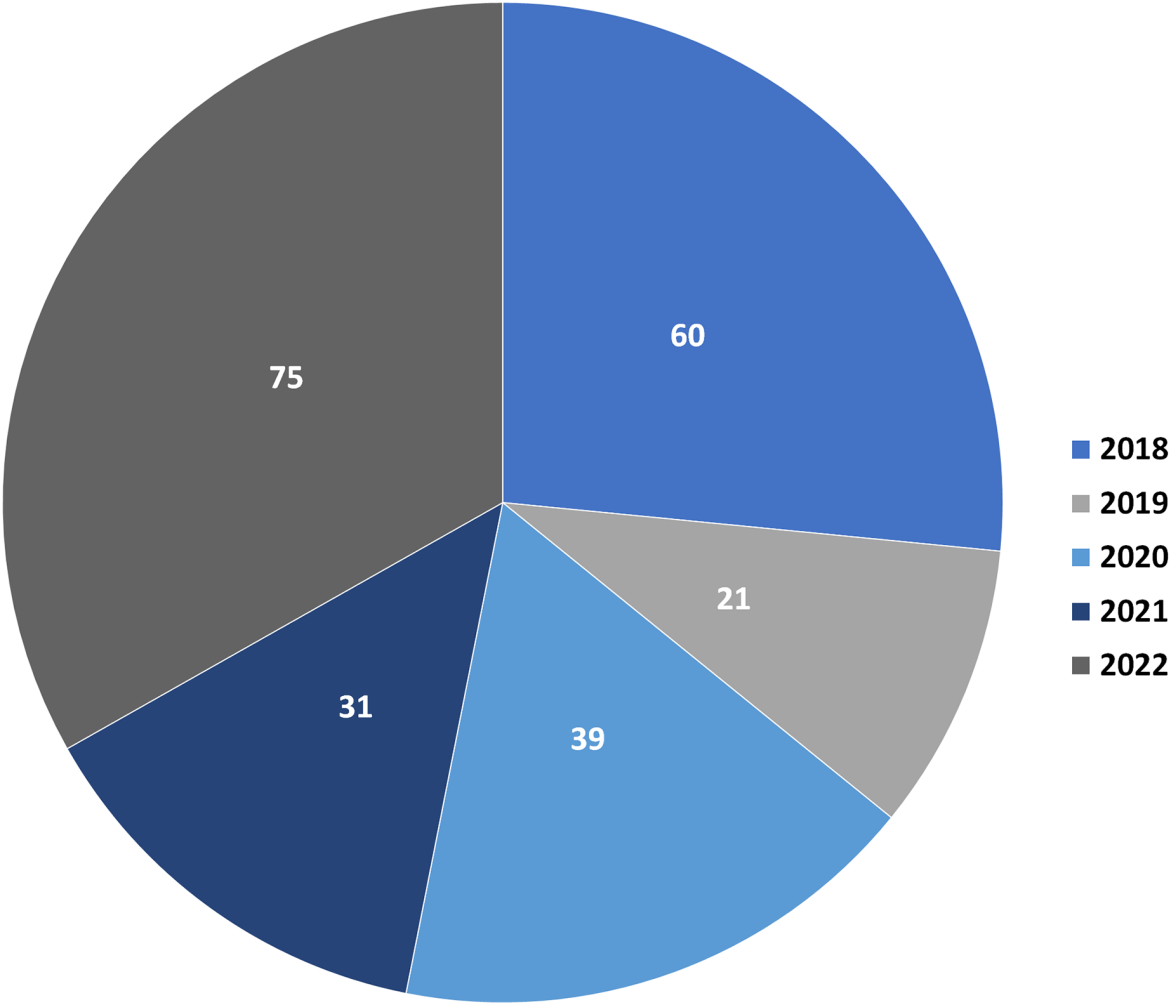
Number of reported depsidones per year in the period from 2018 to 2022.

Depsidones are polyphenolic polyketides featuring a tricyclic framework that have a central seven-membered lactone ring; 11H-dibenzo[b,e][1,4]dioxepin-11-one (Nguyen et al., 2020; Sedrpoushan, Haghi & Sohrabi, 2022). This ring is resulted from ester and ether linkages joining the two ß-orcinol or orcinol-derived rings (Bay et al., 2020; Bai, Yang & Bai, 2021). Biosynthetically, three to seven carbon chains may be connected at C-1 and C-5 of the rings relying on the starting precursor utilized by PKSs (polyketide synthases) to assemble their backbones (Singh et al., 2021). Also, they are proposed to be originated from depsides, which are formed by ester-linking among two orsellinic acid derivatives followed by ether formation (Ibrahim et al., 2018, 2021; Burt, Harper & Cool, 2022). Their biosynthesis had been previously discussed in some reports (Ibrahim et al., 2018, 2021; Singh et al., 2021). Additionally, ring modifications and side chains constitute the characteristic features of different depsidones (Jin et al., 2018; Mathioudaki et al., 2018). Some of the reported derivatives possess halogen atoms, like chloride as a substituent on their skeletons (Duong et al., 2015). Other reported halogenated derivatives were biosynthesized as a result of modification of the culture media using KBr, NaBr, or porcine.NaBr (Guo et al., 2022; Morshed et al., 2018; Sureram et al., 2013). These metabolites were principally encountered in fungi, lichens, and plants and were rarely reported from marine sources (Hartati, Megawati & Antika, 2022; Sepúlveda et al., 2022; Ismed et al., 2021). Naturally occurring depsidones have been reported to display a span of bioactivities including antimicrobial, antimalarial, cytotoxic, anti-*Trypanosoma*, anti-inflammatory, anti-*Helicobacter pylori*, antimycobacterial, antihypertensive, anti-diarrheal, larvicidal, antidiabetic, herbicidal, antileishmanial, phytotoxic, anti-HIV, anti-osteoclastogenic, and butyrylcholinesterase, aromatase, tyrosinase, hyaluronidase, and acetylcholinesterase inhibition (Addo et al., 2021; Ibrahim et al., 2018). These compounds have attracted considerable research interest because of their structural diversity and varied bioactivities. This class of metabolites had been reviewed in some previous works. For example, a review by Ibrahim et al. (2018) discussed the isolation, structural characterization, biosynthesis, and bioactivities of 84 depsidones reported from fungal sources. Additionally, two published reviews by Ureña-Vacas et al. (2022) and Stojanovic, Stojanovic & Smelcerovic (2012) focused on lichen depsidones, including their structures and biological activities. Due to the rapid research growing on these metabolites, the current review is an update to the formerly published review in 2018 (Ibrahim et al., 2018). In addition, the current work focused on the reported depsidones from various sources in the period from 2018 to the end of 2022 as shown in Fig. 1 and Table S1.

**Figure 2:**
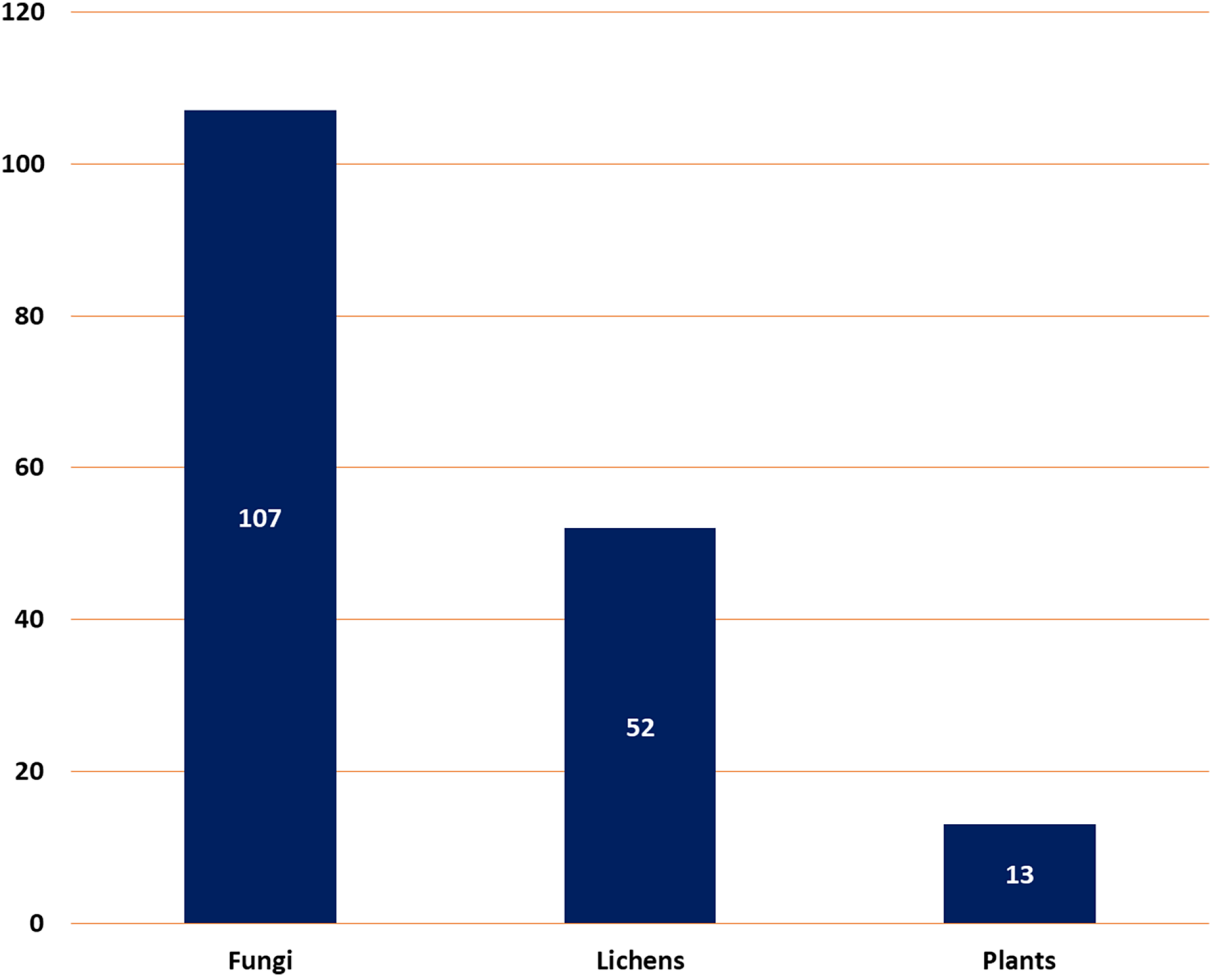
Number of reported depsidones from different sources.

It described a comprehensive summary of the published information on depsidones regarding their sources (fungi, lichens, and plants) (Fig. 2), separation, structural characterization, biosynthesis, semi-synthesis, bioactivities, and structure-activity relation.

This work aimed to provide natural product researchers with comprehensive references that can help them in the identification of separated depsidones from various sources. Additionally, highlighting the reported bioactivities and structure-activity relationship of these compounds may draw the interest of medicinal and synthetic chemists for the synthesis and discovery of new agents utilizing known depsidones as start materials.

## Methodology

The published data on depsidones was obtained by searching articles on various databases and Publishers such as Google-Scholar, PubMed, Science Direct, Bentham, Thieme, Springer, Scopus, Taylor/Francis, and Wiley. The search was done utilizing the keywords: “Depsidone + Lichens”, OR “Depsidone + Fungi”, OR “Depsidone + Plant” OR “Depsidone + Biological activity” OR “Depsidone + Biosynthesis” OR “Depsidone + Semi-synthesis” OR “salazinic acid”, “protocetraric acid”, “lobaric acid”. This work included the English language published articles in peer-reviewed journals in the period from 2018 to the end of 2022. The published articles reported new biological evaluation of metabolites reported before 2018 had been included. A total of 83 articles had been reviewed. The no full access (*e.g*., conference proceedings), irrelevant, and non-reviewed journals published articles had been excluded. For the non-English articles, the data are extracted from the English abstracts.

## Bioactivities of depsidones

The reported depsidones were assessed for various bioactivities (Figs. 3–13, Tables S2–S6).

**Figure 3:**
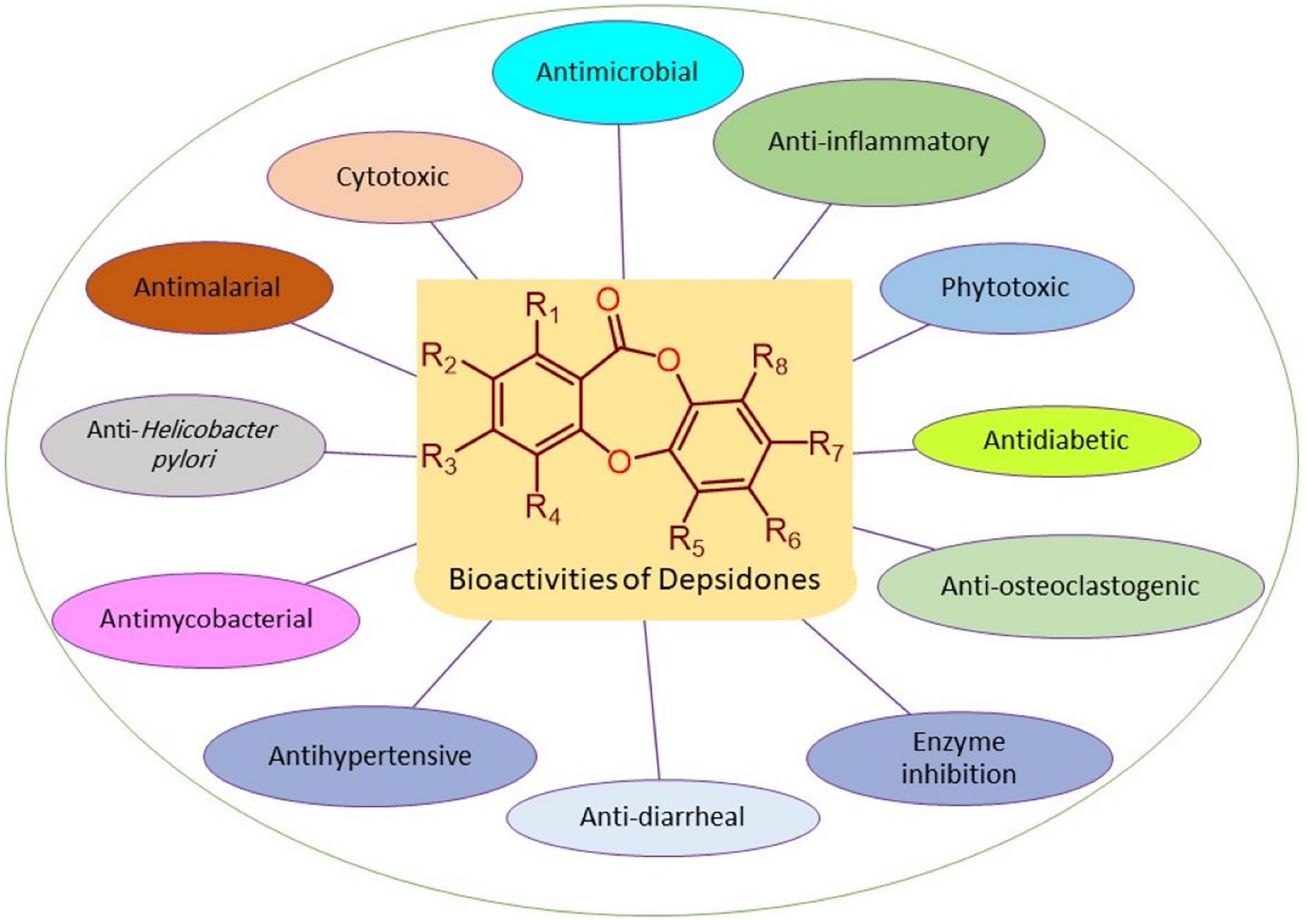
Reported bioactivities of depsidones.

**Figure 4:**
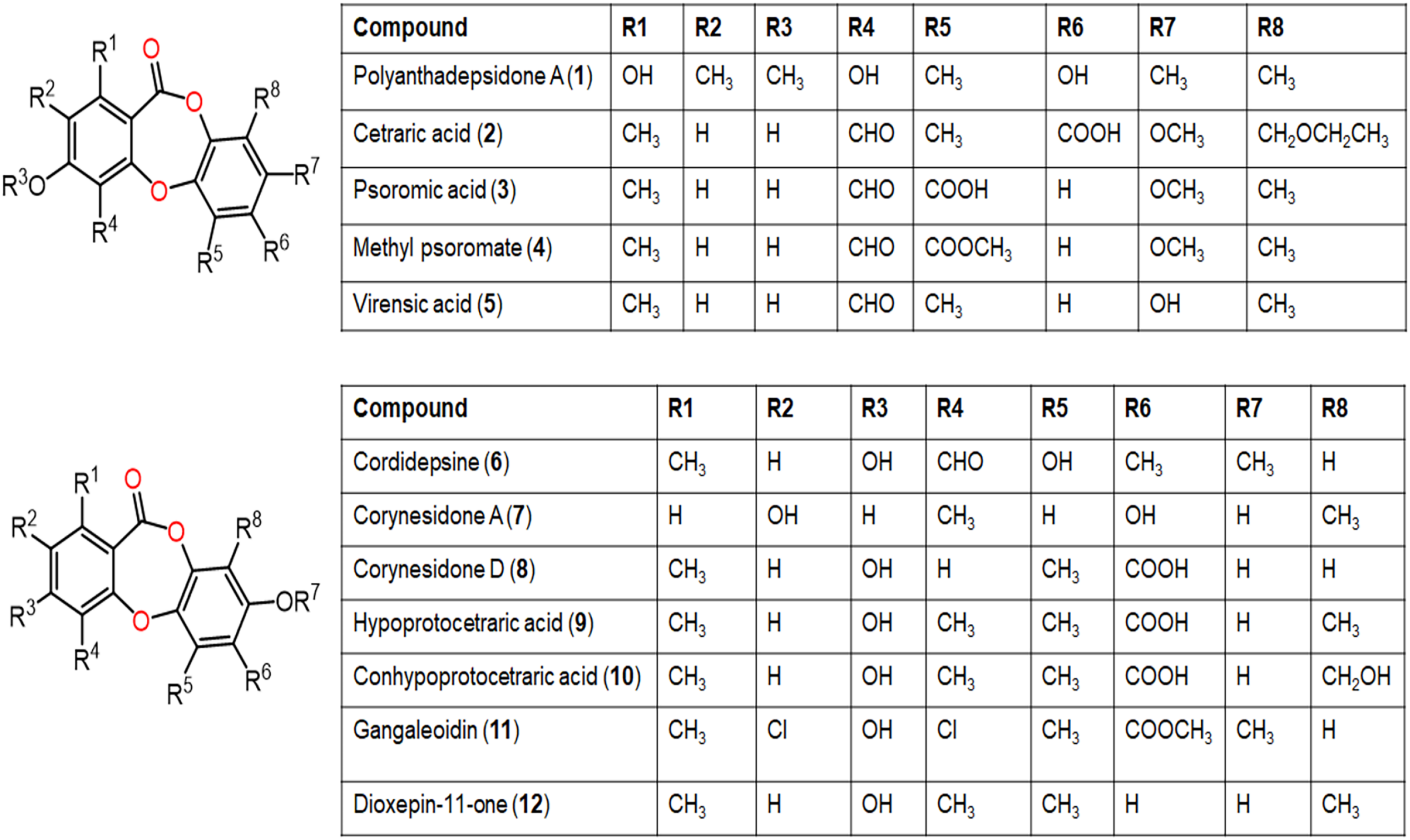
Chemical structures of depsidone (1–12).

**Figure 5:**
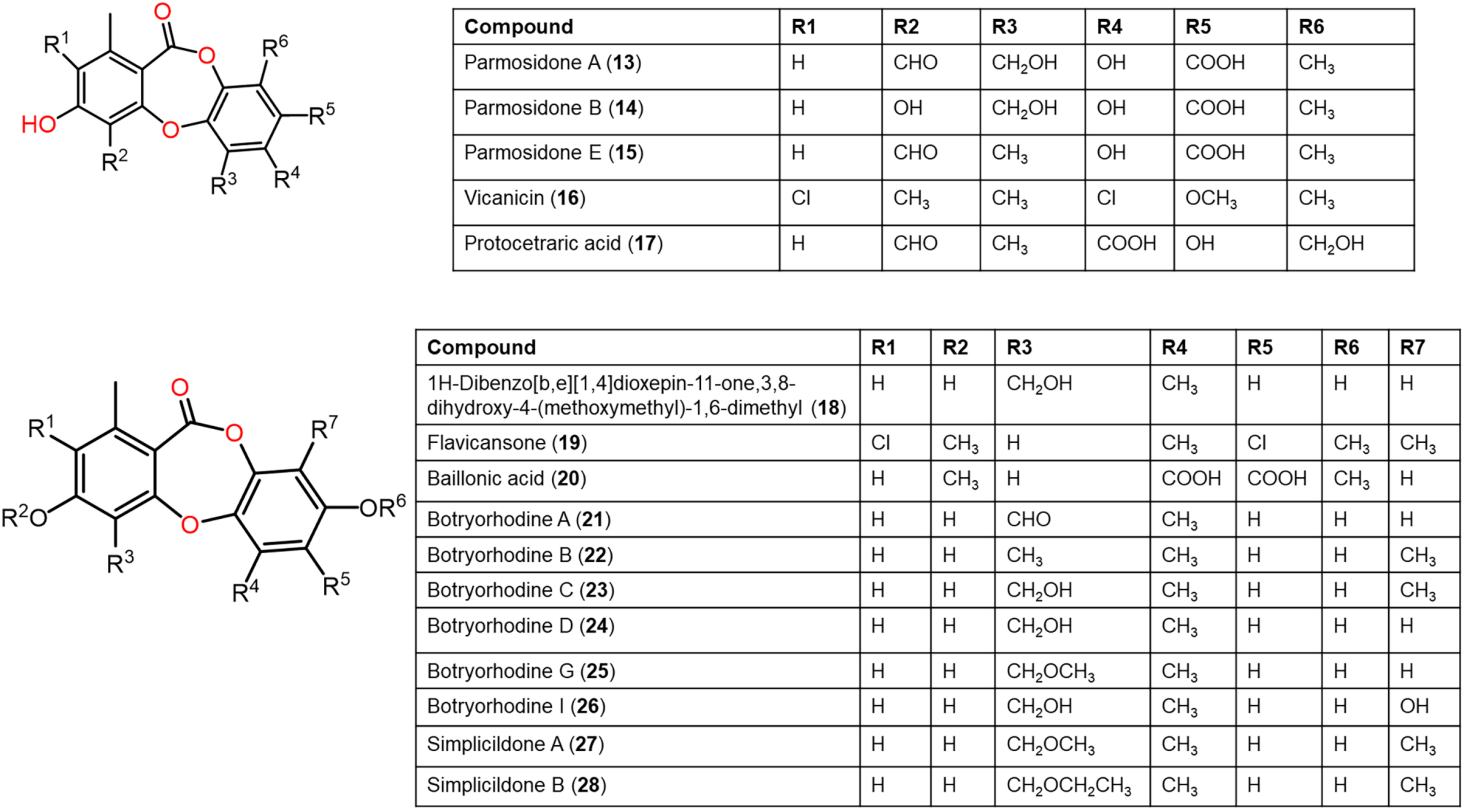
Chemical structures of depsidone (13–28).

**Figure 6:**
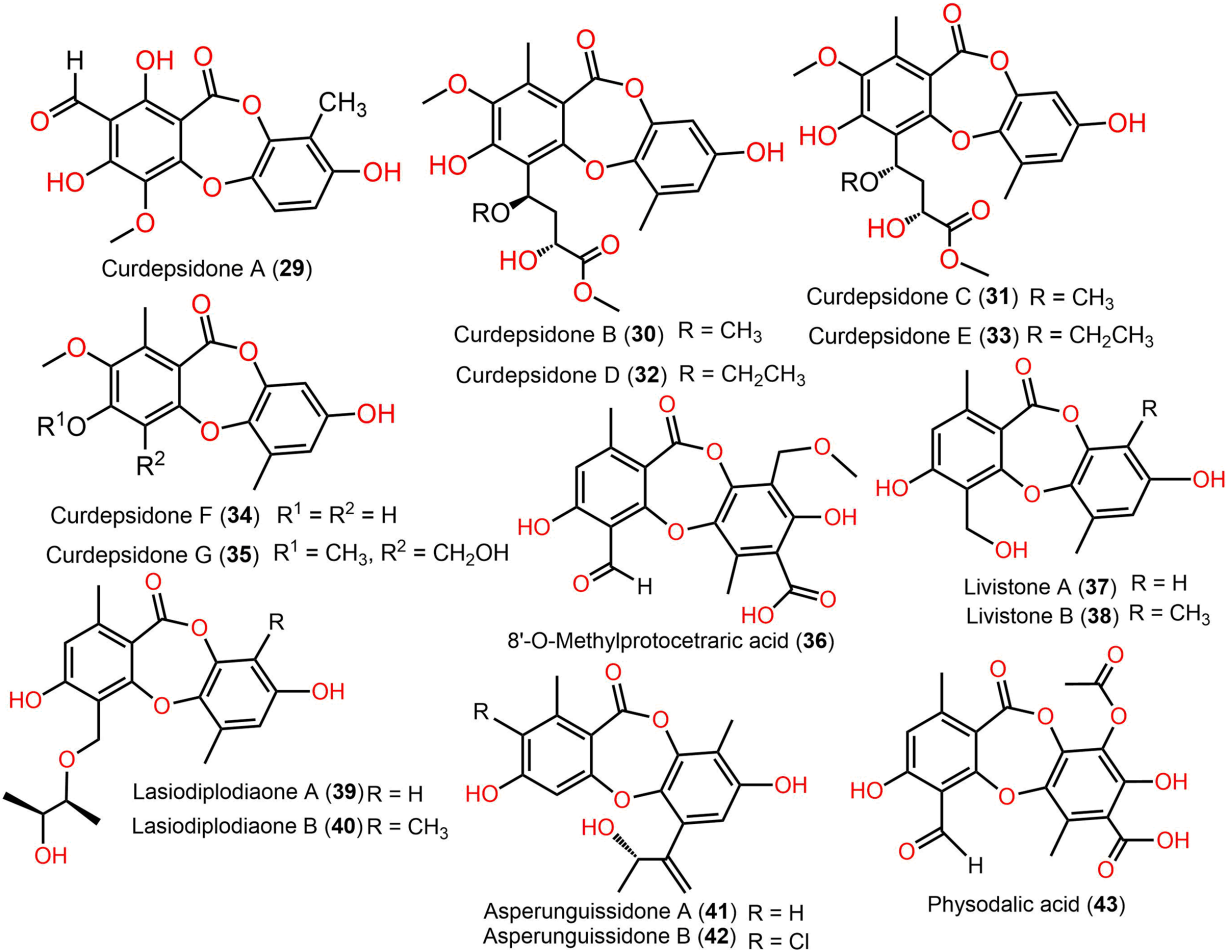
Chemical structures of depsidone (29–43).

**Figure 7:**
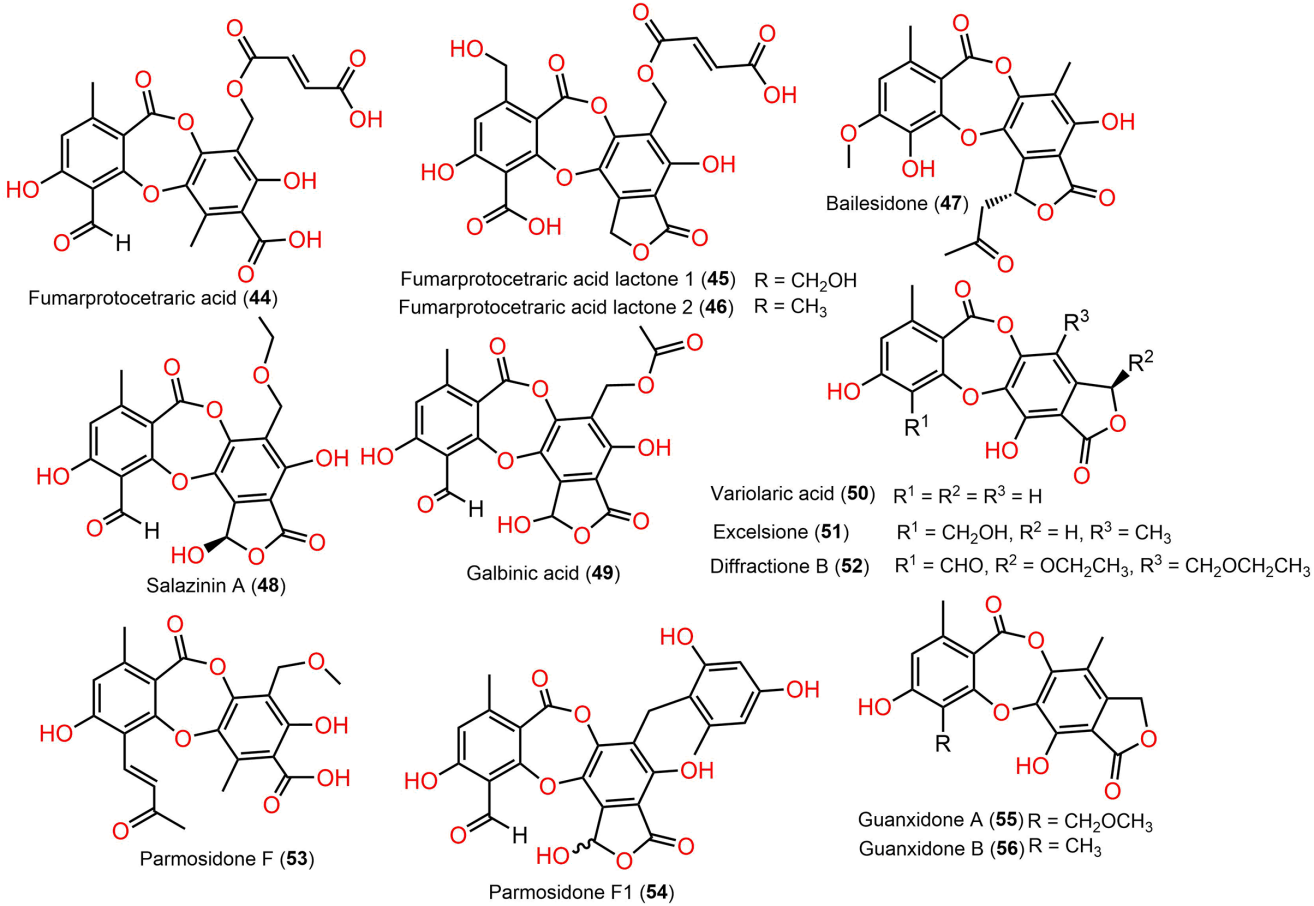
Chemical structures of depsidone (44–56).

**Figure 8:**
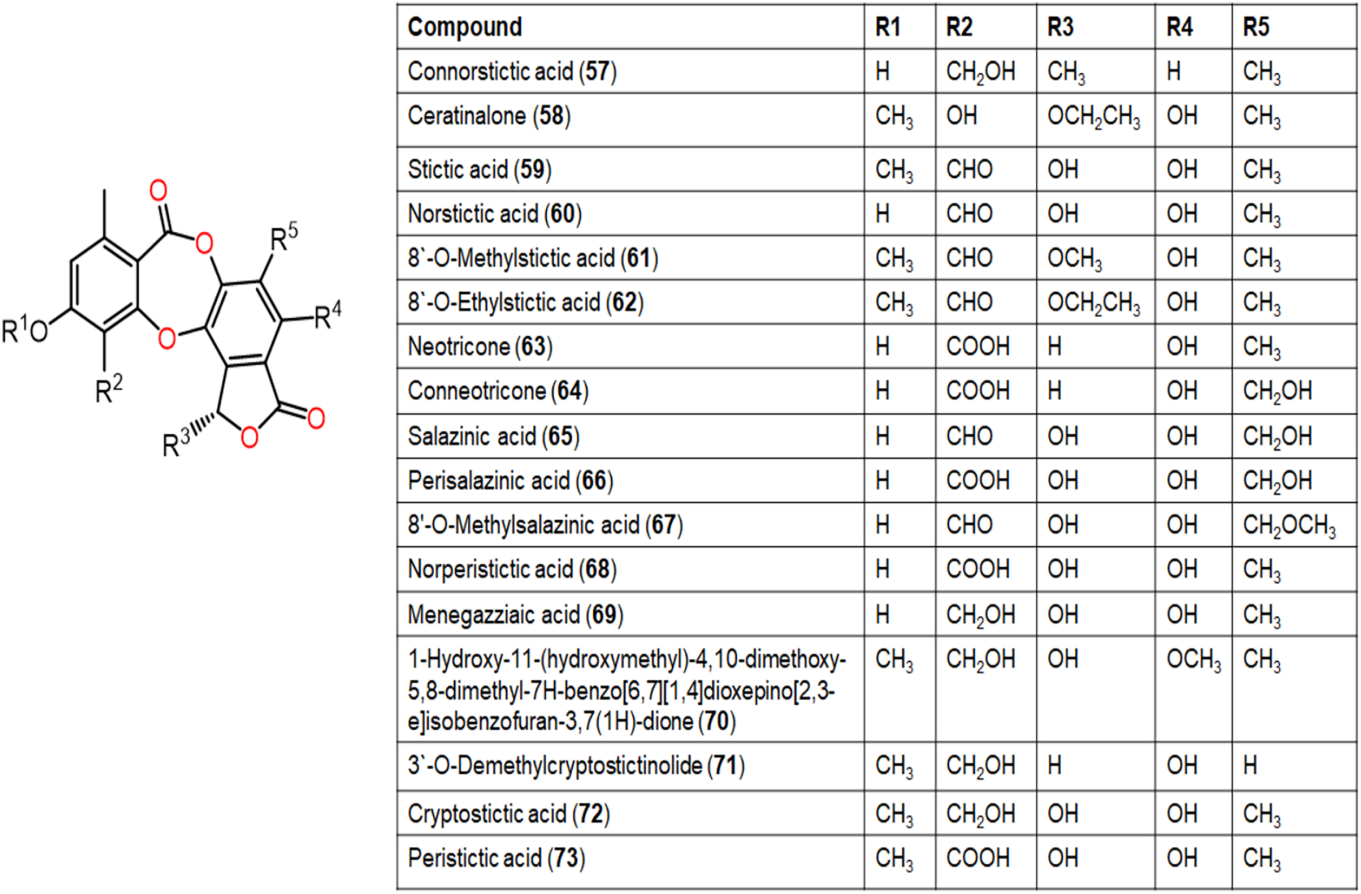
Chemical structures of depsidone (57–73).

**Figure 9:**
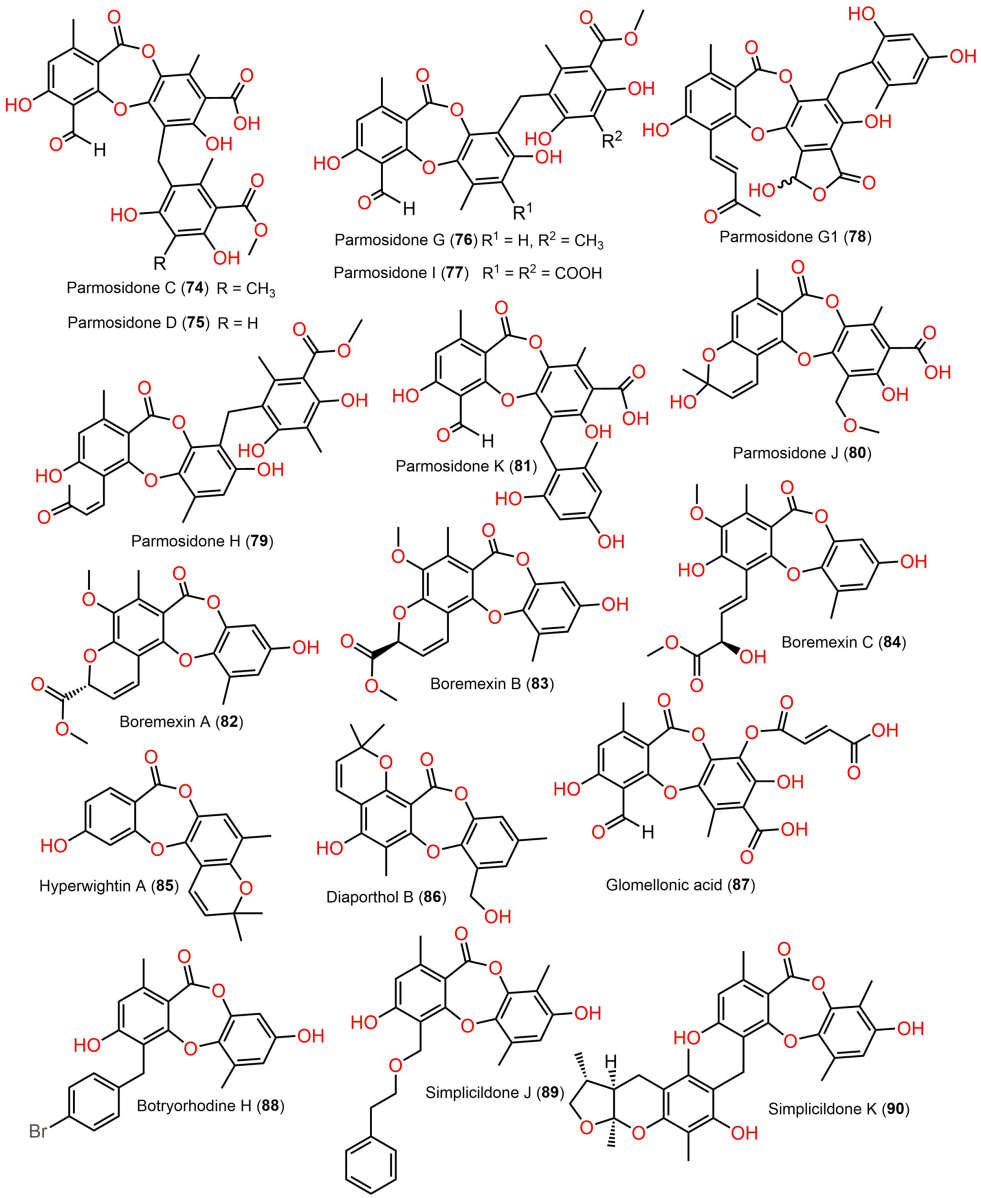
Chemical structures of depsidone (74–90).

**Figure 10:**
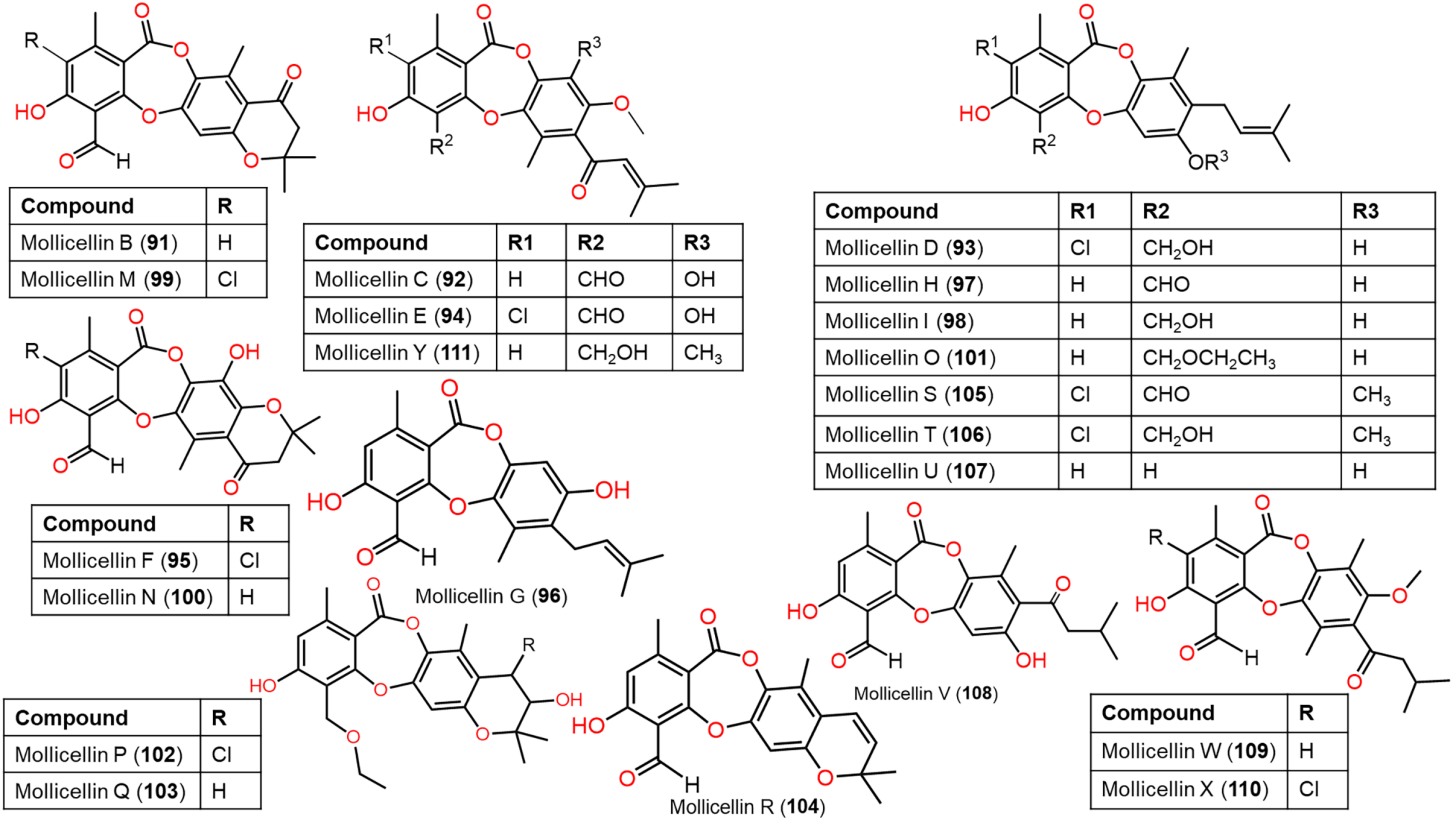
Chemical structures of depsidone (91–111).

**Figure 11:**
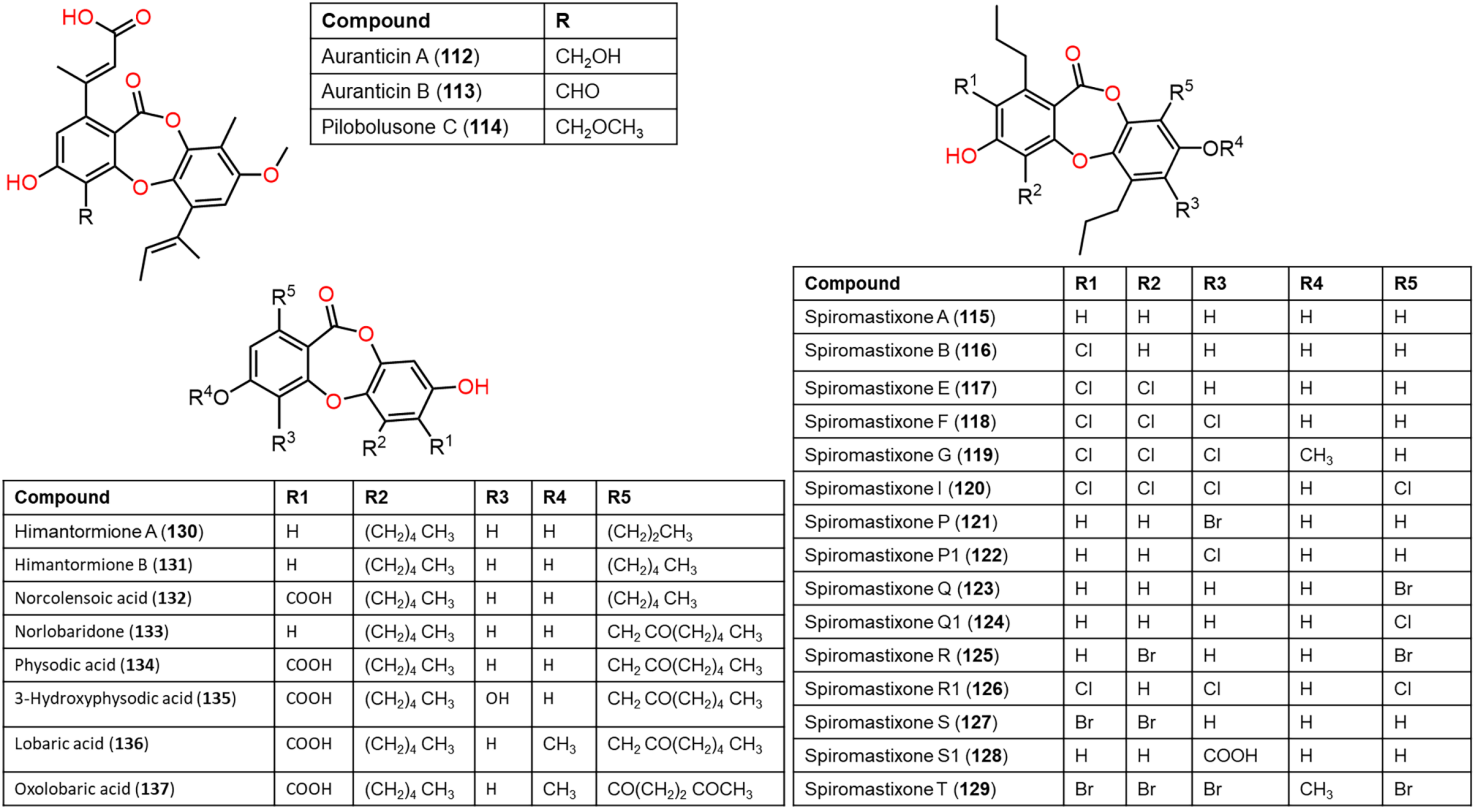
Chemical structures of depsidone (112–137).

**Figure 12:**
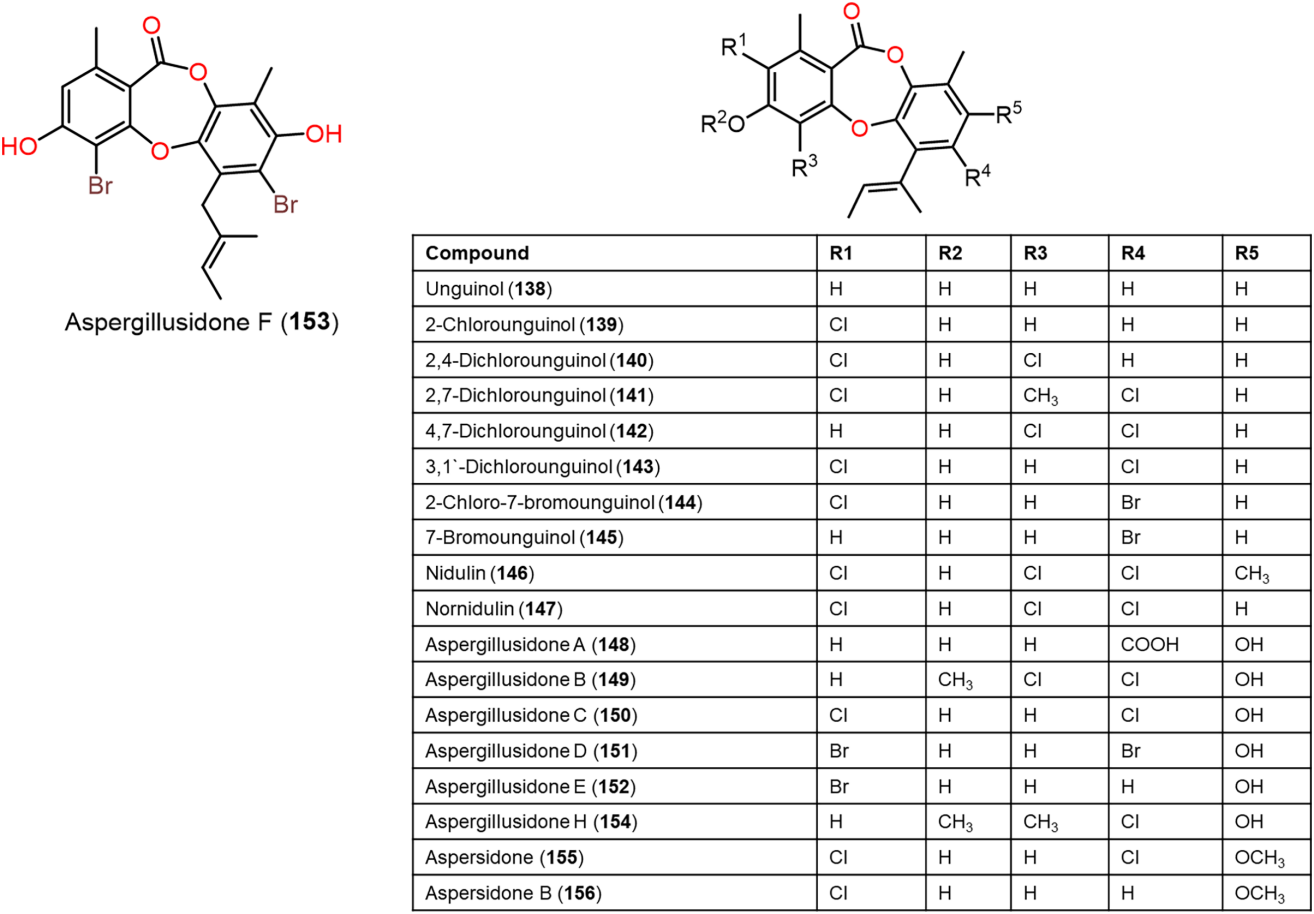
Chemical structures of depsidone (138–156).

**Figure 13:**
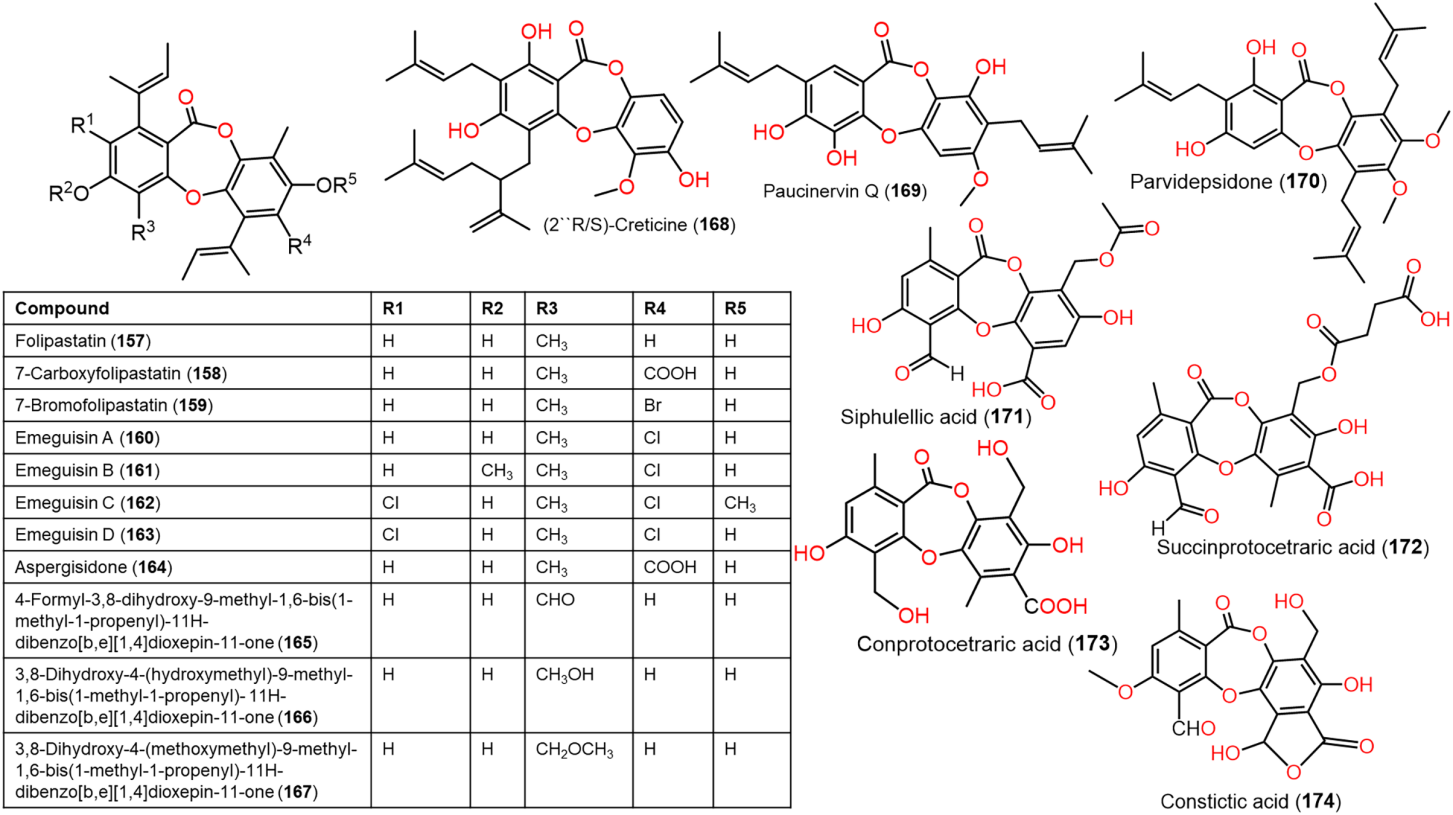
Chemical structures of depsidone (157–174).

It is noteworthy that some of the reported metabolites had more powerful efficacy than the positive controls. The results of the reported bioactivities were listed and discussed below.

### Antimicrobial activity

Currently, antibiotic resistance of microbes has become one of the utmost serious menaces to human health (Fjell et al., 2012). The global amplification and rapid growth of multi-resistant microbes that are untreatable with the current antimicrobial therapy have been associated with growing morbidity and mortality rates (Dhingra et al., 2020). Despite, immense knowledge of this universal health dilemma, developing new-generation antibiotics that combat these microbes has been proven to represent a significant defy (Bahar & Ren, 2013). In this regard, many natural metabolites have gained much attention from scientific and pharmaceutical communities because of their antibiotic potential (Khameneh et al., 2019). The majority of reported depsidones were assessed for their capacities on various pathogens including antitubercular, anti-phytopathogenic, antimalarial, and antibacterial activities. In many studies, they possessed a broad range of activity.

Two new derivatives, simplicildones J (**89**) and K (**90**) and related known compounds **21**, **22**, **27**, and **28** were obtained and characterized from *Simplicillium lanosoniveum* PSUH168 and PSUH261 associated with *Hevea brasiliensis* leaves. Compounds **22** and **27** demonstrated notable antibacterial effectiveness against *Staphylococcus aureus* and MRSA (Methicillin-resistant *S. aureus*, MICs 32.0 μg/mL), while **90** was 4-fold less active compared to vancomycin (MIC 0.5 μg/mL), whilst **90** had (MIC 32 μg/mL) antifungal influence against *Cryptococcus neoformans* ATCC90113 in comparison to amphotericin B (MIC 0.5 μg/mL) (Rukachaisirikul et al., 2019).

From *Eucalyptus exserta-*associated *Chaetomium* sp. Eef-10 cultures, new depsidones, mollicellins O–R (**101**–**104**), in addition to known mollicellins **96**–**98** were purified using SiO_2_, Sephadex LH-20, and HPLC and elucidated by spectral analyses. Among these metabolites, **97** (IC_50_s 5.14 and 6.21 µg/mL, respectively) had notable antibacterial capacity against *S. aureus (*ATCC-29213) and *S. aureus (*N50, MRSA) compared to streptomycin sulfate (IC_50_ 1.05 µg/mL for *S. aureus* ATCC-29213) in the broth dilution assay (Table S2) (Ouyang et al., 2018).

New members of mollicellin family; mollicellins **108**–**111**, together with **92**, **94**, **95**, **97**, **99**, **100**, and **104** were separated utilizing SiO_2_ CC/preparative TLC from Thai rice-accompanied *C*. Brasiliense (Promgool et al., 2022). Compounds **92**–**95**, **108**, and **109** had powerful antibacterial potential against *Bacillus subtilis* and *Bacillus cereus* (MICs 2.0–8.0 μg/mL) which was close to kanamycin (MICs 2.0 μg/mL), however, they displayed moderate efficacy on *S. aureus* ATCC25923 (MICs 16.0–64.0 μg/mL). Besides, **91**, **92**, **97**, **110**, and **111** were moderately active against different MRSA isolates (ATCC33591, ATCC33592, and ATCC43300, MICs 32.0–128.0 μg/mL) with the same MICs as oxacillin (MICs 32.0–128.0 μg/mL), whereas **91** and **92** also had moderate influence (MICs 32.0–128.0 μg/mL) against *S. aureus* SA1-3 clinical isolates (Promgool et al., 2022). It was noted that C-4-CHO and complete lactone ring were significant for antibacterial potential against Gram-positive bacteria (Promgool et al., 2022).

**Scheme 1:**
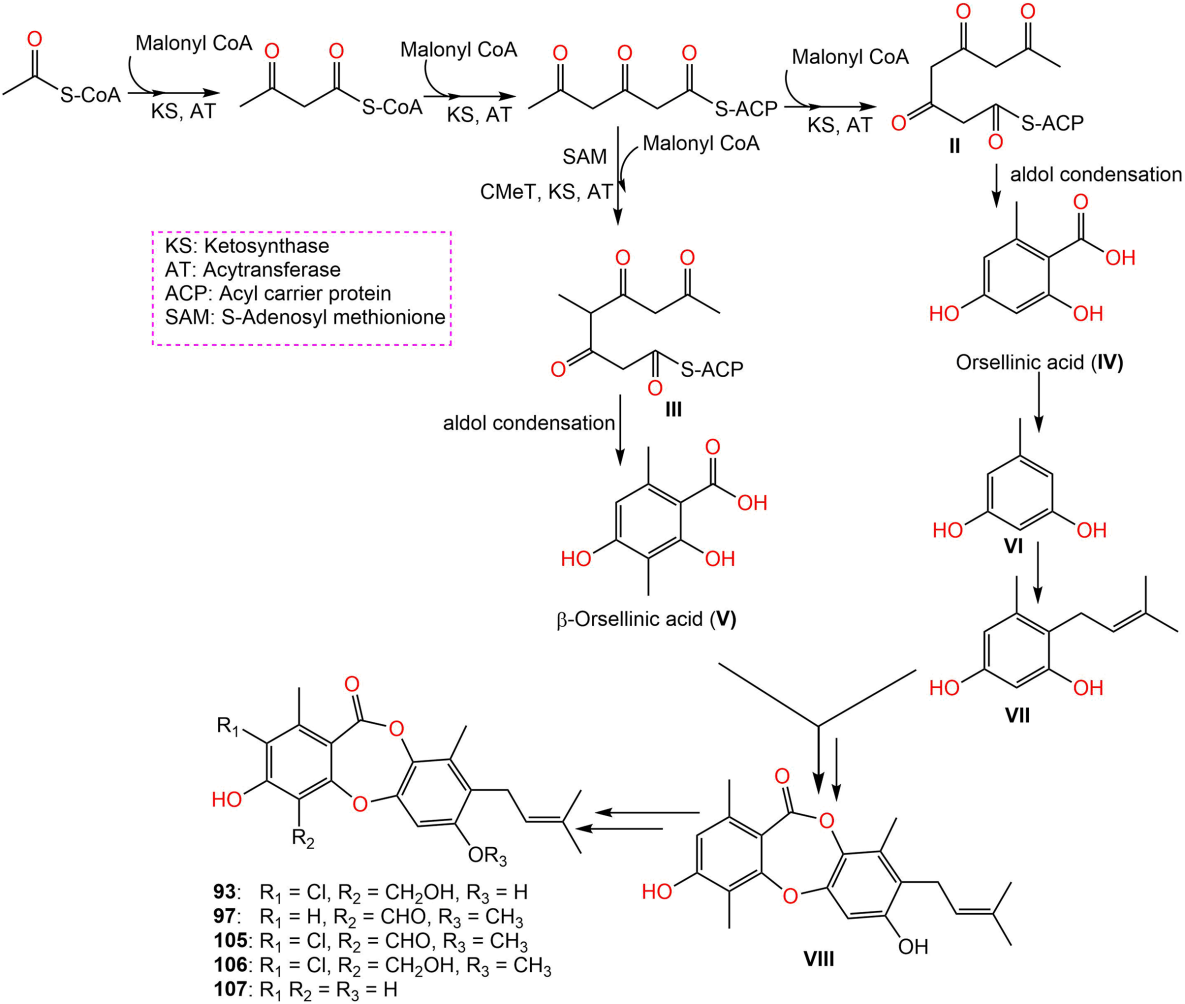
Biosynthetic pathway of 93, 97, and 105–107 (Zhao et al., 2021a, 2021b).

Further, new members of mollicellins: **105**–**107**, and the known mollicellins **93** and **97** were purified from *C. brasiliense* SD-596 rice media utilizing SiO_2_ CC/HPLC and elucidated by spectral analyses (Zhao et al., 2021b). The analogs **97** and **105**–**107** possessed specific inhibition capacities towards *S. aureus* and MRSA, whereas **105** was the most efficient (MICs 6.25 μg/mL) compared to vancomycin (MICs 1.0 μg/mL); however, they had no efficacy against *Candida albicans* and *Pseudomonas aeruginosa* in the broth microdilution assay. It was noted that substituting 6-OH (in **93**) with OCH_3_ (in **106)** and 4-CH_2_OH (in **106**) with 4-CHO (in **105**) enhanced the antibacterial capacity. It is worth noting that CHO at C4 and OCH_3_ at C7 in **105** could have synergetic efficacy in boosting the antibacterial potential (Zhao et al., 2021b). Zhao et al. (2021b) postulated the biosynthetic pathway of these mollicellins as shown in Scheme 1. First, the precursors **II** and **III** were biosynthesized by KS (β-ketoacyl synthase) domain, AT (acyltransferase) domain, ACP (acyl-carrier-protein) domain, and Claisen-type/cyclase-thioesterase domain, in addition to CMeT (C-methyltransferase) domain and SAM (S-adenosyl-methionine) only for **III**. The non-enzymatic aldol condensation of **II** and **III** results in orsellinic acid (**IV**) and β-orsellinic acid (**V**). Decarboxylation of **IV** followed by isoprenylation that are catalyzed by decarboxylase and aromatic prenyl transferase, respectively form **VII**. By oxidase and/or esterase **V** and **VII** are connected to produce **VIII**. Enzymatic oxidation, methylation, or halogenation of **VIII** yield compounds **93**, **97**, and **105**–**107** (Zhao et al., 2021a) (Scheme 1).

From the deep sea-derived *Spiromastix* MCCC3A00308, **122**, **124**, **126**, and **128** were separated and characterized using Sephadex LH-20/HPLC and spectroscopic data, respectively (Niu et al., 2021a). Compounds **122**, **124**, **126**, and **128** revealed promising antibacterial influence on *S. aureus* ATCC25923, *Bacillus thuringiensis* ATCC10792, and *B. subtilis* CMCC63501 (MIC ranging from 0.5–32 μg/mL) compared to chloramphenicol (MIC 1.0 μg/mL), while they were weakly active against *E. coli* in the broth microdilution assay. It was found that the tri-chlorinated derivative, **126** (MICs ranged from 0.5 to 1.0 μg/mL) was more powerful than di-chlorinated analog **124**, whereas the latter had more potential than **122** (Niu et al., 2021a). Guo et al. separated from ant (*Monomorium chinensis*)-associated *Spiromastix* sp. MY-1 cultivated on KBr-supplemented medium, new brominated derivatives; **121**, **123**, **125**, **127**, and **129** and the known depsidones **115**–**120** by SiO_2_ CC/HPLC. These metabolites except **116** and **123** displayed potent growth inhibitory effectiveness (MICs ranged from 5.2–27.6 μM) against plant pathogens; *Xanthomonas oryzae* pv. *oryzae* (Xoo, B1 and B2 strains), *Erwinia amylovora* (B3), *Pseudomonas syringae* pv. *lachrymans* (B4), and *Clavibacter michiganense* subsp. *sepedonicus* (B5), whereas **127** had the strongest potential against *X. oryzae* pv. *oryzae* (MIC 5.2 μM) compared to kanamycin (MICs ranged from 0.54–4.3 μM). *X. oryzae* pv. *oryzae* causes bacterial blight, which is a worldwide devastating rice disease, leading to up to 60% annual yield loss in Asia, it represents one of the utmost fatal rice diseases (Guo et al., 2022; Xu et al., 2015). Mutualistic microbes with ants were reported as effective protection against plant pathogens and herbivores (González-Teuber, Kaltenpoth & Boland, 2014; Guo et al., 2022). These findings revealed that **127** could be a potential lead for bactericides to control rice bacterial-blight disease and the ant-accompanied fungi might be a prominent source of bactericide against rice pathogens in the rice system (Guo et al., 2022). It is noteworthy that compounds **123**, **125**, **127**, and **129** reported by Guo et al. have the same nomenclatures as **122**, **124**, **126**, and **128** isolated by Niu et al. (2021a) however, they have different structures.

From Antarctic lichen *Himantormia lugubris*, two new analogs, himantormiones A (**130**) and B (**131**) were separated and identified by Koo et al. (2022) Compound **130** featured propyl and pentyl units at C-1 and C-8, respectively, while **131** has two pentyl moieties. These compounds exhibited inhibitory capacity on *S. aureus* (IC_50_s 35.09 and 7.01 µM) in the broth microdilution assay. Nugraha et al. (2022) stated that **136** reported from Indonesian lichen *Candelaria fibrosa*, had antibacterial efficacy against *B. cereus and S. aureus* (MICs 88.0 and 39.6 μM, respectively) in the microdilution method.

Furthermore, **139**, **142**, **144**, **145**, **150**–**153**, and **160** reported from *Aspergillus unguis* demonstrated potent antifungal potential against *S. cerevisiae* (ATCC 9763) (MICs 2.3–25.0 µg/mL), compared to clotrimazole (MIC 0.4 µg/mL). Besides, **142**, **144**, **145**, **158**, and **159**, along with earlier reported **138**, **139**, **146**, **147**, **150**–**153**, **157**, and **160** were found to have antibacterial effectiveness against *B. subtilis* and *S. aureus* (MICs 0.8–41.1 µg/mL) in comparison with ampicillin (MIC 0.2 and 3.1 µg/mL, respectively). Whilst **146**, **147**, **157**, **159**, and **160** (MICs 0.8, 1.6, 0.8, 1.6, and 0.8 µg/mL, respectively) revealed the powerful efficacy against *B. subtilis*, also **144**, **151**, **157**, **159**, and **160** with MICs 2.6, 1.6, 1.6, 3.1, and 2.9 µg/mL, respectively were the most active against *S. aureus*. Structure-activity relationship revealed the role of bromo- and/or chloro-substitution in enhancing the potency and maintaining selectivity against bacteria and yeast (Morshed et al., 2018).

**Scheme 2:**
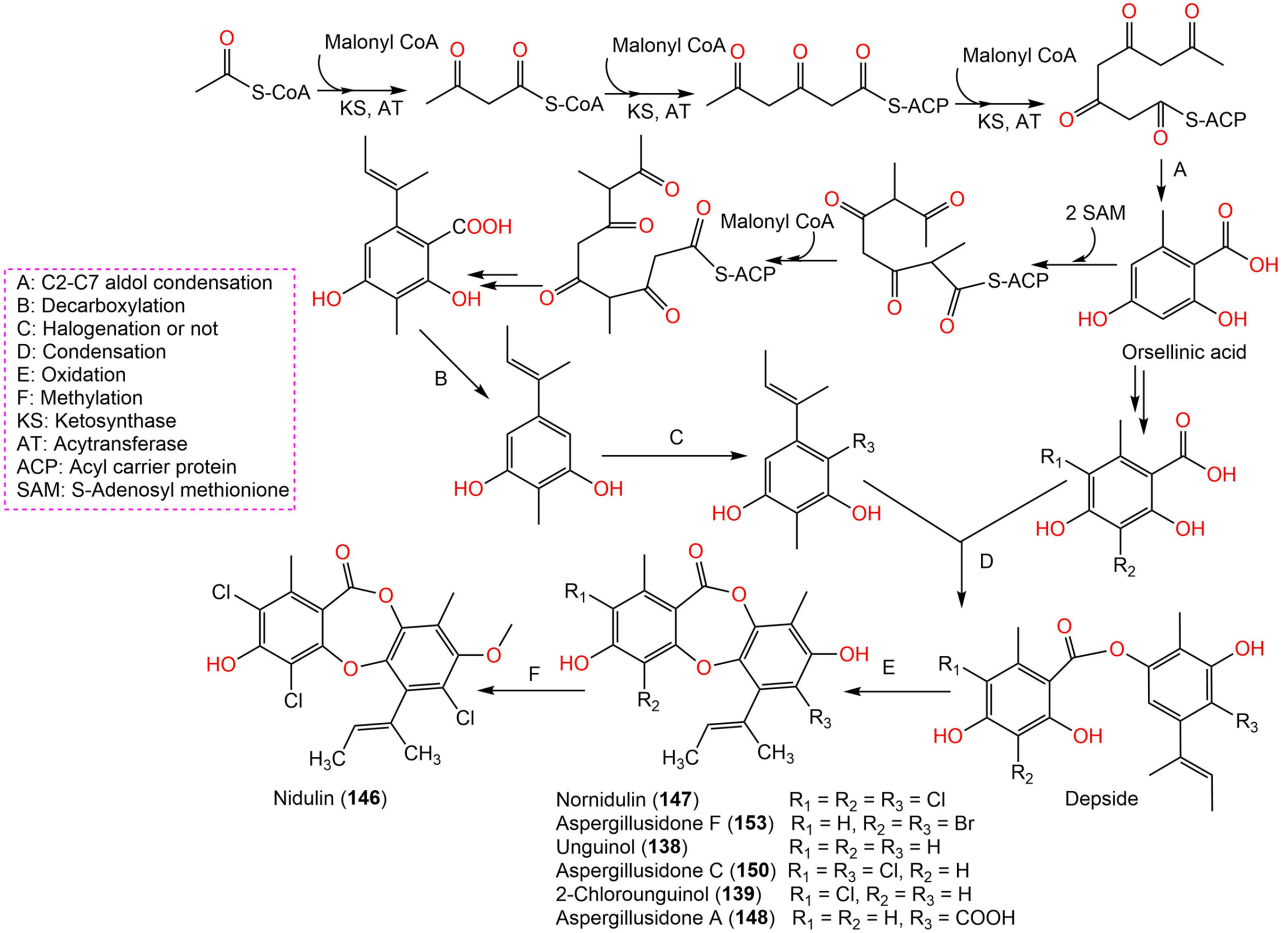
Biosynthetic pathway of 138, 139, 146–148, 150, and 153 (Yang et al., 2018; Sureram et al., 2013).

Aspergisidone (**164**), a new metabolite, along with **138**–**140, 146**–**150**, **155**, **157**, and **160** were obtained from soil-associated *A. unguis* PSU-RSPG204 mycelia and broth EtOAc extract using Sephadex LH-20/SiO_2_ CC/preparative TLC and assigned by spectral analyses (Phainuphong et al., 2018). Compounds **155** and **160** had powerful antibacterial influence against MRSA and *S. aureus* (MICs 0.5 μg/mL) as vancomycin (MICs 0.5 and 0.25 μg/mL, respectively), whereas **139**, **146, 150**, and **157** were weakly active (MIC ranged from 1–8 μg/mL). In the antifungal assay, **139** exhibited potent effectiveness on *C. albicans* (MIC 8 μg/mL), whereas **160** (MICs 0.5 μg/mL) was two-fold more effective than **157** (MIC 1 μg/mL) on flucytosine-resistant *C. neoformans* and **150** had the strongest effect on *Microsporum gypseum* (MIC 2 μg/mL) in the broth dilution method (Phainuphong et al., 2018). Norcolensoic acid (**132**) was purified for the first time from *Lachnum virgineum* using SiO_2_ CC that showed intense blue color with 10% vanillin/H_2_SO_4_ on TLC plates. This compound displayed antimicrobial effectiveness against *Aspergillus clavatus* F318a, *S. aureus* NBRC13276, and *P. aeruginosa* ATCC-15442 (MICs 50, 25, and 25 μg/mL, respectively), however, it was inactive against *C. albicans* (Shiono, Koseki & Koyama, 2018). In Yang et al. (2019) purified depsidone analogs: **138, 139 146**–**148**, **150**, and **153** from plasma-mutant *Aspergillus unguis* or by *A. unguis* in a medium supplemented with epigenetic modifiers (procaine, NaBr, or procaine/NaBr) that were characterized by optical rotation, spectral, and CD analyses. These metabolites had antimicrobial efficacy against *P. aeruginosa*, MRSA, *Vibrio parahemolyticus*, and *C. albicans* (IZDs ranged 6.0 to 17.7 mm, Conc. 10 µg/disc) compared to ampicillin (IZDs 9–14 mm) and ketoconazole (IZD 22 mm for *C. albicans*), whereas **148** demonstrated potent influence on *P. aeruginosa*, MRSA, and *C. albicans*. Structure-activity relationship revealed that the ring C carboxyl group was crucial for antifungal potential (Yang et al., 2018). Also, **138, 139**, **146**–**148**, **150**, and **153** were assumed to be generated through depside production from orcinol derivatives and orsellinic acid that were derived from the PKS pathway and post-PKS modification (Scheme 2) (Yang et al., 2018). In previous work by Sureram et al. (2013), unguinol (**138**) and aspergillusidones D–F (**151**–**153**) were proposed to be biosynthesized through an oxidative coupling of depsides that are produced from the condensation of orcinol derivatives (aspergillusphenols A and B) and orsellinic acid. It is noteworthy these biosynthetic intermediates were co-isolated along with these depsidones (Sureram et al., 2013).

Saetang et al. (2021) purified the new derivatives; asperunguissidones A (**41**) and B (**42**) in addition to **138**, **139**, **146**–**148**, **150**, **157, 160**, **161**, and **164** from *A. unguis* PSUMF16 utilizing SiO_2_/RP-10 CC/preparative TLC. All the 1-methyl-6-(2-methylbut-2-enyl)depsidone derivatives (**41**, **138**, **139**, **146**–**148**, and **150**) possessed remarkable antibacterial effectiveness against *S. aureus* ATCC25923 and MRSA (MICs 1.0–8.0 μg/mL) except for non-chlorinated derivatives; **41**, **138**, and **148** (Phainuphong et al., 2018). Structure-activity relationship revealed that C-4, C-2, and C-7 chlorination dramatically boosted antibacterial capacity. It is noteworthy that the C-4 chlorine atom remarkably elevated antifungal efficacy against *C. neoformans* (*e.g*., **147** and **146** *vs* **150**). Moreover, **41** with 3-substituted-2-hydroxy-3-butenyl unit instead of the 2-methylbut-2-enyl unit in **138** was 2-fold more active against MRSA than **138**, however substituting H-7 (*e.g*., **138**) with C=O group (*e.g*., **148**) led to the loss of antibacterial potential. In the 4-methyl-1,6-di(2-methylbut-2-enyl)depsidone derivatives (**157**, **160**, **161**, and **164**), the non-chlorinated **157** was more potent than **138** against *S. aureus*, MRSA, and *C. neoformans* (MICs 2.0, 1.0, and 1.0 μg/mL, respectively) (Phainuphong et al., 2018), while **164**, the carboxyl derivative of **157** was 16-, 64-, and 64-fold less active against *S. aureus*, MRSA, and *C. neoformans* than **157**. Also, **160** (7-chloro derivative of **157**) demonstrated better potential against *S. aureus*, MRSA, and *C. neoformans* than **157** (MICs 0.5 μg/mL). Besides, lacking **161** (3-methoxy derivative of **160**) antimicrobial potential, indicating 3-OH`s importance for activity (Saetang et al., 2021).

A chemical investigation of marine-derived *A. unguis* EtOAc extracts using RP-18 CC and HPLC resulted in new depsidone, **156** with the known analogs, **138, 139**, **143**, **146**, **147**, **149**, **155**, **157**, and **160** (Anh et al., 2022). Compounds **156** revealed antimicrobial effectiveness against *B. subtilis*, *Micrococcus luteus*, and *S. aureus* (MICs 10.7, 10.7, and 5.3 µM, respectively), compared to kanamycin (MICs 1.0, 0.5, and 1.0 µM, respectively) in the broth dilution assay (Anh et al., 2022).

From coral-derived *A. unguis* GXIMD-02505, new metabolite; aspergillusidone H (**154**), along with **147**, **149,** and **150** were purified by Zhang et al. (2022) using SiO_2_/RP-18/HPLC and determined by spectral and physicochemical data. These compounds had inhibitory potential against marine biofilm-producing bacteria; MRSA, *Marinobacterium jannaschii*, *Microbulbifer variabilis*, and *Vibrio Pelagius* in the broth microdilution method. It is noteworthy that **147** possessed significant effectiveness against MRSA (MIC 2.0 µg/mL) compared to ampicillin (MIC 1.0 µg/mL). Besides, **147** and **150** exhibited moderate efficacies against *M. variabilis* and *M. jannaschii* (MICs ranged from 8.0–32.0 µg/mL) (Zhang et al., 2022).

Sadorn et al. (2022) reported a new metabolite; **163**, in addition to **8, 138, 139, 141, 142, 146**–**150**, **157,** and **160**–**164** from *Coriandrum sativum*-associated *A. unguis* BCC-54176 using Sephadex LH-20 CC/HPLC that were assigned by spectral analyses and chemical transformation, as well as X-ray data for **163**. Compounds **8, 139**, **141**, **146**, **147**, **149, 150**, and **160**–**164** displayed broad antibacterial effectiveness against *B. cereus* (MICs 1.56–25.00 μg/mL), while **163** had the potent efficacy (MIC 1.56 μg/mL) compared to rifampicin and vancomycin (MICs 0.31 and 0.08 μg/mL, respectively) (Sadorn et al., 2022). Assessing the anti-phytopathogens activity of these metabolites revealed that **139**, **147**, **150**, and **164** were active (MICs 6.25–50 μg/mL) on *Alternaria brassicicola* and **139**, **141**, **146**–**148**, **150**, **160**, and **164** demonstrated anti—*Colletotrichum acutatum* (MICs 3.13–50.00 μg/mL) using CFDA (5(6)-carboxyfluorescein diacetate) fluorometric assay compared to amphotericin B (MIC 1.56 μg/mL). Whilst all of them did not possess any effect on *A. baumannii* (Conc. 50 μg/mL) (Sadorn et al., 2022). Structure-activity relation showed that metabolites with two (E)-1-methylprop-1—enyl units at C-6 and C-1 (*e.g*., **157** and **160–164**) and the ones with C-7 Cl-atom (*e.g*., **160**) had more potential against *B. cereus* than the non-chlorinated analog (*e.g*., **157**). On the other hand, compounds with C-2 and C-7 two Cl-atoms had better activity (**163** and **162**) than the compound with one Cl-atom (as in **160**). In addition, the C-3 methoxy group led to the loss of activity (**160** *vs* **161**), while the C-7 carboxy boosted the efficacy (**157** *vs* **164**). Further, more (E)-1-methylprop-1—enyl moiety and Cl-atoms in the compounds led to more activity (Sadorn et al., 2022).

### Antimalarial and antimycobacterial activities

Compound **44** purified from *Cladonia pyxidata* was found to have marked growth inhibitory potential against *Mycobacterium tuberculosis* H37Ra and six MDR (multidrug-resistant) *M. tuberculosis* clinical isolates with MICs 7.81–31.25 μg/mL, compared to rifampicin (MICs 0.2–100 μg/mL) using the XRMA method (Thuan et al., 2022). Further, **65** and **136** reported from *Usnea laevis* possessed potent antimycobacterial capacity against MDR strains of *Mycobacterium smegmatis* (MDR-40 and MDR-R) (MICs 50 μg/mL) than rifampicin (MICs 100 and >200 μg/mL, respectively), also **136** had potent (MICs 50 μg/mL) efficacy towards *M. tuberculosis* (MDR-A8 and MDR-V791) compared to rifampicin (MICs 100 and >200 μg/mL, respectively) (Tatipamula & Annam, 2022).

Among the reported derivatives; **8**, **138**, **139**, **141**, **142, 146**–**150**, **157**, and **160**–**164** from *C. sativum*-associated *A. unguis* BCC-54176, **149**, **157**, and **160**–**163** revealed anti*-Plasmodium falciparum* (K1, MDR-strain, IC_50_s 7.69–9.02 μM) in the micro-culture radio-isotope assay compared todihydroartemisinin (IC_50_ 2.60 nM) and chloroquine (IC_50_ 0.51 μM) (Sadorn et al., 2022) (Table S3), whilst **138**, **139**, **150, 160**, **162**, and **163** exhibited anti-*Mycobacterium tuberculosis* (MICs 15.0–50 μg/mL) relative to ofloxacin, rifampicin, streptomycin, ethambutol, and isoniazid (MICs 0.39, 0.01, 0.31, 0.94, and 0.05 μg/mL, respectively) in the GFPMA (green fluorescent protein microplate assay) (Sadorn et al., 2022).

### Cytotoxic activity

Some of the reported depsidones were assessed for their cytotoxic capacities against different cancer cell lines that were highlighted below, and the results of the potential metabolites were listed in Table S4.

Flavicansone (**19**) a 2,7-dichloro-3,8-dimethoxy-1,6,9-trimethyl-11H-dibenzo[b,e][1,4]dioxepin-11-one was separated as a new metabolite, along with **16** from *Teloschistes flavicans* lichen utilizing SiO_2_ CC and Sephadex LH-20 that were specified by different spectral tools. Compound **19** is structural like **16** with differences in substitution at C-3 and C-4, having 3-OCH_3_ instead of 3-OH in **16** and lacking 4-CH_3_ in **16**. Compound **19** possessed moderate cytotoxic effectiveness against HL-60 cells in the CCK-8 assay (IC_50_ 58.18 μM) compared to quercetin (IC_50_ 61.1 μM) and 5-fluorouracil (IC_50_ 9.5 μM) (Sanjaya et al., 2020). Botryorhodine I (**26**) was reported as a new derivative, along with **18**, **21**, **22**, **24**, and **27** from sediment-obtained *Lasiodiplodia theobromae* M4.2-2 rice cultures using Sephadex LH-20 CC and HPLC. Only **18** possessed noticeable cytotoxic potential (IC_50_ 7.3 μM) on L5178Y compared to kahalalide F (IC_50_ 4.30 μM) in the MTT assay, while other metabolites were inactive (Umeokoli et al., 2019). The cytotoxic effectiveness of **23**–**25** and **88** against MMQ and GH3 cells showed that **88** had potent cytotoxic potential against GH3 and MMQ cell lines (IC_50_ 3.64 and 3.09 μM, respectively), while **23 (**IC_50_s 31.62 and 19.72 μM, respectively) displayed moderate effectiveness and **24** and **25** were inactive in the MTT assay (Zhang et al., 2018).

A new depsidone, curdepsidone A (29) purified from white croaker-associated curvularia sp. IFB-Z10 EtOAc extract by macro-porous resin CC and HPLC and assigned by spectral analyses. It displayed marked cytotoxic efficacy against BEL7402 and BEL7402/5-Fu (IC_50_s 9.85 and 2.46 μM, respectively), compared to 5-fluorouracil (IC_50_s 14.0 and 1,630.0 μM, respectively) in the MTT assay (An et al., 2018). A novel depsidone, bailesidone (**47**), which is an 8`S-configured analog of **69** with unparalleled B-ring substituents was biosynthesized by *Usnea baileyi*. This metabolite had moderate potential against the A549 cell line (IC_50_ 92.94 µM) and no influence against the HT-29 cell line (Van Nguyen et al., 2018). Bui et al. (2022) purified and characterized a new derivative; ceratinalone (**58**) along with **47**, **59**, **61**, and **62** using SiO_2_ CC and spectral data. Compounds **58** and **61** were moderately cytotoxic against MCF-7, HeLa, HepG2, and NCI-H460 in the SRB assay. Besides, **61** revealed a notable influence against HeLa cells (IC_50_ 15.61 μg/mL) (Ouyang et al., 2018). Compounds **65** and **134** demonstrated high cytotoxic efficacy against DLD-1 and HCT116 cells through modulation of NF-κB, Nrf2, and STAT3 pathways. It was found that **65** was the most potent modulator of these pathways (Papierska et al., 2021).

The new depsidones: boremexins A–C (**82**–**84**), in addition to **7**, **30**, **31**, and **34** were biosynthesized by *Boeremia exigua* harboring potato that were separated and specified utilizing SiO_2_/RP-18 CC/HPLC and spectral/ECD analyses, respectively. Compounds **82** and **83** were obtained as a racemic mixture having 10*R* ([*α*]_D_ + 199.2) and 10*S* ([*α*]_D_ − 206.5) configurations, respectively that were further separated into enantiomers on chiral HPLC column. In the MTT assay, **83** (IC_50_ 33.1 μM) possessed cytotoxic capacity against MCF–7 compared to taxol (IC_50_ 0.008 μM) (Chen et al., 2020).

Nakashima et al. (2018) purified **86** from *Phellodendron amurense*-associated *Diaporthe* sp. ECN.137 culture by SiO_2_ CC, which was assigned by spectral and X-ray analyses. Its effect against TGFβ1, which boosts the tumor cell invasion was examined. It was found (Conc. 20 µM) to repress TGFβ1-caused wound closing of MDA-MB-231 cells, indicating its possible potential as a tumor metastasis inhibitor (Fig. 9). On the other hand, **96** revealed prominent cytotoxic efficacy against HepG2 and HeLa cells (IC_50_s 19.64 and 13.97 µg/mL, respectively), whereas **97** had better cytotoxic potential on HepG2 (IC_50_ 6.83 µg/mL) in comparison to camptothecin (IC_50_s 3.6 and 6.3 µg/mL, respectively) in the MTT assay (Ouyang et al., 2018).

A study by Promgool et al. (2022) showed that mollicellins **91**, **92**, **94**, **95**, **97**, **99**, **100**, **104**, and **108**-**110** exhibited cytotoxic efficacy against Hela, KB, HepG2, MCF-7, and HT-29 cell lines (IC_50_s 4.79–92.11 μM), where **95**, **97**, and **110** (IC_50_s 4.79, 10.64, and 9.83 μM, respectively) and **91**, **95**, and **110** (IC_50_s 10.66, 7.10, and 11.69 μM, respectively) were potent against KB and HepG2 cell lines, respectively. These metabolites were cytotoxic on Vero cells (IC_50_s 5.65–54.06 μM) except **111**. The findings indicated that the complete lactone ring and C-4-CHO group were substantial for activity, whereas the replacement of C-4-CHO with CH_2_OH resulted in the loss of activity (*e.g*., **111**) (Promgool et al., 2022).

Also, Koo et al. (2022) reported that in the MTS assay of **130** and **131** against HCT-116 cells, **131** showed potent cytotoxic potential (EC_50_ 1.11 μM) than 5—fluorouracil (EC_50_ 9.4 μM), suggesting its potential as an anticancer lead against colon cancer. Additionally, physodic acid (**134**) identified from *Hypogymnia physodes* European lichen (Fig. 11) was found to exhibit cytotoxic potential against A-172, T98G, and U-138 MG cell lines (IC_50_s 42.41, 50.57, and 45.72 μM, respectively) in the MTT assay (Studzińska-Sroka et al., 2021). Additionally, Cardile et al. investigated **134**`s potential on DU-145 and LNCaP cell growth and its apoptotic capacity on TRAIL-resistant LNCaP cells in combination with TRAIL (tumor-necrosis factor-related apoptosis-inducing ligand) using MTT assay. Lactate dehydrogenase (LDH) release is a marker of membrane breakdown. It prohibited both cell viability (Conc. 12.5–50 μM) without affecting normal cells and no observed increase in LDH (lactate dehydrogenase) level, which is a marker for membrane integrity. In addition, it activated apoptosis and raised ROS formation. Interestingly, it sensitized LNCaP cells to TRAIL-produced apoptosis. Thus, combining **134** with other anti-prostatic cancer drugs could be a prominent treatment strategy that required further studies (Cardile et al., 2022).

Anh et al. (2022) stated that **138**, **139**, **143**, **146**, **147**, **155**–**157**, and **160** were found to have cytotoxic potential against PC-3, NCI-H23, HCT-15, NUGC-3, ACHN, and MDA-MB-231 with IC_50_s ranging from 3.4 to 27.7 µM, whereas **138**, **139**, and **143** were the most potential metabolites (IC_50_s 3.4–6.2 µM). It was observed that the number of chlorine and substitution had no significant effect on activity, while free C-4-OH (**157**) was substantial for activity.

Zwartsen et al. (2019) reported that **138** and **151** reduced MDA-MB-231 cell viability (Conc. M50 μM), while they did not affect cell proliferation (Fig. 12). Additionally, they caused MDA-MB-231 cell cycle arrest (Conc. 100 μM). It is noteworthy that **138** potency was less than **151**, this variation may be due to two bromine atoms in **151** compared to **138** that enabled the halogen bonds formation (Fig. 12).

New depsidones: **142**, **144**, **145**, **158**, and **159**, along with earlier reported **138**, **139**, **146**, **147**, **150**-**153**, **157**, and **160** were biosynthesized by *A. unguis* using yeast extract sucrose culture media supplemented with KBr or NaCl (Morshed et al., 2018). In the MTT assay, they demonstrated cytotoxic potential against NS-1 cell line (MICs 6.3 to 50 µg/mL) compared to 5-fluorouracil (MIC 0.1 µg/mL), whereas **138**, **157**, **159**, and **160** (MICs 12.5, 6.3, 12.5, and 12.5 µg/mL, respectively) were the most active (Morshed et al., 2018).

Phainuphong et al. (2018) reported that **160** revealed the potent inhibition activity on HCT-116 cell (IC_50_ 23.5 μM, inhibition 87.06%), while **138**–**140**, **146**–**150**, **155**, **157**, and **164** had weak to moderate efficacy (3.98–59.63%). Compound **160** also dose-dependently decreased (IC_50_s 34.8–84.7 μM) live cells/dead cells numbers in a 3D-culture model relying on the incubation durations, indicating its potential in spheroidal cancer model (Phainuphong et al., 2018). Further, compounds **146**, **147**, and **153** demonstrated notable larvicidal potential on *Artemia salina* (LC_50_s 4.5–12.8μM) compared to Hg(NO3)_2_ (LC_50_ 77.0 μM) (Yang et al., 2018).

Compounds **112**–**114** and **165**–**167** separated from the culture of wetland-soil-associated *Pycnidiophora dispersa*, utilizing SiO_2_/RP-18 CC/HPLC had cytotoxic capacity against HeLa, PC-3, A549, HepG-2, and HL-60 (IC_50_s ranged from 11.4 to 86.8 μM) compared to cisplatin (IC_50_s ranging from 5.6 to 15.7 μM) in the CCK-8 assay. Compounds **112** and **165** had marked efficacy on A549 cells (IC_50_s 13.0 and 11.4 μM, respectively) compared to cisplatin (IC_50_ 11.8 μM) (Zhao et al., 2020).

From *Garcinia paucinervis* stems, a new depsidone, paucinervin Q (**169**) was separated by SiO_2_ and RP-18 CC and assigned by spectral analyses. This compound revealed marked inhibition capacity against PC-3, HL-60, and CaCo-2 (IC_50_s 18.57, 3.11, and 6.78 μM, respectively) in the MTT assay compared to 5-fluorouracil (IC_50_s 30.59, 2.39, and 38.77 μM, respectively) (Jia et al., 2019) (Fig. 13).

### Anti-inflammatory activity

Inflammation is a complicated defense process, which is induced by pro-inflammatory cytokines secretion by macrophages as a result of stimuli (*e.g*., infectious agent, tissue ischemia, injury, *etc*.) (Zhao et al., 2021a; Liang et al., 2022). Impairment of the pro-inflammation mediator secretion can lead to diverse disorders such as asthma, atherosclerosis, psoriasis, periodontal diseases, carcinogenesis, and rheumatoid arthritis (Chen et al., 2018a; Niu et al., 2021b).

Also, polyanthadepsidone A (**1**), a new highly methylated depsidone from the *Garcinia polyantha* leaves dichloromethane extract exhibited *in vitro* suppressive influence on the oxidative burst by serum opsonized zymosan in the whole blood (Lannang et al., 2018).

Chemical investigation guided by HPLC/DAD of the EtOAc extract of the marine-derived *Curvularia* sp. IFBZ10 resulted in new depsidones; **30**–**35** that were separated by SiO_2_ CC/HPLC and their structures and absolute configuration were determined by spectral analyses as well as TDDFT/ECD (time-dependent density functional theory/electronic circular dichroism) and DFT/NMR (density functional theory/nuclear magnetic resonance) calculations (Duong, 2019). The anti-inflammation potential of **30**, **31**, **34**, and **35** was assessed by measuring IL-1β production inhibition in *Propionibacterium acnes*-induced THP-1 cells. Compound **31** exhibited noticeable IL-1β production inhibition (IC_50_ 7.47 μM) compared to retinoic acid (IC_50_ 3.38 µM), while **30** and **35** (IC_50_ 18.83 μM) had no and moderate efficacy, respectively revealing that stereo-configuration had a substantial role in the activity. Further, **31** prohibited the IL-1β production by selectively minimizing the JNK and ERK phosphorylation. The molecular docking implied that **31** suppressed IL-1β production *via* binding to the TLR2/1 protein active site (Ding et al., 2019). Compounds **7**, **34**, and **82**–**84** revealed anti-inflammation potential (IC_50_s 19.4–34.4 μM) on NO formation induced by LPS in RAW264.7 macrophages, where **7** and **83** had potent potential (IC_50_s 22.6 and 19.9 μM, respectively) relative to PDTC (IC_50_ 23.1 μM, ammonium pyrrolidine dithiocarbamate) (Chen et al., 2020) (Table S5).

Hao et al. (2022) purified compound **55**, a new tetracyclic derivative from mangrove-associated *Aspergillus* sp. GXNU-A9 EtOAc extract utilizing SiO_2_ CC and HPLC. This metabolite (IC_50_ 8.22 μM) displayed a noticeable NO production inhibition capacity in RAW 264.7 cells boosted by LPS compared to dexamethasone (IC_50_ 5.62 μM). In another study, He et al. (2022) investigated *Melastoma malabathricum subsp. normale* roots utilizing SiO_2_/RP-18/Sephadex LH-20 CC/HPLC, resulting in a new derivative, guanxidone B (**56**) together with **12**, **51**, and **55**. Their structures were elucidated by spectral and CD analyses. Compounds **55** and **56** possessed marked anti-inflammation efficacy (IC_50_s 6.46 to 9.82 µM, respectively) *via* suppressing NO production utilizing Griess Reagent System compared to dexamethasone (IC_50_ 2.52 µM). Compound **56** was structurally similar to **51** having C-4 CH_2_OH instead of C-4 CH_3_ in **51**. It is noteworthy that **56** had better activity than **55**, indicating that CH_3_O at C-4 affected the activity (He et al., 2022).

Lobaric acid (**136**) separated from *Stereocaulon paschale* nordic lichen was found to prohibit TNF-α and IL-1β secretion and NF-κB activation boosted by LPS in macrophages. Docking results revealed its binding to PPAR-γ between beta-sheet and helix H3 as a partial PPAR-γ agonist, suggesting its efficacy because of NF-κB pathway blockage *via* PPAR-γ activation (Carpentier et al., 2018). These findings supported the development of **136** as PPAR-γ agonists for chronic inflammation disorders. From *Usnea subfloridana*, salazinic acid (**65**), galbinic acid (**49**), lobaric acid (**136**), conprotocetraric acid (**173**), and constictic acid (**174**) exerted antigout and antiinflammation capacities through inhibition of 5-LOX, COX1, XO, and COX2 in enzyme inhibition assays. It is noteworthy that **136** and **173** had effective COX2 inhibition capacity (IC_50_s 7.01 and 7.17 µM, respectively), compared to indomethacin (7.3 µM), whereas all of them exhibited potent XO inhibition activity (Nguyen et al., 2021).

### Anti-*Helicobacter pylori* activity

The inhibition of *Helicobacter pylori* urease activity is an effective strategy for treating this infectious disease. From *Cladonia rappii* acetone extract, **44** was separated by crystallization ((CH_3_)_2_CO:CHCl_3_ 20:1) and identified by spectral data. This compound was a marked competitive inhibitor of jack bean urease uricolytic activity. Also, it had a potent (MICs 0.034 to 0.068 μM) growth inhibition effectiveness against six clinical isolates of *H. pylori* than omeprazole (MICs 0.046–0.093 μM) in the broth microdilution assay. Therefore, **44** could be further developed for treating *H. pylori*-linked infections (Lage et al., 2018).

### Antioxidant activity

Methylstictic acid (**61**) having β-orcinol core with γ-lactone connected to B ring and aldehyde group at C-3 was separated using SiO_2_ CC and HPLC for the first time from *Hypotrachyna caraccensis*. It had reactivity and potency as DPPH^•^ scavenger as indicated by a kinetic study (EC_50_ 2.66 µM) compared to BHT (EC_50_ 0.11 µM) and ascorbic acid (EC_50_ 0.24 µM). It had optimal lipophilicity and permeability for penetrating the skin that could be utilized as a topical component for preventing oxidative injuries (Leal et al., 2018). The finding of antioxidant testing in the DPPH assay of mollicellins **96**–**98** and **101**–**104** showed that only **101** exhibited weak activity (IC_50_ 71.92 µg/mL) compared to BHT (IC_50_ 0.15 µg/mL) (Ouyang et al., 2018). Also, **134** possessed (IC_50_ 160 µg/mL) 5-times less antioxidant potential than resveratrol (IC_50_ 31.0 µg/mL) in the CUPRAC (CUPric-reducing-antioxidant capacity) assay (Studzińska-Sroka et al., 2021).

**Scheme 3:**
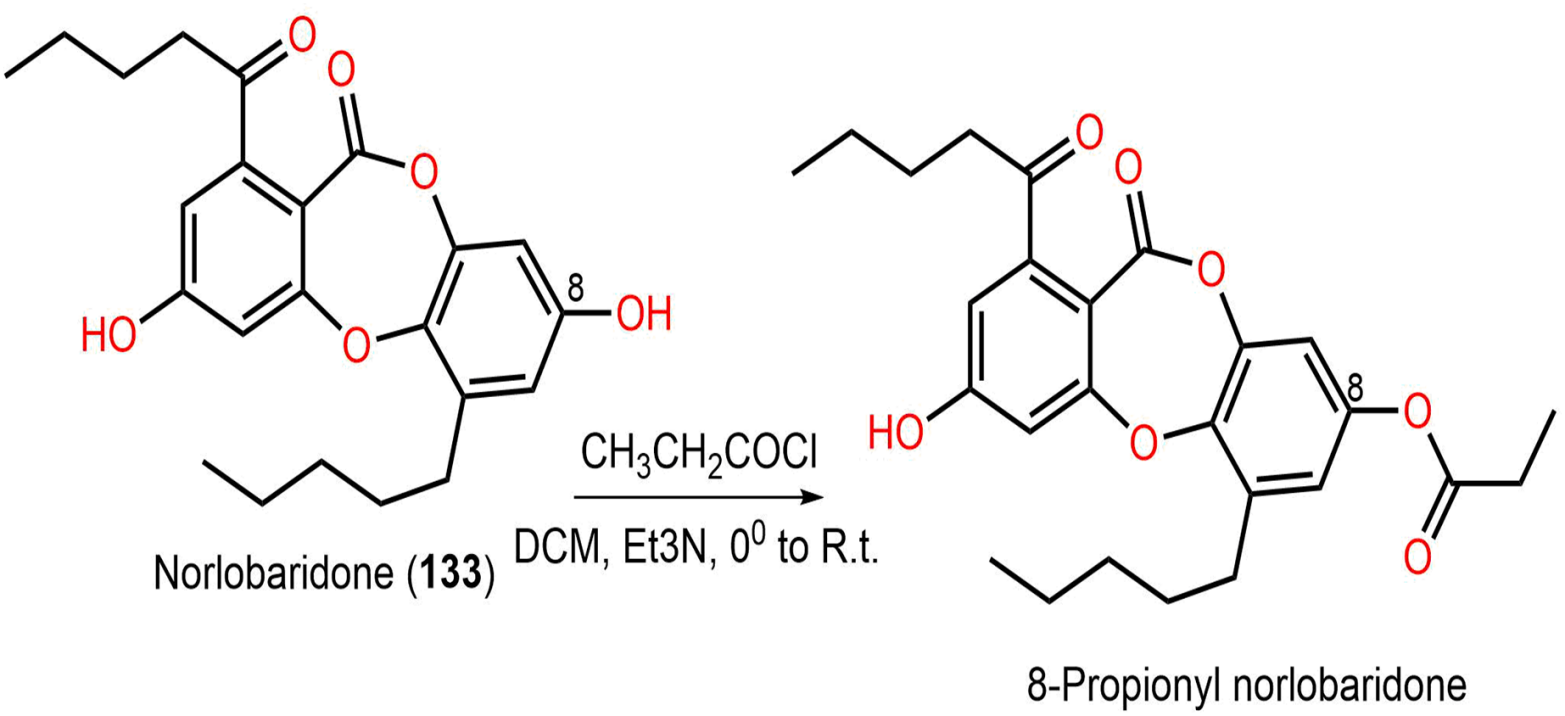
Semi-synthesis of norlobaridone (133) derivative (Pavan Kumar et al., 2020).

*Ramalina* lichenized fungi depsidones; **9**–**11**, **17**, **50**, **57**, **59**, **60**, **65**, **72**, **73**, and **134** were examined for antioxidant properties utilizing kinetic and thermodynamic calculations in the gaseous phase and aqueous solution. It was found that their BDE (bond-dissociation-energy) values were 74.4–87.7 kcal/mol, whereas **65**, **72**, and **73** had the lowest BDE(C-H)s (76.9, 74.4, and 75.2 kcal/mol^−1^, respectively). These metabolites were significant O_2_•− and HO• radical scavengers in aqueous media. Thus, depsidones exhibited potential O_2_•− and HO• radical scavenging capacity (Bay et al., 2020). In a study by Pavan Kumar et al. (2020), **133** isolated from *Parmotrema tinctorum* by SiO_2_ CC, along with its semi-synthesized derivative that was prepared using propionyl chloride were assessed for antioxidant potential in ABTS assay (Scheme 3). It was observed that **133** demonstrated potent effectiveness (%ABTS inhibition 98.90%, SC_50_ 20 μg/mL) compared to trolox (%ABTS inhibition 99.78%), while its derivative was inactive (%ABTS inhibition 4.7%, SC_50_ 20 μg/mL), revealing the importance of the free 8-OH group for the activity (Pavan Kumar et al., 2020).

### Antidiabetic activity

Diabetes mellitus is a worldwide rapidly disseminated metabolic disorder that is distinguished by persistent hyperglycemia because of the flaw in insulin action, insulin secretion, or both (Devi et al., 2020). Unrestrained hyperglycemia promotes protein glycation product formation (advanced-glycation-end products, AGEs). AGEs immoderate accumulation in diabetics enhances diabetic complication pathogenesis, including nephropathy, retinopathy, cardiomyopathy, and neuropathy. It was estimated that the number of diabetic patients has been rose from 108 million in 1980 to 422 million in 2014 (Zheng, Ley & Hu, 2018). In 2015, five million deaths were reported due to diabetes and its related complications, making it 9th causal factor of diminished life expectancy (Abubakar, Tillmann & Banerjee, 2015). In 2019, two million deaths were recorded caused by diabetes, and kidney diseases resulted from diabetes (World Health Organization, 2021). The available oral synthetic antidiabetics *e.g*., thiazolidinediones, biguanides, meglitinides, and sulfonylureas were reported to produce unwanted effects (Lorenzati et al., 2010). Thus, searching for new targets and approaches for treating diabetes is extremely recommended. α-Glucosidase (AG) is one of the fundamental enzymes implicated in carbohydrate digestion. It has been proven as an efficient target for diabetes management. However, the usage of the available alpha-glucosidase inhibitors (AGIs) such as miglitol, voglibose, 1-deoxynojirimycin, and acarbose has frequently been accompanied by side effects, in addition to the high costs. Many studies were carried out for identifying and validating the potential of natural products as AGIs for the prevention or curing of diabetes (Assefa et al., 2019).

The new derivatives, **54**, **67**, and **78**, along with **49** and **65** purified from *Parmotrema dilatatum* whole thalli acetone extract using SiO_2_ CC were assayed for their AG inhibition activities. Compounds **54**, **65**, and **78** revealed a notable AG inhibition (IC_50_s 2.2, 34.8, and 4.3 μM, respectively) while **49** and **67** were inactive compared with acarbose (IC_50_ 449 μM) (Devi et al., 2020). Structure-activity relation demonstrated the 3`-benzyl and C-3 aldehyde moieties enhanced the activity, while methylation of 8′-OH resulted in losing activity and γ-butyrolactone moiety did not influence the efficacy (Devi et al., 2020). Additionally, **53, 76**, **77**, **79**, and **80** new members of the depsidone family were separated from *Parmotrema tsavoense* utilizing SiO_2_ CC/TLC and assigned by spectral methods. Compound **80** is a 2*H*-chromene containing depsidone. Investigating AGI potential of **53**, **76**, and **77** revealed their marked inhibitory effectiveness (IC_50_s 11.4, 17.6, and 10.7 µM, respectively) than acarbose (IC_50_ 449 µM) in the colorimetric assay (Duong et al., 2020). Further investigation of *P. tsavoense* by Nguyen et al. (2022a) led to the separation of a new metabolite, **81** that demonstrated a powerful (IC_50_ 3.12 μM) AGI capacity than acarbose (IC_50_ 162.54 μM). Co-culturing of *Trichoderma* sp. 307 derived from *Clerodendrum inerme* with *Acinetobacter johnsonii* B2A (pathogenic aquatic bacteria) produced a new depsidone, botryorhodine H (**88**) and known analogs, **23**–**25** that were separated and characterized using Sephadex LH-20 and SiO_2_ CC and spectral analyses, respectively. These metabolites possessed powerful AG inhibition capacity (IC_50_s 8.1, 11.2, and 10.3 μM, respectively) than acarbose (IC_50_ 703.8 μM), whereas **25** displayed 13-fold more inhibition capacity (IC_50_ 54.1 μM) than acarbose (Table S6). It was indicated that C-3 functional groups influenced AGI activity (**88** *vs* **24** *vs* **25**), while the C-3′ CH_3_ group did not affect the activity (**23** *vs* **24**) (Zhang et al., 2018).

### Antihypertensive activity

RhoA (Ras homolog-gene family-member-A) is a member of the Rho—GTPase superfamily that was originally found to promote migration and cell cycle progress in cancer cells and control actin dynamics that are substantial for preserving the cell’s cytoarchitecture. It had been reported to have a marked role in cardiomyopathies and cardiac remodeling (Kilian et al., 2021). Also, the inhibition of RhoA activation reduced the angiotensin II-dependent hypertension development (Olivon et al., 2018). Olivon et al. identified the new metabolite, baillonic acid (**20**) along with **59** from New Caledonian *Meiogyne baillonii* bark EtOAc extract. Only **59** exhibited a significant RhoA-p115 complex GDP/GTP exchange inhibition potential (IC_50_ 187 μM, 50.5% inhibition) in the Biacore assay, thus it could have a potential for treating high blood pressure (Olivon et al., 2018).

### Anti-diarrheal activity

CFTR (cystic fibrosis transmembrane conductance regulator) is a cAMP-activated chloride channel that is accountable for the trans-epithelial secretion of chloride, resulting in the promoting force for intestinal fluid secretion (Li & Naren, 2010). The CFTR’s excessive function leads to secretory diarrhea, therefore its prohibition minimized intestinal fluid secretion. The CFTR inhibitory potential of **138**, **139**, **141**, **146**, **147**, **150**, **157**, **160**, and **164** in T84 cell monolayers using short-circuit current analysis was estimated. It is noteworthy that **138**, **139**, **141, 147, 150**, **157**, and **160** had remarkable (concentration 10 μM, >50% inhibition) CFTR-mediated chloride secretion inhibition where **160** and **157** were the most powerful. Compounds **157** and **160** dose-dependently prohibited forskolin-boosted chloride secretion in T84 cells (IC_50_s 0.5 and 2.0 μM, respectively) with almost complete suppression at concentrations of 20 and 10 μM, respectively, whereas **157** was more potent than **160**. Further investigation of **157** for their effect on CT (cholera toxin)-boosted chloride secretion across T84 cells. CT is an enterotoxin accountable for massive symptoms of cholera patients’ diarrhea. Compound **157** was found to dose-dependently prohibit CT-induced chloride secretion (IC_50_ 5.0 μM) with complete prohibition at a concentration of 100 μM. These findings revealed an anti-secretory potential of **157** and **160** that could be beneficial for diarrhea treatment (Phainuphong et al., 2018).

### BChE (butyrylcholinesterase) and AChE (acetylcholinesterase), and phosphodiesterase inhibition activities

Neurodegenerative illnesses, such as Alzheimer’s (AD) or Parkinson’s disease (PD) represent a critical global health concern. They are a series of procedures that result in the gradual forfeiture of neuronal function and nerve cell death (Di Paolo et al., 2019). BChE (butyrylcholinesterase) and AChE (acetylcholinesterase) are substantial for CNS functions that hydrolyze acetylcholine (Studzińska-Sroka et al., 2021). Acetylcholine hydrolysis suppression is substantial in neuro-degenerative illnesses. Moreover, BChE and AChE noncholinergic actions like the impact on cellular adhesion and proliferation process regulation are also crucial in brain tumors (Studzińska-Sroka et al., 2021).

Rukachaisirikul et al. (2019) stated that **23** and **27** possessed PDE5 (−5) inhibition capacity (% inhibition 84% and 89% and IC_50_s 5.69 and 9.96 μM, respectively). Studzińska-Sroka et al. investigated **134** AChE and BChE inhibition potentials using Ellman’s colorimetric method. It only prohibited BChE (%inhibition 8.1%) (Studzińska-Sroka et al., 2021). Compound **148** reported from *A. unguis* displayed AChE inhibition potential (IC_50_ 102.4 µM), while **138**, **139**, **146**, **147**, **150,** and **153** had weak or no effectiveness (Yang et al., 2018).

### Tyrosinase and hyaluronidase inhibitory activities

Hyaluronic acid (HA) is a brain-extracellular matrix prime component that is generated by Hyaluronan synthase (HAS) and broken down into fragments by hyaluronidase (Misra et al., 2011). The resulting fragments were reported to be related to enhanced cancer cell invasion capability and proliferation, as well as proangiogenic and proinflammation processes (Chen et al., 2018b).

Studzińska-Sroka et al. (2021) stated that **134** had a high hyaluronidase suppression potential with IC_50_ 0.053 mg/mL that was 6–10 times more powerful than tannic acid (IC_50_ 0.554 mg/mL).

Tyrosinase oxidizes surplus dopamine to form dopamine quinones, quite reactive species that promote cell death and neural damage. It is implicated in neurodegeneration-related illnesses like Parkinson’s disease (Chen et al., 2018b). Thus, its prohibition is targeted to discover new drugs for these disorders, particularly Parkinson’s disease. Compound **134** showed 25% inhibition of tyrosinase enzyme (Conc. 1.6 mg/mL), which was 3-times lower than azelaic acid using L-DOPA (substrate) (Studzińska-Sroka et al., 2021).

### Anti-osteoclastogenic activity

Bone homeostasis is maintained and regulated by two metabolic processes, bone formation by osteoblasts and bone resorption by osteoclasts (Jacome-Galarza et al., 2019). Osteoclast differentiation is controlled by two factors, the M-CSF (macrophage colony stimulation factor) and RANKL (receptor activator of the nuclear factor kappa-B ligand). Signaling pathways of RANKL are considered key targets for prohibiting bone resorption and osteoclast differentiation (Tan et al., 2020). NF-κB has a pivotal function in RANKL-caused osteoclast differentiation (Zhang et al., 2022).

Zhang et al. (2022) investigated the inhibitory potential of **147**, **149**, 150, and **154** on RANKL-induced osteo-clastogenesis in RAW264.7 macrophages and BMMs (bone marrow macrophage cells) using luciferase reporter gene and TRAP (tartate-resisant acid phosphatase) assays, respectively. It was found that **147**, **149**, and **154** demonstrated prohibition of LPS-caused NF-κB activation in RAW264.7 macrophages (Conc. 20 μM).

### Phytotoxic activity

Norcolensoic acid (**132**) was found to prohibit lettuce seedlings’ root growth (% inhibition 92 and 63%, respectively at Conc. 300 and 100 μg/mL, respectively), whereas it strongly suppressed seed germination at a concentration of 500 μg/mL (Shiono, Koseki & Koyama, 2018).

### Antiviral activity

*Cordia millenii* investigation resulted in a new analog, **6** that was assigned by spectral and X-ray analyses. This compound had promising HIV-1 integrase efficacy (IC_50_ 4.65 µM) in comparison to chicoric acid (IC_50_ 0.33 µM) (Dongmo Zeukang et al., 2019).

Salazinic (**65**) and protocetraric (**17**) acids were reported as 3CLpro SARS-CoV-2 slow-binding inactivators (*Ki* of 3.77 and 3.95 µM, respectively) that could be possible scaffolds for the development of effective SARS-CoV-2 cysteine enzyme 3CLpro inhibitors (Fagnani et al., 2022).

### GnRH (Gonadotropin-releasing hormone) antagonistic activity

Nguyen et al. (2022b) proved that the treatment of female rats with lobaric acid (**136**) (50 and 100 mg/kg b.w./day) significantly ameliorated tetramethrin (50 mg/kg b.w/day)-induced alteration on estrous cycle. It reversed gonadotropins serum levels through influencing the pituitary/hypothalamic axis and competitively inhibited tetramethrin binding to GnRH receptor in both thermodynamic and kinetic processes.

## Semisynthetic depsidone derivatives

**Figure 14:**
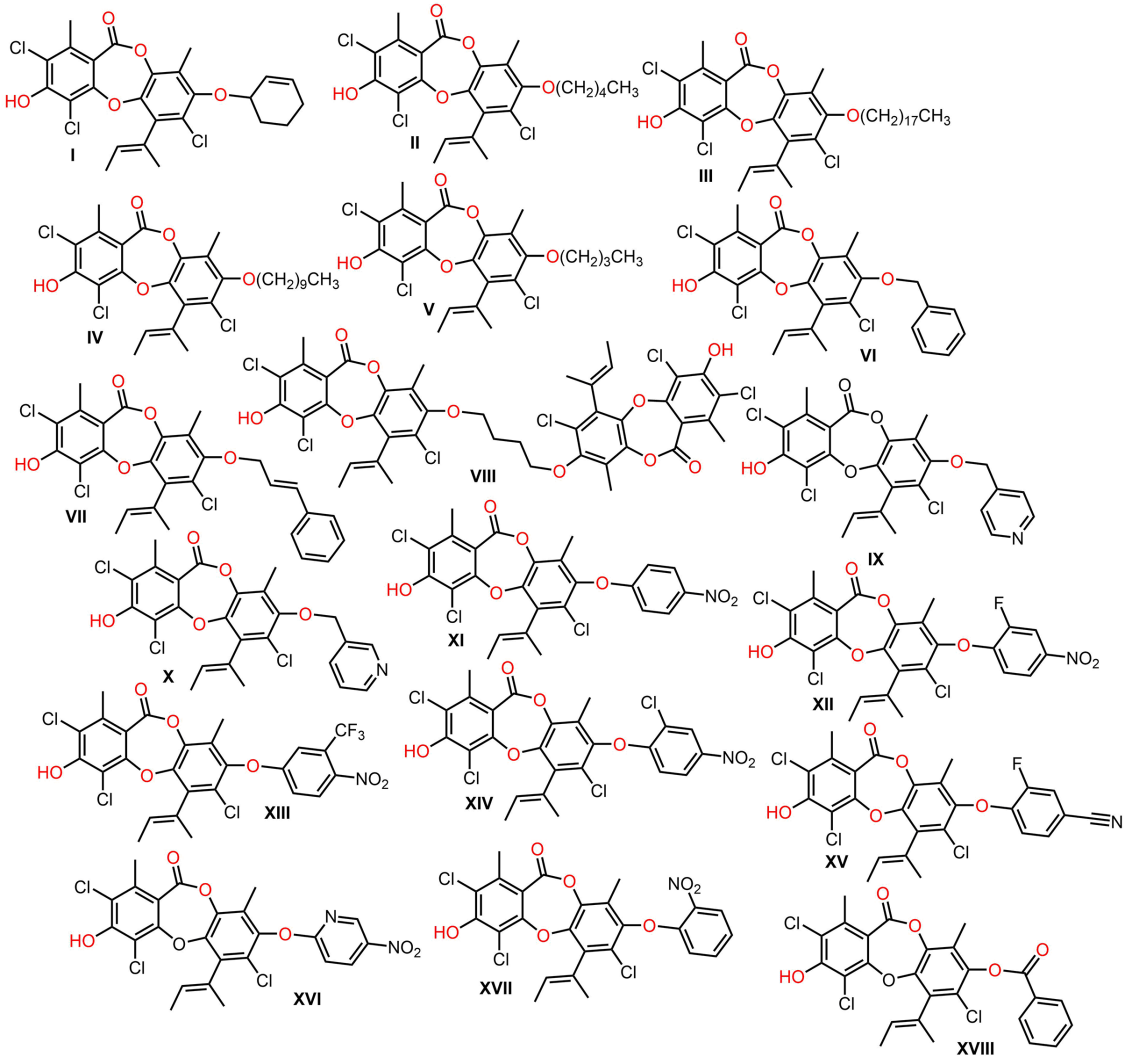
Semisynthetic derivatives of nornidulin (I–XVIII).

Isaka et al. (2019) synthesized derivatives of **146** utilizing regioselective arylation, acylation, and alkylation reactions to give 8-O-substituted analogs that were assessed for their antibacterial potential. Many of the 8-O-alkyl derivatives had more powerful antibacterial capacities than **146**, whereas 8-O**-**butyl exhibited the highest potential against *B. cereus* (MIC 0.391 μg/mL), however, the derivatives with long sidechains, as well as acylated derivatives displayed weaker capacity. On the other hand, O-aryl analogs demonstrated powerful antibacterial potential against MRSA (Isaka et al., 2019) (Fig. 14).

**Scheme 4:**
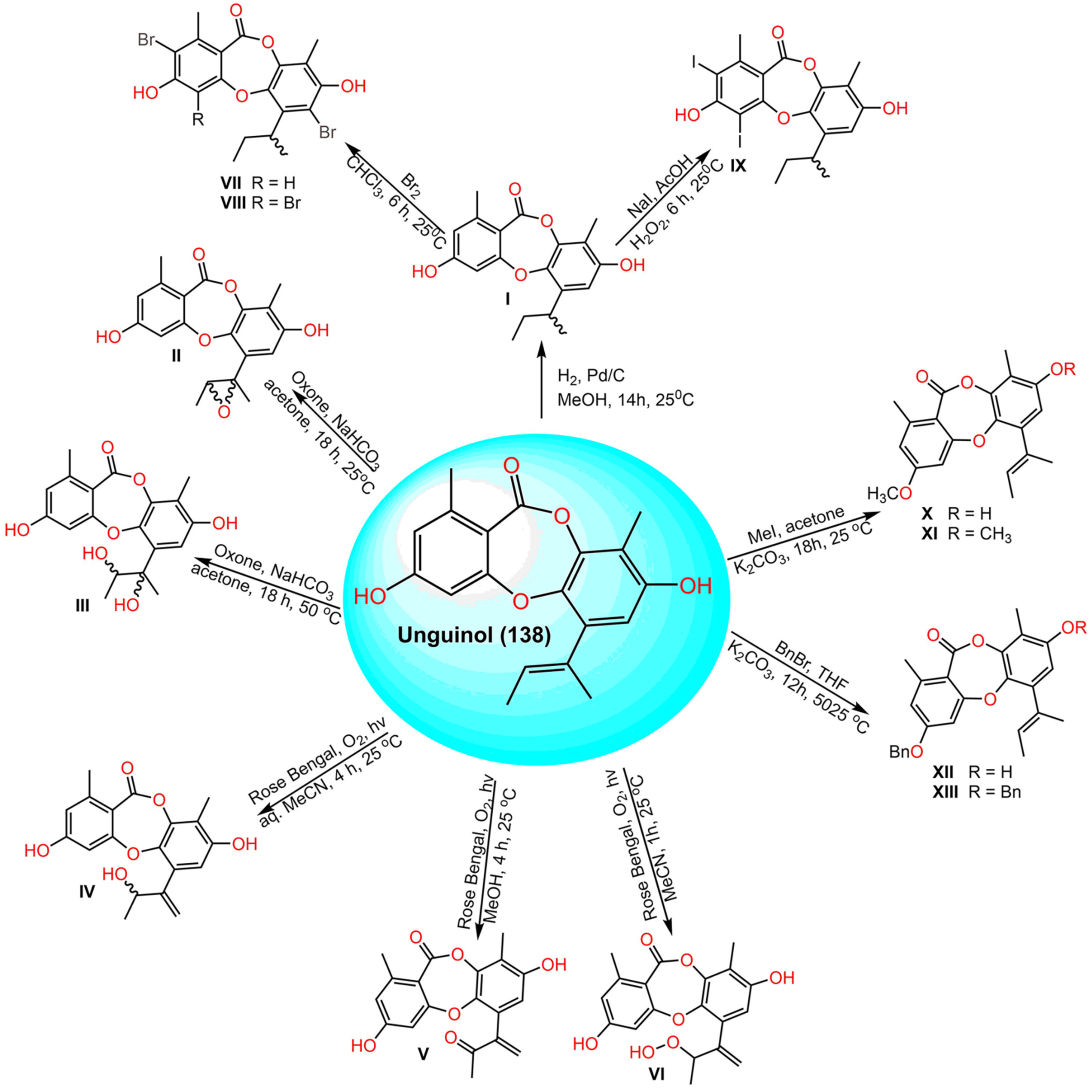
Semisynthetic derivatives (I–XIII) of unguinol (138) (Morshed et al., 2021).

Morshed et al. (2021) prepared semisynthetic derivatives of **138** (Scheme 4). Among them, 3-O-(2,4-difluorobenzyl)unguinol and 3-O-(2-fluorobenzyl)unguinol possessed remarkable antibacterial effectiveness against methicillin-susceptible and -resistant *S. aureus* (MIC 0.25–1 µg/mL).

## Conclusion

Natural metabolites biosynthesized by various living organisms are renowned for their vital contribution to drug design and discovery. In this work, a total of 172 depsidones were reported from various sources from 2018 to 2022 with a greater number separated in 2022. The major depsidone derivatives are reported from fungi (107 compounds, 62.2%), then lichens (52 compounds, 30.3%), and the least number of depsidones were reported from plant sources (13 compounds, 7.5%). These metabolites were commonly separated from the species belonging to the following genera: *Aspergillus*, *Chaetomium*, and *Spiromastix* (fungi); *Usnea, Parmotrema*, and *Ramalina* (lichens), and *Melastoma, Hypericum*, and *Garcinia* (plants).

It is noteworthy that these metabolites show various structural features according to their sources. It was noted that fungal-derived depsidones possess various substituents such as pyran (*e.g*., **83**, **86**, **91**, **99**, **100**, and **102**–**104**), substituted benzyl (*e.g*., **88**–**90**), isoprenyl (*e.g*., **92**–**94**, **96**–**98**, and **105**–**111**), 2-methylbut-2-enyl (*e.g*., **138**–**167**), three to five carbon aliphatic chain (*e.g*., **115**–**129**), and halogen (bromine (*e.g*., **121**, **123**, **125**, **127**, **129**, **144**, **145**, **151**, **152**, and **159**) and chlorine (*e.g*., **116**–**120**, **139**–**144**, **154**–**156**, and **160**–**163**)) and lichen-derived depsidones feature furan (*e.g*., **45**–**52** and **57**–**73**), substituted benzyl (*e.g*., **54** and **74**–**79**), and five to seven aliphatic chains (*e.g*., **130**–**137**), while that reported from the plant have isoprenyl substituent (*e.g*., **168**–**170**).

These metabolites were evaluated for various bioactivities mainly antimicrobial, cytotoxic, and antidiabetic capacities. Depsidones could have the potential as lead metabolites for neurodegenerative illnesses and diabetes through their inhibition of butyrylcholinesterase, tyrosinase, α-glucosidase, and acetylcholinesterase enzymes. Besides, **127** could be a potential lead for bactericides to control rice bacterial-blight disease. Also, **44** demonstrated powerful anti-*H. pylori* potential that could be further developed for treating *H. pylori*-linked infections. It was found that the ring substation patterns greatly influenced the activities as highlighted in some reports on structure-activity relation (Shukla et al., 2019; Niu et al., 2021b).

Preparation of semi-synthetic derivatives from these compounds resulted in derivatives with more powerful activity than parent compounds *e.g*., unguinol and nidulin, which could encourage medicinal chemists to carry out further modification of the structures of other reported metabolites and assess the effect of this modification on the bioactivities. Besides, altering cultural media conditions could be an efficient strategy to get novel biometabolites. Also, the co-culturing of two or more organisms from different species produced interesting metabolites that have not been produced in the cultivation of the organism alone. Therefore, this approach could be further utilized for discovering more valuable metabolites. Collectively, depsidone derivatives feature diversified chemical entities and numerous bioactivities. These metabolites could be beneficial scaffolds and building blocks for synthesizing various drugs for multiple human health disorders. However, the *in vivo* evaluation of their potential biological properties and mechanistic investigations should indubitably be the focal point of future studies.

## Supplemental Information

10.7717/peerj.15394/supp-1 Supplemental Information 1 Supplemental Tables.

## List of abbreviations

A-172: Glioblastoma cell line
A549: Human lung adenocarcinoma epithelial cell line
ABTS: 2,2`-Azinobis-(3-ethylbenzthiazoline-6-sulphonate)
ACHN: Human renal carcinoma cell lines
AChE: Acetylcholinesterase
Bel-7402: Human hepatocellular carcinoma cell line
BHT: Butylated hydroxytoluene
CCK-8: Cell counting kit-8
CD: Circular dichroism
CFTR: Cystic fibrosis transmembrane conductance regulator
CH_2_Cl_2_: Dichloromethane
COX1: Cyclooxygenase-1
COX2: Cyclooxygenase-2
DPPH: 1,1-Diphenyl-2-picrylhydrazyl
EC_50_: Half maximal effective concentration
ECD: Electronic circular dichroism
EtOH: Ethanol
EtOAc: Ethyl acetate
ESI-MS: Electrospray ionization mass spectrometry
GFPMA: Green fluorescent protein microplate assay
HCT-15: Human colon cancer cell line
HCT-116: Human colon cancer cell line
HeLa: Human cervical epitheloid carcinoma cell line
HepG2: Human hepatocellular liver carcinoma cell line
HPLC: High-performance liquid chromatography
HRESIMS: High resolution electrospray ionization mass spectroscopy
HT-29: Human colon cancer cell line
IC_50_: Half-maximal inhibitory concentration
IL-1β: Interleukin*-*1β
KB: Human oral epidermoid carcinoma cell line
LC_50_: Lethal concentration that kills 50%
IR: Infrared
LPS: Lipopolysaccharide
5-LOX: 5-Lipooxygenase
MCF-7: Human breast adenocarcinoma cell line
MDR: Multidrug-resistant
MDA-MB-231: Human breast cancer cell line
Med25: Mediator of RNA polymerase II transcription subunit 25
MeOH: Methanol
MIC: Minimum inhibitory concentration
MRSA: Methicillin-resistant *Staphylococcus aureus*
MS: Mass spectrometry
MTS: (3-(4,5-Dimethylthiazol-2-yl)-5-(3-carboxymethoxyphenyl)-2-(4-sulfophenyl)-2H-tetrazoliuminner salt)
MTT: 3-(4,5-Dimethylthiazol-2-yl)-2,5-diphenyltetrazolium bromide
TM4 cells: Murine Sertoli cells
NCI-H460: Human non-small cell lung cancer cell line
NCI-H187: Human small-cell lung cancer
NCI-H23: Human lung cancer cell line
NF-κB: Nuclear factor kappa-light-chain-enhancer of activated B cells
NMR: Nuclear magnetic resonance
NUGC-3: Human stomach cancer cell line
NO: Nitric oxide
PC-3: Human prostatic-testosterone-independent cell line
PDE5: Phosphodiesterase
ROS: Reactive oxygen species
PPAR-γ: Peroxisome proliferator—activated receptor gamma
RP-18: Reversed phase-18
SC_50_: Scavenging concentration 50%
SiO_2_ CC: Silica gel column chromatography
PPIs: Protein-protein interactions
T98G: Glioblastoma cell line
TLC: Thin layer chromatography
TNF-α: Tumor necrosis factor alpha
TRAIL: Tumor necrosis factor-related apoptosis-inducing ligand
U-138MG: Glioblastoma cell line
U937: Pro-monocytic, human myeloid leukemia cell line
Vero cell: Normal African green monkey kidney fibroblasts
XO: xanthine oxidase
XRMA: 2,3-bis-(2-methoxy-4-nitro-5-sulfophenyl)-2H-tetrazolium-5-carboxanilide (XTT) reduction menadione assay.

## Additional Information and Declarations

### Data Availability

The following information was supplied regarding data availability: This is a literature review.
