# Supplementary material for: Recent advances on natural depsidones: sources, biosynthesis, structure-activity relationship, and bioactivities
Source: PeerJ. 2023 May 12;11:e15394. doi: 10.7717/peerj.15394 (PMC10184659; doi:10.7717/peerj.15394)
Supplement: Supplemental Information 1 [file peerj-11-15394-s001.docx]

**Supplementary Materials**

**Recent advances on natural depsidones: Sources, biosynthesis, structure-activity relationship, and bioactivities**

Maan T. Khayat^1,^, Kholoud F. Ghazawi^2^, Waad A. Samman^3^, Aisha A. Alhaddad^3^, Gamal A. Mohamed,^4^ Sabrin R. M. Ibrahim^5,6,*^

^1^ Department of Pharmaceutical Chemistry, Faculty of Pharmacy, King Abdulaziz University, Jeddah 21589, Saudi Arabia; Mkhayat@kau.edu.sa

^2^ Clinical Pharmacy Department, College of Pharmacy, Umm Al-Qura University, Makkah 24382, Saudi Arabia; [kfghazawi@uqu.edu.sa](mailto:kfghazawi@uqu.edu.sa)

^3^ Department of Pharmacology and Toxicology, College of Pharmacy, Taibah University, Al-Madinah Al-Munawwarah 30078, Saudi Arabia; [aahaddad@taibahu.edu.sa](mailto:aahaddad@taibahu.edu.sa) (A.A.A.); [WSAMMAN@taibahu.edu.sa](mailto:WSAMMAN@taibahu.edu.sa) (W.A.S.)

^4^ Department of Natural Products and Alternative Medicine, Faculty of Pharmacy,
King Abdulaziz University, Jeddah 21589, Saudi Arabia; [gahussein@kau.edu.sa](mailto:gahussein@kau.edu.sa)

^5^ Preparatory Year Program, Department of Chemistry, Batterjee Medical College, Jeddah 21442, Saudi Arabia

^6^ Department of Pharmacognosy, Faculty of Pharmacy, Assiut University, Assiut 71526, Egypt; sabreen.ibrahim@pharm.aun.edu.eg

Corresponding Author:

Sabrin R. M. Ibrahim^1,2^

Preparatory Year Program, Department of Chemistry, Batterjee Medical College, Jeddah 21442, Saudi Arabia

Email address: sabrin.ibrahim@bmc.edu.sa; Tel.: +966-581183034

**Table S1:** Naturally occurring depsidone and their derivatives (Source, host, place, molecular weights, and formulae).

| **Compound name** | **M.Wt.** | | **M. F.** | **Source** | Host (Part) | | **Source, Place** | | **Ref.** |
| --- | --- | --- | --- | --- | --- | --- | --- | --- | --- |
| Polyanthadepsidone A (**1**) | 346 | | C_18_H_18_O_7_ | *Garcinia polyantha*  (Plant, leaves) | *-* | | Yaoundé, Center region, Cameroon | | (Lannang et al., 2018) |
| Cetraric acid (**2**) | 402 | | C_20_H_18_O_9_ | *Usnea longissima*  (Beard lichen) | - | | Sichuan, Yunnan, Zhejiang, and Shanxi, China | | (Jin et al., 2018) |
| Psoromic acid (**3**) | 358 | | C_18_H_14_O_8_ | *Usnea longissima*  (Beard lichen) | - | | Sichuan, Yunnan, Zhejiang, and Shanxi, China | | (Jin et al., 2018) |
|  | - | | - | *Homalium cochinchinensis* (Plant, aerial parts) | *-* | | Cotuy forest, Nui Chua National Park, Ninh Thuan, Ninh Hai District, Vietnam | | (Addo et al., 2021) |
| Methyl psoromate (**4**) | 372 | | C_19_H_16_O_8_ | *Usnea longissima*  (Beard lichen) | - | | Sichuan, Yunnan, Zhejiang, and Shanxi, China | | (Jin et al., 2018) |
| Virensic acid (**5**) | 358 | | C_18_H_14_O_8_ | *Usnea longissima*  (Beard lichen) | - | | Sichuan, Yunnan, Zhejiang, and Shanxi, China | | (Jin et al., 2018) |
| Cordidepsine (**6**) | 330 | | C_17_H_4_O_7_ | *Cordia millenii*  (Plant, stem bark) | - | | Batoufam, West region of Cameroon | | (Dongmo Zeukang et al., 2019) |
| Corynesidone A (**7**) | 272 | | C_15_H_12_O_5_ | *Boeremia exigua* (Fungus) | *Solanum tuberosum* (Potato) | | Lincang, Yunnan, China | | (Chen et al., 2020) |
| Corynesidone D (**8**) | 316 | | C_16_H_12_O_7_ | *Aspergillus unguis* BCC54176 (Fungus) | *Coriandrum sativum* (Leaf) | | Ying Charoen market, north Bangkok, Thailand | | (Sadorn et al., 2022) |
| Hypoprotocetraric acid (**9**) | 344 | | C_18_H_16_O_7_ | Genus *Ramalina* (Lichen) | - | | - | | (Bay et al., 2020) |
| Conhypoprotocetraric acid (**10**) | 360 | | C_18_H_16_O_8_ | Genus *Ramalina* (Lichen) | - | | - | | (Bay et al., 2020) |
| Gangaleoidin (**11**) | 412 | | C_18_H_14_Cl_2_O_7_ | Genus *Ramalina* (Lichen) | - | | - | | (Bay et al., 2020) |
| Dioxepin-11-one (**12**) | 300 | | C_17_H_16_O_5_ | *Melastoma malabathricum* (Plant, Roots) | - | | Yanshan Town Guilin, China | | (He et al., 2022) |
| Parmosidone A (**13**) | 374 | | C_18_H_14_O_9_ | *Parmotrema tsavoense* (Lichen) | - | | Rocks on Ta Cu Mountain, Ham Thuan Nam district, Binh Thuan, Vietnam | | (Duong et al., 2015) |
| Parmosidone B (**14**) | 362 | | C_17_H_14_O_9_ | *Parmotrema tsavoense* (Lichen) | - | | Rocks on Ta Cu Mountain, Ham Thuan Nam district, Binh Thuan, Vietnam | | (Duong et al., 2015) |
| Parmosidone E (**15**) | 358 | | C_18_H_14_O_8_ | *Parmotrema tsavoense* (Lichen) | - | | Rocks on Ta Cu Mountain, Ham Thuan Nam district, Binh Thuan, Vietnam | | (Duong et al., 2015) |
| Vicanicin (**16**) | 382 | | C_18_H_16_Cl_2_O_5_ | *Teloschistes flavicans* (Lichen) | *-* | | Rejang Lebong, Bengkulu, Indonesia | | (Sanjaya et al., 2020) |
| Protocetraric acid (**17**) | 374 | | C_18_H_14_O_9_ | *Usnea baileyi* (Lichen) | - | | Lam Dong, Vietnam | | (Van Nguyen et al., 2018) |
| 1H-Dibenzo[b,e][1,4]dioxepin-11-one,3,8-dihydroxy-4-(methoxymethyl)-1,6-dimethyl (**18**) | 302 | | C_16_H_14_O_6_ | *Lasiodiplodia theobromae* M4.2-2 (Fungus) | Mangrove sediment | | Dongzhai Harbor, Hainan, China | | (Umeokoli et al., 2019) |
| Flavicansone (**19**) | 382 | | C_18_H_16_O_5_Cl_2_ | *Teloschistes flavicans* (Lichen) | *-* | | Rejang Lebong, Bengkulu, Indonesia | | (Sanjaya et al., 2020) |
| Baillonic acid (**20**) | 374 | | C_18_H_14_O_9_ | *Meiogyne baillonii*  (Plant, Bark) | - | | Nodela Valle, New Caledonia | | (Olivon et al., 2018) |
| Botryorhodine A = Botryosphaerone A **(21)** | 300 | | C_16_H_11_O_6_ | *Lasiodiplodia theobromae* M4.2-2 (Fungus) | Mangrove sediment | | Dongzhai Harbor, Hainan, China | | (Umeokoli et al., 2019) |
|  | - | | - | *Simplicillium lanosoniveum* PSU-H168 and PSU-H261  (Fungus) | *Hevea brasiliensis* (Plant, Leaves) | | Songkhla, Thailand | | (Rukachaisirikul et al., 2019) |
|  | - | | - | *Lasiodiplodia pseudotheobromae* (Fungus) | Soil | | Hainan wetland park China | | (Liang et al., 2022) |
| Botryorhodine B = Botryosphaerone B (**22**) | 314 | | C_17_H_14_O_6_ | *Simplicillium lanosoniveum* PSU-H168 and PSU-H261  (Fungus) | *Hevea brasiliensis* (Plant, Leaves) | | Songkhla, Thailand | | (Rukachaisirikul et al., 2019) |
|  | - | | - | *Lasiodiplodia theobromae* M4.2-2 (Fungus) | Mangrove sediment | | Dongzhai Harbor, Hainan, China | | (Umeokoli et al., 2019) |
|  | - | | - | *Lasiodiplodia pseudotheobromae* (Fungus) | Soil | | Hainan wetland park China | | (Liang et al., 2022) |
| Botryorhodine C = Botryosphaerone C (**23**) | 416 | | C_17_H_16_O_6_ | *Trichoderma* sp. 307 co-culturing with *Acinetobacter johnsoni*i B2 (Fungus) | *Clerodendrum inerme* (Mangrove plant) | | Zhanjiang Mangrove National Nature Reserve, Guangdong, China | | (Zhang et al., 2018) |
| Botryorhodine D = Botryosphaerone D (**24**) | 302 | | C_16_H_14_O_6_ | *Trichoderma* sp. 307 co-culturing with *Acinetobacter johnsoni*i B2 (Fungus) | *Clerodendrum inerme* (Mangrove plant) | | Zhanjiang Mangrove National Nature Reserve, Guangdong, China | | (Zhang et al., 2018) |
|  | - | | - | *Lasiodiplodia theobromae* M4.2-2 (Fungus) | Mangrove sediment | | Dongzhai Harbor, Hainan, China | | (Umeokoli et al., 2019) |
| Botryorhodine G (**25**) | 316 | | C_17_H_16_O_6_ | *Trichoderma* sp. 307 co-culturing with *Acinetobacter johnsoni*i B2 (Fungus) | *Clerodendrum inerme* (Mangrove plant) | | Zhanjiang Mangrove National Nature Reserve, Guangdong, China | | (Zhang et al., 2018) |
|  | - | | - | *Lasiodiplodia pseudotheobromae* (Fungus) | Soil | | Hainan wetland park China | | (Liang et al., 2022) |
| Botryorhodine I (**26**) | 318 | | C_16_H_14_O_7_ | *Lasiodiplodia theobromae* M4.2-2 (Fungus) | Mangrove sediment | | Dongzhai Harbor, Hainan, China | | (Umeokoli et al., 2019) |
| Simplicildone A (**27**) | 330 | | C_18_H_18_O_6_ | *Simplicillium lanosoniveum* PSU-H168 and PSU-H261  (Fungus) | *Hevea brasiliensis* (Plant, Leaves) | | Songkhla, Thailand | | (Rukachaisirikul et al., 2019) |
|  | - | | - | *Lasiodiplodia theobromae* M4.2-2 (Fungus) | Mangrove sediment | | Dongzhai Harbor, Hainan, China | | (Umeokoli et al., 2019) |
| Simplicildone B (**28**) | 344 | | C_19_H_20_O_6_ | *Simplicillium lanosoniveum* PSU-H168 and PSU-H261  (Fungus) | *Hevea brasiliensis* (Plant, Leaves) | | Songkhla, Thailand | | (Rukachaisirikul et al., 2019) |
| Curdepsidone A (**29**) | 332 | | C_16_H_12_O_8_ | *Curvularia* sp. IFB-Z10 (Fungus) | *Genyonemus lineatus*  (Marine White croaker) | | Nanjing, Jiangsu, China | | (An et al., 2018) |
| Curdepsidone B (**30**) | 448 | | C_22_H_24_O_10_ | *Curvularia* sp. IFB-Z10 (Fungus) | *Argyrosomus argentatus* (Croaker gut) | | Yellow Sea, Sea nearthe Lvsi Port, China | | (Ding et al., 2019) |
|  | - | | - | *Boeremia exigua* (Fungus) | *Solanum tuberosum* (Potato) | | Lincang, Yunnan, China | | (Chen et al., 2020) |
| Curdepsidone C (**31**) | 448 | | C_22_H_24_O_10_ | *Curvularia* sp. IFB-Z10 (Fungus) | *Argyrosomus argentatus* (Croaker gut) | | Yellow Sea, Sea nearthe Lvsi Port, China | | (Ding et al., 2019) |
|  | - | | - | *Boeremia exigua* (Fungus) | *Solanum tuberosum* (Potato) | | Lincang, Yunnan, China | | (Chen et al., 2020) |
| Curdepsidone D (**32**) | 462 | | C_23_H_26_O_10_ | *Curvularia* sp. IFB-Z10 (Fungus) | *Argyrosomus argentatus* (Croaker gut) | | Yellow Sea, Sea nearthe Lvsi Port, China | | (Ding et al., 2019) |
| Curdepsidone E (**33**) | 462 | | C_23_H_26_O_10_ | *Curvularia* sp. IFB-Z10 (Fungus) | *Argyrosomus argentatus* (Croaker gut) | | Yellow Sea, Sea nearthe Lvsi Port, China | | (Ding et al., 2019) |
| Curdepsidone F (**34**) | 302 | | C_16_H_14_O_6_ | *Curvularia* sp. IFB-Z10 (Fungus) | *Argyrosomus argentatus* (Croaker gut) | | Yellow Sea, Sea nearthe Lvsi Port, China | | (Ding et al., 2019) |
|  | - | | - | *Boeremia exigua* (Fungus) | *Solanum tuberosum* (Potato) | | Lincang, Yunnan, China | | (Chen et al., 2020) |
| Curdepsidone G (**35**) | 346 | | C_18_H_18_O_7_ | *Curvularia* sp. IFB-Z10 (Fungus) | *Argyrosomus argentatus* (Croaker gut) | | Yellow Sea, Sea nearthe Lvsi Port, China | | (Ding et al., 2019) |
| 8’-*O*-methylprotocetraric acid (**36**) | 388 | | C_19_H_16_O_9_ | *Parmotrema dilatatum* (Lichen thalli) | - | | Rocks in Lam Dong, Vietnam | | (Duong T., 2019) |
| Livistone A (**37**) | 302 | | C_16_H_14_O_6_ | *Lasiodiplodia pseudotheobromae* (Fungus) | Soil | | Hainan wetland park China | | (Liang et al., 2022) |
| Livistone B (**38**) | 316 | | C_17_H_16_O_6_ | *Lasiodiplodia pseudotheobromae* (Fungus) | Soil | | Hainan wetland park China | | (Liang et al., 2022) |
| Lasiodiplodiaone A (**39**) | 374 | | C_20_H_22_O_7_ | *Lasiodiplodia pseudotheobromae* (Fungus) | Soil | | Hainan wetland park China | | (Liang et al., 2022) |
| Lasiodiplodiaone B (**40**) | 388 | | C_21_H_24_O_7_ | *Lasiodiplodia pseudotheobromae* (Fungus) | Soil | | Hainan wetland park China | | (Liang et al., 2022) |
| Asperunguissidone A (**41**) | 342 | | C_19_H_18_O_6_ | *Aspergillus unguis* PSU-MF16 (Fungus) | *Dysidea* (Sponge) | | Koh Bulon Mai Pai, Satun, Thailand | | (Saetang et al., 2021) |
| Asperunguissidone B (**42**) | 376 | | C_19_H_17_ClO_6_ | *Aspergillus unguis* PSU-MF16 (Fungus) | *Dysidea* (Sponge) | | Koh Bulon Mai Pai, Satun, Thailand | | (Saetang et al., 2021) |
| Physodalic acid (**43**) | 402 | | C_19_H_14_O_10_ | *Hypogymnia physodes* (Lichen_ | - | | Jastrz˛ebsko Stare, Poland | | (Studzińska-Sroka et al., 2021) |
| Fumarprotocetraric acid (**44**) | 472 | | C_22_H_16_O_12_ | *Cladonia rappii* (Lichen) | *-* | | Serra do Brigadeiro State Park, Araponga, Minas Gerais, Brazil | | (Lage et al., 2018) |
|  | - | | - | *Usnea longissima* (Beard lichen) | - | | Sichuan, Yunnan, Zhejiang, and Shanxi, China | | (Jin et al., 2018) |
|  | - | | - | *Lichen rangiferinus* (Lichen) | *-* | | Uttar Pradesh, India | | (Shukla et al., 2019) |
|  | - | | - | *Cladonia pyxidate* (Lichen) | Rocks | | Gangotri (Uttarkashi district), Uttarakhand, India | | (Thuan et al 2022) |
| Fumarprotocetraric acid lactone 1 (**45**) | 502 | | C_22_H_14_O_14_ | *Cladonia metacorallifera* (Antarctic lichen) | - | | Peninsula Fildem, King George Island, Antarctica | | (Sepúlveda et al., 2022) |
| Fumarprotocetraric acid lactone 2 (**46**) | 486 | | C_22_H_14_O_13_ | *Cladonia metacorallifera* (Antarctic lichen) | - | | Peninsula Fildem, King George Island, Antarctica | | (Sepúlveda et al., 2022) |
| Bailesidone (**47**) | 414 | | C_21_H_18_O_9_ | *Usnea baileyi* (Lichen) | - | | Lam Dong, Vietnam | | (Van Nguyen et al., 2018) |
|  | - | | - | *Usnea ceratina* (Lichen thalli) | *-* | | Paksong town Paksong district, Champasack, Laos, Vietnam | | (Bui et al., 2022) |
| Salazinin A (**48**) | 416 | | C_20_H_16_O_10_ | *Parmelia saxatilis* (Lichen) | - | | Hanshan National Nature Reserve Tongliao, Inner Mongolia, China | | (Bai et al., 2021) |
|  | - | | - | *Cladonia metacorallifera* (Antarctic lichen) | - | | Peninsula Fildem, King George Island, Antarctica | | (Sepúlveda et al., 2022) |
| Galbinic acid (**49**) | | 430 | C_20_H_14_O_11_ | *Parmotrema dilatatum* (Lichen thalli) | - | | Rocks in Lam Dong, Vietnam | (Devi et al., 2020) | |
|  | | - | - | *Usnea subfloridana* Stirton (Lichen) | Barks of alpine trees | | Coimbatore-Ooty-Gundlupet Hwy, Tamil Nadu, India | (Nguyen et al. 2021) | |
| Variolaric acid (**50**) | | 314 | C_16_H_10_O_7_ | Genus *Ramalina* (Lichen) | - | | - | (Bay et al., 2020) | |
| Excelsione = Phomopsidone (**51**) | | 358 | C_18_H_14_O_8_ | *Melastoma malabathricum* (Plant, Roots) | - | | Yanshan Town Guilin, China | (He et al., 2022) | |
| Diffractione B (**52**) | | 444 | C_22_H_20_O_10_ | *Parmelia saxatilis* (Lichen) | - | | Hanshan National Nature Reserve Tongliao, Inner Mongolia, China | (Bai et al., 2021) | |
| Parmosidone F (**53**) | | 428 | C_22_H_20_O_9_ | *Parmotrema tsavoense* (Lichen) | - | | Rocks on Ta Cu Mountain, Ham Thuan Nam district, Binh Thuan, Vietnam | (Duong et al., 2020) | |
| Guanxidone A (**55**) | | 372 | C_19_H_16_O_8_ | *Melastoma malabathricum* (Plant, Roots) | - | | Yanshan Town Guilin, China | (He et al., 2022) | |
|  | | - | - | *Aspergillus* sp. GXNU-A9 (Fungus) | *Acanthus ilicifolius* (Mangrove plant) | | Seaside of Qinzhou, Guangxi, China | (Hao et al., 2022) | |
| Guanxidone B (**56**) | | 342 | C_18_H_14_O_7_ | *Melastoma malabathricum* (Plant, Roots) | - | | Yanshan Town Guilin, China | (He et al., 2022) | |
| Parmosidone F1 (**54**)* | | 494 | C_25_H_18_O_11_ | *Parmotrema dilatatum* (Lichen thalli) | - | | Rocks in Lam Dong, Vietnam | (Devi et al., 2020) | |
| Connorstictic acid (**57**) | | 358 | C_18_H_14_O_8_ | *Usnea baileyi* (Lichen) | - | | Lam Dong, Vietnam | (Van Nguyen et al., 2018) | |
|  | | - | - | Genus *Ramalina* (Lichen) | - | | - | (Bay et al., 2020) | |
|  | | - | - | *Cladonia metacorallifera* (Antarctic lichen) | - | | Peninsula Fildem, King George Island, Antarctica | (Sepúlveda et al., 2022) | |
| Ceratinalone (**58**) | | 402 | C_20_H_18_O_9_ | *Usnea ceratina* (Lichen thalli) | *-* | | Paksong town Paksong district, Champasack, Laos, Vietnam | (Bui et al., 2022) | |
| Stictic acid = Scopuloric acid (**59**) | | 386 | C_19_H_14_O_9_ | *Usnea baileyi* (Lichen) | - | | Lam Dong, Vietnam | (Van Nguyen et al., 2018) | |
|  | | - | - | *Meiogyne baillonii* (Plant, Bark) | - | | Nodela Valle, New Caledonia | (Olivon et al., 2018) | |
|  | | - | - | Genus *Ramalina* (Lichen) | - | | - | (Bay et al., 2020) | |
|  | | - | - | *Stereocaulon montagneanum* (Lichen) | - | | Sirukam, Solok, West Sumatra, Indonesia | (Ismed et al., 2021) | |
|  | | - | - | *Stereocaulon montagneanum* (Lichen) | - | | Malalak, West Sumatra, Indonesia | (Ismed et al., 2021) | |
|  | | - | - | *Usnea ceratina* (Lichen thalli) | *-* | | Paksong town Paksong district, Champasack, Laos, Vietnam | (Bui et al., 2022) | |
| Norstictic acid (**60**) | | 372 | C_18_H_12_O_9_ | Genus *Ramalina* (Lichen) | - | | - | (Bay et al., 2020) | |
|  | | - | - | *Usnea fulvoreagens* (Lichen) | - | | Skyline Trail in Sibley Volcanic Regional Preserve, Alameda County, California, U.S.A. | (Burt et al., 2022) | |
| 8`-O-Methylstictic acid (**61**) | | 400 | C_20_H_16_O_9_ | *Usnea baileyi* (Lichen) | - | | Lam Dong, Vietnam | (Van Nguyen et al., 2018) | |
|  | | - | - | *Hypotrachyna caraccensis* (Lichen) | *-* | | Páramo de Sumapaz, Bogotá Colombia | (Leal et al., 2018) | |
|  | | - | - | *Usnea ceratina* (Lichen thalli) | *-* | | Paksong town Paksong district, Champasack, Laos, Vietnam | (Bui et al., 2022) | |
| 8`-O-Ethylstictic acid (**62**) | | 414 | C_21_H_18_O_9_ | *Usnea ceratina* (Lichen thalli) | *-* | | Paksong town Paksong district, Champasack, Laos, Vietnam | (Bui et al., 2022) | |
| Neotricone (**63**) | | 372 | C_18_H_12_O_9_ | *Usnea fulvoreagens* (Lichen) | - | | Skyline Trail in Sibley Volcanic Regional Preserve, Alameda County, California, U.S.A. | (Burt et al., 2022) | |
| Conneotricone (**64**) | | 388 | C_18_H_12_O_10_ | *Usnea fulvoreagens* (Lichen) | - | | Skyline Trail in Sibley Volcanic Regional Preserve, Alameda County, California, U.S.A. | (Burt et al., 2022) | |
| Salazinic acid (**65**) | | 388 | C_18_H_12_O_10_ | *Parmotrema dilatatum* (Lichen thalli) | - | | Rocks in Lam Dong, Vietnam | (Duong, 2019) | |
|  | | - | - | *Parmotrema tinctorum* (Lichen) | *-* | | Horsely Hills of Eastern Ghats, Chittor, Andhra Pradesh, India | (Pavan Kumar et al., 2020) | |
|  | | - | - | *Parmotrema dilatatum* (Lichen thalli) | - | | Rocks in Lam Dong, Vietnam | (Devi et al., 2020) | |
|  | | - | - | Genus *Ramalina* (Lichen) | - | | - | (Bay et al., 2020) | |
|  | | - | - | *Parmelia saxatilis* (Lichen) | - | | Hanshan National Nature Reserve Tongliao, Inner Mongolia, China | (Bai et al., 2021) | |
|  | | - | - | *Usnea subfloridana* Stirton (Lichen) | Barks of alpine trees | | Coimbatore-Ooty-Gundlupet Hwy, Tamil Nadu, India | (Nguyen et al. 2021) | |
|  | | - | - | *Usnea fulvoreagens* (Lichen) | - | | Skyline Trail in Sibley Volcanic Regional Preserve, Alameda County, California, U.S.A. | (Burt et al., 2022) | |
|  | | - | - | *Usnea laevis* Nyl (Lichin) | Tree barks | | Western Ghats mountains, Udhagamandalam (Nilgiris District), Tamil Nadu, India | (Tatipamula and Annam, 2022) | |
| Perisalazinic acid (**66**) | | 404 | C_18_H_12_O_11_ | *Usnea fulvoreagens* (Lichen) | - | | Skyline Trail in Sibley Volcanic Regional Preserve, Alameda County, California, U.S.A. | (Burt et al., 2022) | |
| 8’-O-methylsalazinic acid **(67)** | | 402 | C_19_H_14_O_10_ | *Parmotrema dilatatum* (Lichen thalli) | - | | Rocks in Lam Dong, Vietnam | (Duong, 2019) | |
|  | | - | - | *Parmotrema dilatatum* (Lichen thalli) | - | | Rocks in Lam Dong, Vietnam | (Devi et al., 2020) | |
| Norperistictic acid (**68**) | | 346 | C_18_H_18_O_7_ | *Usnea longissima* (Beard lichen) | - | | Sichuan, Yunnan, Zhejiang, and Shanxi, China | (Jin et al., 2018) | |
| Menegazziaic acid (**69**) | | 374 | C_18_H_14_O_9_ | *Usnea longissima* (Beard lichen) | - | | Sichuan, Yunnan, Zhejiang, and Shanxi, China | (Jin et al., 2018) | |
|  | | - | - | *Usnea baileyi* (Lichen) | - | | Lam Dong, Vietnam | (Van Nguyen et al., 2018) | |
| 1-Hydroxy-11-(hydroxymethyl)-4,10-dimethoxy-5,8-dimethyl-7H-benzo[6,7][1,4]dioxepino [2,3-e]  isobenzofuran-3,7(1H)-dione (**70**) | | 402 | C_20_H_18_O_9_ | *Usnea longissima* (Beard lichen) | - | | Sichuan, Yunnan, Zhejiang, and Shanxi, China | (Jin et al., 2018) | |
| 3′-*O-*Demethylcryptostictinolide (**71**) | | 358 | C_18_H_14_O_8_ | *Usnea baileyi* (Lichen thalli) | Bark of trees | | Tam Bo Mountain, Di Linh district, Lam Dong, Vietnam | (Nguyen et al., 2020) | |
| Cryptostictic acid (**72**) | | 388 | C_19_H_16_O_9_ | *Usnea baileyi* (Lichen) | - | | Lam Dong, Vietnam | (Van Nguyen et al., 2018) | |
|  | | - | - | Genus *Ramalina* (Lichen) | - | | - | (Bay et al., 2020) | |
|  | | - | - | *Cladonia metacorallifera* (Antarctic lichen) | - | | Peninsula Fildem, King George Island, Antarctica | (Sepúlveda et al., 2022) | |
| Peristictic acid (**73)** | | 402 | C_19_H_14_O_10_ | Genus *Ramalina* (Lichen) | - | | - | (Bay et al., 2020) | |
| Parmosidone C (**74**) | | 552 | C_28_H_24_O_12_ | *Parmotrema tsavoense* (Lichen) | - | | Rocks on Ta Cu Mountain, Ham Thuan Nam district, Binh Thuan, Vietnam | (Duong et al., 2015) | |
| Parmosidone D (**75**) | | 538 | C_27_H_22_O_12_ | *Parmotrema tsavoense* (Lichen) | - | | Rocks on Ta Cu Mountain, Ham Thuan Nam district, Binh Thuan, Vietnam | (Duong et al., 2015) | |
| Parmosidone G (**76**)* | | 508 | C_27_H_24_O_10_ | *Parmotrema tsavoense* (Lichen) | - | | Rocks on Ta Cu Mountain, Ham Thuan Nam district, Binh Thuan, Vietnam | (Duong et al., 2020) | |
| Parmosidone I (**77**) | | 566 | C_28_H_22_O_13_ | *Parmotrema tsavoense* (Lichen) | - | | Rocks on Ta Cu Mountain, Ham Thuan Nam district, Binh Thuan, Vietnam | (Duong et al., 2020) | |
| Parmosidone G1 (**78**)* | | 534 | C_28_H_22_O_11_ | *Parmotrema dilatatum* (Lichen thalli) | - | | Rocks in Lam Dong, Vietnam | (Devi et al., 2020) | |
| Parmosidone H (**79**) | | 548 | C_30_H_28_O_10_ | *Parmotrema tsavoense* (Lichen) | - | | Rocks on Ta Cu Mountain, Ham Thuan Nam district, Binh Thuan, Vietnam | (Duong et al., 2020) | |
| Parmosidone J (**80**) | | 428 | C_22_H_20_O_9_ | *Parmotrema tsavoense* (Lichen) | - | | Rocks on Ta Cu Mountain, Ham Thuan Nam district, Binh Thuan, Vietnam | (Duong, T. et al., 2020) | |
| Parmosidone K (**81**) | | 480 | C_25_H_20_O_10_ | *Parmotrema tsavoense* (Lichen) | - | | Rocks on Ta Cu Mountain, Ham Thuan Nam district, Binh Thuan, Vietnam | (Nguyen et al., 2022a) | |
| Boremexin A (**82**) | | 398 | C_21_H_18_O_8_ | *Boeremia exigua* (Fungus) | *Solanum tuberosum* (Potato) | | Lincang, Yunnan, China | (Chen et al., 2020) | |
| Boremexin B (**83**) | | 398 | C_21_H_18_O_8_ | *Boeremia exigua* (Fungus) | *Solanum tuberosum* (Potato) | | Lincang, Yunnan, China | (Chen et al., 2020) | |
| Boremexin C (**84**) | | 416 | C_21_H_20_O_9_ | *Boeremia exigua* (Fungus) | *Solanum tuberosum* (Potato) | | Lincang, Yunnan, China | (Chen et al., 2020) | |
| Hyperwightin A (**85**) | | 324 | C_19_H_16_O_5_ | *Hypericum wightianum* (Whale plant) | *-* | | Tengchong, Yunnan, China | (Yang et al., 2019) | |
| Diaporthol B (**86**) | | 360 | C_20_H_18_O_5_ | *Diaporthe* sp. ECN-137 | *Phellodendron amurense* (Leaves) | | Herbal Garden of Gifu Pharmaceutical Universit, Gifu, Japan | (Nakashima et al., 2018) | |
| Glomellonic acid (**87**) | | 458 | C_21_H_14_O_12_ | *Usnea longissima* (Beard lichen) | - | | Sichuan, Yunnan, Zhejiang, and Shanxi, China | (Jin et al., 2018) | |
| Botryorhodine H (**88**) | | 388 | C_22_H_18_O_6_ | *Trichoderma* sp. 307 co-culturing with *Acinetobacter johnsoni*i B2 (Fungus) | *Clerodendrum inerme* (Mangrove plant) | | Zhanjiang Mangrove National Nature Reserve, Guangdong, China | (Zhang et al., 2018) | |
| Simplicildone J (**89**) | | 420 | C25H24O6 | *Simplicillium lanosoniveum* PSU-H168 and PSU-H261  (Fungus) | *Hevea brasiliensis* (Plant, Leaves) | | Songkhla, Thailand | (Rukachaisirikul et al., 2019) | |
| Simplicildone K (**90**) | | 546 | C32H34O8 | *Simplicillium lanosoniveum* PSU-H168 and PSU-H261  (Fungus) | *Hevea brasiliensis* (Plant, Leaves) | | Songkhla, Thailand | (Rukachaisirikul et al., 2019) | |
| Mollicellin B (**91**) | | 382 | C_21_H_18_O_7_ | *Chaetomium brasiliense* (Fungus) | Thai rice (Stems) | | Thailand | (Promgool et al., 2022) | |
| Mollicellin C (**92**) | | 412 | C_22_H_20_O_8_ | *Chaetomium brasiliense* (Fungus) | Thai rice (Stems) | | Thailand | (Promgool et al., 2022) | |
| Mollicellin D (**93**) | | 404 | C_21_H_21_ClO_6_ | *Chaetomium* *brasiliense* SD-596 (Fungus) | Soil | | Miyaluo Scenic Spot, Aba City, Sichuan, China | (Zhao, P. et al., 2021) | |
| Mollicellin E (**94**) | | 446 | C_22_H_19_ClO_8_ | *Chaetomium brasiliense* (Fungus) | Thai rice (Stems) | | Thailand | (Promgool et al., 2022) | |
| Mollicellin F (**95**) | | 432 | C_21_H_17_ClO_8_ | *Chaetomium brasiliense* (Fungus) | Thai rice (Stems) | | Thailand | (Promgool et al., 2022) | |
| Mollicellin G (**96**) | | 368 | C_21_H_20_O_6_ | *Chaetomium* sp. Eef-10 (Fungus) | *Eucalyptus exserta* | | Guangdong China | (Ouyang et al., 2018) | |
| Mollicellin H (**97**) | | 368 | C_21_H_20_O_6_ | *Chaetomium* sp. Eef-10 (Fungus) | *Eucalyptus exserta* | | Guangdong China | (Ouyang et al., 2018) | |
|  | | - | - | *Chaetomium* *brasiliense* SD-596 (Fungus) | Soil | | Soil in the Miyaluo Scenic Spot, Aba City, Sichuan, China | (Zhao et al., 2021) | |
|  | | - | - | *Chaetomium brasiliense* (Fungus) | Thai rice (Stems) | | Thailand | (Promgool et al., 2022) | |
| Mollicellin I (**98**) | | 370 | C_21_H_22_O_6_ | *Chaetomium* sp. Eef-10 (Fungus) | *Eucalyptus exserta* | | Guangdong China | (Ouyang et al., 2018) | |
| Mollicellin M (**99**) | | 416 | C_21_H_17_ClO_7_ | *Chaetomium brasiliense* (Fungus) | Thai rice (Stems) | | Thailand | (Promgool et al., 2022) | |
| Mollicellin N (**100**) | | 398 | C_21_H_18_O_8_ | *Chaetomium brasiliense* (Fungus) | Thai rice (Stems) | | Thailand | (Promgool et al., 2022) | |
| Mollicellin O (**101**) | | 398 | [C_23_H_26_O_6_](https://pubchem.ncbi.nlm.nih.gov/#query=C23H26O6) | *Chaetomium* sp. Eef-10 (Fungus) | *Eucalyptus exserta* | | Guangdong China | (Ouyang et al., 2018) | |
| Mollicellin P (**102**) | | 430 | C_23_H_26_O_8_ | *Chaetomium* sp. Eef-10 (Fungus) | *Eucalyptus exserta* | | Guangdong China | (Ouyang et al., 2018) | |
| Mollicellin Q (**103**) | | 414 | C_23_H_26_O_7_ | *Chaetomium* sp. Eef-10 (Fungus) | *Eucalyptus exserta* | | Guangdong China | (Ouyang et al., 2018) | |
| Mollicellin R (**104**) | | 366 | C_21_H_18_O_6_ | *Chaetomium* sp. Eef-10 (Fungus) | *Eucalyptus exserta* | | Guangdong China | (Ouyang et al., 2018) | |
|  | | - | - | *Chaetomium brasiliense* (Fungus) | Thai rice (Stems) | | Thailand | (Promgool et al., 2022) | |
| Mollicellin S (**105**) | | 416 | C_22_H_21_ClO_6_ | *Chaetomium* *brasiliense* SD-596 (Fungus) | Soil | | Soil in the Miyaluo Scenic Spot, Aba City, Sichuan, China | (Zhao et al., 2021) | |
| Mollicellin T (**106**) | | 418 | C_22_H_23_ClO_6_ | *Chaetomium* *brasiliense* SD-596 (Fungus) | Soil | | Soil in the Miyaluo Scenic Spot, Aba City, Sichuan, China | (Zhao et al., 2021) | |
| Mollicellin U (**107**) | | 340 | C_20_H_20_O_5_ | *Chaetomium* *brasiliense* SD-596 (Fungus) | Soil | | Soil in the Miyaluo Scenic Spot, Aba City, Sichuan, China | (Zhao et al., 2021) | |
| Mollicellin V (**108**) | | 384 | C_21_H_20_O_7_ | *Chaetomium brasiliense* (Fungus) | Thai rice (Stems) | | Thailand | (Promgool et al., 2022) | |
| Mollicellin W (**109**) | | 412 | C_23_H_24_O_7_ | *Chaetomium brasiliense* (Fungus) | Thai rice (Stems) | | Thailand | (Promgool et al., 2022) | |
| Mollicellin X (**110**) | | 446 | C_23_H_23_ClO_7_ | *Chaetomium brasiliense* (Fungus) | Thai rice (Stems) | | Thailand | (Promgool et al., 2022) | |
| Mollicellin Y (**111**) | | 412 | C_23_H_24_O_7_ | *Chaetomium brasiliense* (Fungus) | Thai rice (Stems) | | Thailand | (Promgool et al., 2022) | |
| Auranticin A (**112**) | | 440 | C_24_H_24_O_8_ | *Pycnidiophora dispersa* (Fungus) | Wetland-soil samples | | Baiyangdian Lake, Hebei, China | (Zhao et al., 2020) | |
| Auranticin B (**113**) | | 438 | C_24_H_22_O_8_ | *Pycnidiophora dispersa* (Fungus) | Wetland-soil samples | | Baiyangdian Lake, Hebei, China | (Zhao et al., 2020) | |
| Pilobolusone C (**114**) | | 454 | C_25_H_26_O_8_ | *Pycnidiophora dispersa* (Fungus) | Wetland-soil samples | | Baiyangdian Lake, Hebei, China | (Zhao et al., 2020) | |
| Spiromastixone A (**115**) | | 328 | C_19_H_20_O_5_ | *Spiromastix* sp. MY-1 (Fungus) | *Monomorium chinensis* | | Suburb of Nanjing, Jiangsu, China | (Guo et al., 2022) | |
| Spiromastixone B (**116**) | | 362 | C_19_H_19_ClO_5_ | *Spiromastix* sp. MY-1 (Fungus) | *Monomorium chinensis* | | Suburb of Nanjing, Jiangsu, China | (Guo et al., 2022) | |
| Spiromastixone E (**117**) | | 396 | C_19_H_18_Cl_2_O_5_ | *Spiromastix* sp. MY-1 (Fungus) | *Monomorium chinensis* | | Suburb of Nanjing, Jiangsu, China | (Guo et al., 2022) | |
| Spiromastixone F (**118**) | | 430 | C_19_H_17_Cl_3_O_5_ | *Spiromastix* sp. MY-1 (Fungus) | *Monomorium chinensis* | | Suburb of Nanjing, Jiangsu, China | (Guo et al., 2022) | |
| Spiromastixone G (**119**) | | 444 | C_20_H_19_Cl_3_O_5_ | *Spiromastix* sp. MY-1 (Fungus) | *Monomorium chinensis* | | Suburb of Nanjing, Jiangsu, China | (Guo et al., 2022) | |
| Spiromastixone I (**120**) | | 463 | C_19_H_16_Cl_4_O_5_ | *Spiromastix* sp. MY-1 (Fungus) | *Monomorium chinensis* | | Suburb of Nanjing, Jiangsu, China | (Guo et al., 2022) | |
| Spiromastixone P (**121**)* | | 406 | C_19_H_19_BrO_5_ | *Spiromastix* sp. MY-1 (Fungus) | *Monomorium chinensis* | | Suburb of Nanjing, Jiangsu, China | (Guo et al., 2022) | |
| Spiromastixone P1 (**122**)* | | 362 | C_19_H_19_ClO_5_ | *Spiromastix* sp. MCCC 3A00308 | Deep-sea sediment | | South Atlantic Ocean | (Niu et al., 2021a) | |
| Spiromastixone Q (**123**)* | | 406 | C_19_H_19_BrO_5_ | *Spiromastix* sp. MY-1 (Fungus) | *Monomorium chinensis* | | Suburb of Nanjing, Jiangsu, China | (Guo et al., 2022) | |
| Spiromastixone Q1 (**124**)* | | 362 | C_19_H_19_ClO_5_ | *Spiromastix* sp. MCCC 3A00308 | Deep-sea sediment | | South Atlantic Ocean | (Niu et al., 2021a) | |
| Spiromastixone R (**125**)* | | 406 | C_19_H_19_BrO_5_ | *Spiromastix* sp. MY-1 (Fungus) | *Monomorium chinensis* | | Suburb of Nanjing, Jiangsu, China | (Guo et al., 2022) | |
| Spiromastixone R1 (**126**)* | | 430 | C_19_H_17_Cl_3_O_5_ | *Spiromastix* sp. MCCC 3A00308 | Deep-sea sediment | | South Atlantic Ocean | (Niu et al., 2021a) | |
| Spiromastixone S (**127**)* | | 483 | C_19_H_18_Br_2_O_5_ | *Spiromastix* sp. MY-1 (Fungus) | *Monomorium chinensis* | | Suburb of Nanjing, Jiangsu, China | (Guo et al., 2022) | |
| Spiromastixone S1 (**128**)* | | 372 | C_20_H_20_O_7_ | *Spiromastix* sp. MCCC 3A00308 | Deep-sea sediment | | South Atlantic Ocean | (Niu et al., 2021a) | |
| Spiromastixone T (**129**) | | 497 | C_20_H_20_Br_2_O_5_ | *Spiromastix* sp. MY-1 (Fungus) | *Monomorium chinensis* | | Suburb of Nanjing, Jiangsu, China | (Guo et al., 2022) | |
| Himantormione A (**130**) | | 356 | C_21_H_24_O_5_ | *Himantormia lugubris* (Antarctic lichen) | *-* | | King George Island Antarctica | (Hyung Koo et al., 2022) | |
| Himantormione B (**131**) | | 384 | C_23_H_28_O_5_ | *Himantormia lugubris* (Antarctic lichen) | *-* | | King George Island Antarctica | (Hyung Koo et al., 2022) | |
| Norcolensoic acid (**132**) | | 428 | C_24_H_28_O_7_ | *Lachnum virgineum* (Fungus, inedible mushroom) | *-* | | Foot of Mt. Gassan, Yamagata, Prefecture, Japan | (Shiono et al., 2018) | |
| Norlobaridone (**133**) | | 398 | C_23_H_26_O_6_ | *Usnea longissima* (Beard lichen) | - | | Sichuan, Yunnan, Zhejiang, and Shanxi, China | (Jin et al., 2018) | |
|  | | - | - | *Parmotrema tinctorum* (Lichen) | *-* | | Horsely Hills of Eastern Ghats, Chittor, Andhra Pradesh, India | (Pavan Kumar et al., 2020) | |
| Physodic acid (**134**) | | 470 | C_26_H_30_O_8_ | *Usnea longissima* (Beard lichen) | - | | Sichuan, Yunnan, Zhejiang, and Shanxi, China | (Jin et al., 2018) | |
|  | | - | - | Genus *Ramalina* (Lichen) | - | | - | (Bay et al., 2020) | |
|  | | - | - | *Pseudevernia furfuracea* (Lichen) | *-* | | - | (Singh et al., 2021a) | |
|  | | - | - | *Hypogymnia physodes* (Lichen) | - | | Jastrz˛ebsko Stare, Poland | (Studzińska-Sroka et al., 2021) | |
|  | | - | - | *Diploschistes diacapsis* (Lichen) | Clay soils | | Maraveh Tapeh Road in Golestan, Iran | (Sedrpoushan et al., 2022) | |
| 3-Hydroxyphysodic acid (**135**) | | 486 | C_26_H_30_O_9_ | *Hypogymnia physodes* (Lichen) | - | | Jastrz˛ebsko Stare, Poland | (Studzińska-Sroka et al., 2021) | |
| Lobaric acid (**136**) | | 456 | C_25_H_28_O_8_ | *Stereocaulon paschale* (Nordic lichen) | - | | Umiujaq, Quebec, Canada | (Carpentier et al., 2018) | |
|  | | - | - | *Usnea longissima* (Beard lichen) | - | | Sichuan, Yunnan, Zhejiang, and Shanxi, China | (Jin et al., 2018) | |
|  | | - | - | *Stereocaulon montagneanum* (Lichen) | - | | Sirukam, Solok, West Sumatra, Indonesia | (Ismed et al., 2021) | |
|  | | - | - | *Stereocaulon montagneanum* (Lichen) | - | | Malalak, West Sumatra, Indonesia | (Ismed et al., 2021) | |
|  | | - | - | *Usnea subfloridana* Stirton (Lichen) | Barks of alpine trees | | Coimbatore-Ooty-Gundlupet Hwy, Tamil Nadu, India | (Nguyen et al. 2021) | |
|  | | - | - | *Swietenia mahogany* (Lichen) | *-* | | Klocing, Bondowoso Regency, East Java, Indonesia | (Nugraha et al., 2022) | |
|  | | - | - | *Cladonia metacorallifera* (Antarctic lichen) | - | | Peninsula Fildem, King George Island, Antarctica | (Sepúlveda et al., 2022) | |
|  | | - | - | *Usnea laevis* Nyl (Lichin) | Tree barks | | Western Ghats mountains, Udhagamandalam (Nilgiris District), Tamil Nadu, India | (Tatipamula and Annam, 2022) | |
| Oxolobaric acid (**137**) | | 470 | C_25_H_26_O_9_ | *Usnea longissima* (Beard lichen) | - | | Sichuan, Yunnan, Zhejiang, and Shanxi, China | (Jin et al., 2018) | |
| Unguinol = Tridechloronornidulin (**138**) | | 326 | C_18_H_19_O_5_ | *Aspergillus unguis* PSU-RSPG204 (Fungus) | Soil | | Surat Thani, Thailand | (Phainuphong et al., 2018) | |
|  | | - | - | *Aspergillus unguis* DLEP2008001 | Seaweed | | Dalian, China | (Yang et al., 2018) | |
|  | | - | - | *Aspergillus unguis* 158SC-067 (Fungus) | Seawater | | Near the Socheongcho Ocean Research Station, Korea | (Anh et al., 2022) | |
|  | | - | - | *Aspergillus unguis* BCC54176 (Fungus) | *Coriandrum sativum* (Leaf) | | Ying Charoen market, north Bangkok, Thailand | (Sadorn et al., 2022) | |
|  | | - | - | *Aspergillus unguis* PSU-MF16 (Fungus) | *Dysidea* (Sponge) | | Koh Bulon Mai Pai, Satun, Thailand | (Saetang et al., 2021) | |
| 2-Chlorounguinol (**139**) | | 360 | C_19_H_17_ClO_5_ | *Aspergillus unguis* PSU-RSPG204 (Fungus) | Soil | | Surat Thani, Thailand | (Phainuphong et al., 2018) | |
|  | | - | - | *Aspergillus unguis* DLEP2008001 | Seaweed | | Dalian, China | (Yang et al., 2018) | |
|  | | - | - | *Aspergillus unguis* 158SC-067 (Fungus) | Seawater | | Near the Socheongcho Ocean Research Station, Korea | (Anh et al., 2022) | |
|  | | - | - | *Aspergillus unguis* BCC54176 (Fungus) | *Coriandrum sativum* (Leaf) | | Ying Charoen market, north Bangkok, Thailand | (Sadorn et al., 2022) | |
|  | | - | - | *Aspergillus unguis* PSU-MF16 (Fungus) | *Dysidea* (Sponge) | | Koh Bulon Mai Pai, Satun, Thailand | (Saetang et al., 2021) | |
| 2,4-Dichlorounguinol (**140**) | | 394 | C_19_H_15_Cl_2_O_5_ | *Aspergillus unguis* PSU-RSPG204 (Fungus) | Soil | | Surat Thani, Thailand | (Phainuphong et al., 2018) | |
|  | | - | - | *Aspergillus unguis* (Fungus) | Cultured | | - | (Morshed et al., 2018) | |
| 2,7-Dichlorounguinol (**141**) | | 408 | C_20_H_18_Cl_2_O_5_ | *Aspergillus unguis* (Fungus) | Cultured | | - | (Morshed et al., 2018) | |
|  | | - | - | *Aspergillus unguis* BCC54176 (Fungus) | *Coriandrum sativum* (Leaf) | | Ying Charoen market, north Bangkok, Thailand | (Sadorn et al., 2022) | |
| 4,7-Dichlorounguinol (**142**) | | 394 | C_19_H_16_C_l2_O_5_ | *Aspergillus unguis* (Fungus) | Cultured | | - | (Morshed et al., 2018) | |
|  | | - | - | *Aspergillus unguis* BCC54176 (Fungus) | *Coriandrum sativum* (Leaf) | | Ying Charoen market, north Bangkok, Thailand | (Sadorn et al., 2022) | |
| 3,1`-Dichlorounguinol (**143**) | | 394 | C_19_H_16_Cl_2_O_5_ | *Aspergillus unguis* 158SC-067 (Fungus) | Seawater | | Near the Socheongcho Ocean Research Station, Korea | (Anh et al., 2022) | |
| 2-Chloro-7-bromounguinol (**144**) | | 438 | C_19_H_16_BrClO_5_ | *Aspergillus unguis* (Fungus) | Cultured | | - | (Morshed et al., 2018) | |
| 7-Bromounguinol (**145**) | | 404 | C_19_H_17_BrO_5_ | *Aspergillus unguis* (Fungus) | Cultured | | - | (Morshed et al., 2018) | |
| Nidulin (**146**) | | 442 | C_20_H_17_Cl_3_O_5_ | *Aspergillus unguis* PSU-RSPG204 (Fungus) | Soil | | Surat Thani, Thailand | (Phainuphong et al., 2018) | |
|  | | - | - | *Aspergillus unguis* DLEP2008001 | Seaweed | | Dalian, China | (Yang et al., 2018) | |
|  | | - | - | *Aspergillus unguis* (Fungus) | Cultured | | - | (Morshed et al., 2018) | |
|  | | - | - | *Aspergillus unguis* 158SC-067 (Fungus) | Seawater | | Near the Socheongcho Ocean Research Station, Korea | (Anh et al., 2022) | |
|  | | - | - | *Aspergillus unguis* BCC54176 (Fungus) | *Coriandrum sativum* (Leaf) | | Ying Charoen market, north Bangkok, Thailand | (Sadorn et al., 2022) | |
|  | | - | - | *Aspergillus unguis* PSU-MF16 (Fungus) | *Dysidea* (Sponge) | | Koh Bulon Mai Pai, Satun, Thailand | (Saetang et al., 2021) | |
| Nornidulin = Ustin (**147**) | | 428 | C_19_H_15_Cl_3_O_5_ | *Aspergillus unguis* PSU-RSPG204 (Fungus) | Soil | | Surat Thani, Thailand | (Phainuphong et al., 2018) | |
|  | | - | - | *Aspergillus unguis* DLEP2008001 | Seaweed | | Dalian, China | (Yang et al., 2018) | |
|  | | - | - | *Aspergillus unguis* (Fungus) | Cultured | | - | (Morshed et al., 2018) | |
|  | | - | - | *Aspergillus unguis* 158SC-067 (Fungus) | Seawater | | Near the Socheongcho Ocean Research Station, Korea | (Anh et al., 2022) | |
|  | | - | - | *Aspergillus unguis* BCC54176 (Fungus) | *Coriandrum sativum* (Leaf) | | Ying Charoen market, north Bangkok, Thailand | (Sadorn et al., 2022) | |
|  | | - | - | *Aspergillus unguis* GXIMD 02505 (Fungus) | *Pocillopora damicornis* (Coral) | | Weizhou Islands coral reef in Guangxi Zhuang autonomous region, China | (Zhang et al., 2022) | |
|  | | - | - | *Aspergillus unguis* PSU-MF16 (Fungus) | *Dysidea* (Sponge) | | Koh Bulon Mai Pai, Satun, Thailand | (Saetang et al., 2021) | |
| Aspergillusidone A (**148**) | | 370 | C_20_H_18_O_7_ | *Aspergillus unguis* PSU-RSPG204 (Fungus) | Soil | | Surat Thani, Thailand | (Phainuphong et al., 2018) | |
|  | | - | - | *Aspergillus unguis* (Fungus) | Cultured | | - | (Morshed et al., 2018) | |
|  | | - | - | *Aspergillus unguis* DLEP2008001 | Seaweed | | Dalian, China | (Yang et al., 2018) | |
|  | | - | - | *Aspergillus unguis* PSU-MF16 (Fungus) | *Dysidea* (Sponge) | | Koh Bulon Mai Pai, Satun, Thailand | (Saetang et al., 2021) | |
|  | | - | - | *Aspergillus unguis* BCC54176 (Fungus) | *Coriandrum sativum* (Leaf) | | Ying Charoen market, north Bangkok, Thailand | (Sadorn et al., 2022) | |
| Aspergillusidone B (**149**) | | 408 | C_20_H_18_ClO_5_ | *Aspergillus unguis* PSU-RSPG204 (Fungus) | Soil | | Surat Thani, Thailand | (Phainuphong et al., 2018) | |
|  | | - | - | *Aspergillus unguis* (Fungus) | Cultured | | - | (Morshed et al., 2018) | |
|  | | - | - | *Aspergillus unguis* PSU-MF16 (Fungus) | *Dysidea* (Sponge) | | Koh Bulon Mai Pai, Satun, Thailand | (Saetang et al., 2021) | |
|  | |  |  | *Aspergillus unguis* BCC54176 (Fungus) | *Coriandrum sativum* (Leaf) | | Ying Charoen market, north Bangkok, Thailand | (Sadorn et al., 2022) | |
|  | | - | - | *Aspergillus unguis* 158SC-067 (Fungus) | Seawater | | Near the Socheongcho Ocean Research Station, Korea | (Anh et al., 2022) | |
|  | | - | - | *Aspergillus unguis* GXIMD 02505 (Fungus) | *Pocillopora damicornis* (Coral) | | Weizhou Islands coral reef, Guangxi Zhuang autonomous region, China | (Zhang et al., 2022) | |
| Aspergillusidone C (**150**) | | 394 | C_19_H_16_ClO_5_ | *Aspergillus unguis* PSU-RSPG204 (Fungus) | Soil | | Surat Thani, Thailand | (Phainuphong et al., 2018) | |
|  | | - | - | *Aspergillus unguis* (Fungus) | Cultured | | - | (Morshed et al., 2018) | |
|  | | - | - | *Aspergillus unguis* DLEP2008001 | Seaweed | | Dalian, China | (Yang et al., 2018) | |
|  | | - | - | *Aspergillus unguis* PSU-MF16 (Fungus) | *Dysidea* (Sponge) | | Koh Bulon Mai Pai, Satun, Thailand | (Saetang et al., 2021) | |
|  | | - | - | *Aspergillus unguis* BCC54176 (Fungus) | *Coriandrum sativum* (Leaf) | | Ying Charoen market, north Bangkok, Thailand | (Sadorn et al., 2022) | |
|  | | - | - | *Aspergillus unguis* GXIMD 02505 (Fungus) | *Pocillopora damicornis* (Coral) | | Weizhou Islands coral reef, Guangxi Zhuang autonomous region, China | (Zhang et al., 2022) | |
| Aspergillusidone D (**151**) | | 384 | C_19_H_16_Br_2_O_5_ | *Aspergillus unguis* (Fungus) | Cultured | | - | (Morshed et al., 2018) | |
| Aspergillusidone E (**152**) | | 404 | C_19_H_17_BrO_5_ | *Aspergillus unguis* (Fungus) | Cultured | | - | (Morshed et al., 2018) | |
| Aspergillusidone F (**153**) | | 498 | C_20_H_18_Br_2_O_5_ | *Aspergillus unguis* DLEP2008001 | Seaweed | | Dalian, China | (Yang et al., 2018) | |
|  | | - | - | *Aspergillus unguis* (Fungus) | Cultured | | - | (Morshed et al., 2018) | |
| Aspergillusidone H (**154**) | | 388 | C_21_H_21_ClO_5_ | *Aspergillus unguis* GXIMD 02505 (Fungus) | *Pocillopora damicornis* (Coral) | | Weizhou Islands coral reef in Guangxi Zhuang autonomous region, China | (Zhang et al., 2022) | |
| Aspersidone (**155**) | | 408 | C_20_H_18_Cl_2_O_5_ | *Aspergillus unguis* PSU-RSPG204 (Fungus) | Soil | | Surat Thani, Thailand | (Phainuphong et al., 2018) | |
|  | | - | - | *Aspergillus unguis* 158SC-067 (Fungus) | Seawater | | Near the Socheongcho Ocean Research Station, Korea | (Anh et al., 2022) | |
| Aspersidone B (**156**) | | 374 | C_20_H_19_ClO_5_ | *Aspergillus unguis* 158SC-067 (Fungus) | Seawater | | Near the Socheongcho Ocean Research Station, Korea | (Anh et al., 2022) | |
| Folipastatin (**157**) | | 380 | C_23_H_24_O_5_ | *Aspergillus unguis* PSU-RSPG204 (Fungus) | Soil | | Surat Thani, Thailand | (Phainuphong et al., 2018) | |
|  | | - | - | *Aspergillus unguis* (Fungus) | Cultured | | - | (Morshed et al., 2018) | |
|  | | - | - | *Aspergillus unguis* PSU-MF16 (Fungus) | *Dysidea* (Sponge) | | Koh Bulon Mai Pai, Satun, Thailand | (Saetang et al., 2021) | |
|  | | - | - | *Aspergillus unguis* 158SC-067 (Fungus) | Seawater | | Near the Socheongcho Ocean Research Station, Korea | (Anh et al., 2022) | |
|  | | - | - | *Aspergillus unguis* BCC54176 (Fungus) | *Coriandrum sativum* (Leaf) | | Ying Charoen market, north Bangkok, Thailand | (Sadorn et al., 2022) | |
| 7-Carboxyfolipastatin (**158**) | | 424 | C_24_H_24_O_7_ | *Aspergillus unguis* (Fungus) | Cultured | | - | (Morshed et al., 2018) | |
| 7-Bromofolipastatin (**159**) | | 458 | C_23_H_23_BrO_5_ | *Aspergillus unguis* (Fungus) | Cultured | | - | (Morshed et al., 2018) | |
| Emeguisin A = 7-Chlorofolipastatin (**160**) | | 414 | C_23_H_23_ClO_5_ | *Aspergillus unguis* (Fungus) | Cultured | | - | (Morshed et al., 2018) | |
|  | | - | - | *Aspergillus unguis* PSU-RSPG204 (Fungus) | Soil | | Surat Thani, Thailand | (Phainuphong et al., 2018) | |
|  | | - | - | *Aspergillus unguis* 158SC-067 (Fungus) | Seawater | | Near the Socheongcho Ocean Research Station, Korea | (Anh et al., 2022) | |
|  | | - | - | *Aspergillus unguis* PSU-MF16 (Fungus) | *Dysidea* (Sponge) | | Koh Bulon Mai Pai, Satun, Thailand | (Saetang et al., 2021) | |
|  | | - | - | *Aspergillus unguis* BCC54176 (Fungus) | *Coriandrum sativum* (Leaf) | | Ying Charoen market, north Bangkok, Thailand | (Sadorn et al., 2022) | |
| Emeguisin B (**161**) | | 428 | C_24_H_25_ClO_5_ | *Aspergillus unguis* (Fungus) | Cultured | | - | (Morshed et al., 2018) | |
|  | | - | - | *Aspergillus unguis* BCC54176 (Fungus) | *Coriandrum sativum* (Leaf) | | Ying Charoen market, north Bangkok, Thailand | (Sadorn et al., 2022) | |
|  | | - | - | *Aspergillus unguis* PSU-MF16 (Fungus) | *Dysidea* (Sponge) | | Koh Bulon Mai Pai, Satun, Thailand | (Saetang et al., 2021) | |
| Emeguisin C (**162**) | | 462 | C_24_H_24_Cl_2_O_5_ | *Aspergillus unguis* (Fungus) | Cultured | | - | (Morshed et al., 2018) | |
|  | | - | - | *Aspergillus unguis* BCC54176 (Fungus) | *Coriandrum sativum* (Leaf) | | Ying Charoen market, north Bangkok, Thailand | (Sadorn et al., 2022) | |
| Emeguisin D (**163**) | | 448 | C_23_H_22_Cl_2_O_5_ | *Aspergillus unguis* BCC54176 (Fungus) | *Coriandrum sativum* (Leaf) | | Ying Charoen market, north Bangkok, Thailand | (Sadorn et al., 2022) | |
| Aspergisidone (**164**) | | 424 | C_24_H_24_O_7_ | *Aspergillus unguis* PSU-RSPG204 (Fungus) | Soil | | Surat Thani, Thailand | (Phainuphong et al., 2018) | |
|  | | - | - | *Aspergillus unguis* PSU-MF16 (Fungus) | *Dysidea* (Sponge) | | Koh Bulon Mai Pai, Satun, Thailand | (Saetang et al., 2021) | |
|  | | - | - | *Aspergillus unguis* BCC54176 (Fungus) | *Coriandrum sativum* (Leaf) | | Ying Charoen market, north Bangkok, Thailand | (Sadorn et al., 2022) | |
| 4-Formyl-3,8-dihydroxy-9-methyl-1,6-bis(1-methyl-1-propenyl)-11*H*-dibenzo[b,e][1,4]dioxepin-11-one  (**165**) | | 394 | C_23_H_22_O_6_ | *Pycnidiophora dispersa* (Fungus) | Wetland-soil samples | | Baiyangdian Lake, Hebei, China | (Zhao et al., 2020) | |
| 3,8-Dihydroxy-4-(hydroxymethyl)-9-methyl-1,6-bis(1-methyl-1-propenyl)- 11*H*-dibenzo[b,e]  [1,4]dioxepin-11-one (**166**) | | 396 | C_23_H_24_O_6_ | *Pycnidiophora dispersa* (Fungus) | Wetland-soil samples | | Baiyangdian Lake, Hebei, China | (Zhao et al., 2020) | |
| 3,8-Dihydroxy-4-(methoxymethyl)-9-methyl-1,6-bis(1-methyl-1-propenyl)-11*H*-dibenzo[b,e][1,4]dioxepin-11-one (**167**) | | 410 | C_24_H_26_O_6_ | *Pycnidiophora dispersa* (Fungus) | Wetland-soil samples | | Baiyangdian Lake, Hebei, China | (Zhao et al., 2020) | |
| (2``R/S)-creticine (**168**) | | 494 | C_29_H_34_O_7_ | *Hypericum kelleri* (Plant, aerial parts) | *-* | | Island of Crete, Greece | (Mathioudaki et al., 2018) | |
| Paucinervin Q (**169**) | | 426 | C_24_H_26_O_7_ | *Garcinia paucinervis* (Stem) | *-* | | Xishuangbanna, Yunnan, China | (Jia et al., 2019) | |
| Parvidepsidone (**170**) | | 508 | C_30_H_36_O_7_ | *Garcinia parvifolia* (Barks) | | *-* | Bulungan Forest, North Kalimantan, Indonesia | (Hartati et al., 2022) | |
| Siphulellic acid (**171**) | | 402 | C_19_H_14_O_10_ | *Cladonia metacorallifera* (Antarctic lichen) | | - | Peninsula Fildem, King George Island, Antarctica | (Seplveda et al., 2022) | |
| Succinprotocetraric acid (**172**) | | 474 | C_22_H_18_O_12_ | *Cladonia metacorallifera* (Antarctic lichen) | | - | Peninsula Fildem, King George Island, Antarctica | (Sepúlveda et al., 2022) | |
| Conprotocetraric acid (**173**) | | 376 | C_18_H_16_O_9_ | *Usnea subfloridana* Stirton (Lichen) | | Barks of alpine trees | Coimbatore-Ooty-Gundlupet Hwy, Tamil Nadu, India | (Nguyen et al. 2021) | |
| Constictic acid (**174**) | | 402 | C_19_H_14_O_10_ | *Usnea subfloridana* Stirton (Lichen) | | Barks of alpine trees | Coimbatore-Ooty-Gundlupet Hwy, Tamil Nadu, India | (Nguyen et al. 2021) | |

*The added number (1) beside the compound name is not original in the reported name, we added it to distinguish these metabolites from the other metabolites reported by different authors having different structure but the same names. M. F.: Molecular formula; M. Wt.: Molecular weight

**Table S2.** Reported antimicrobial activity results of naturally occurring depsidones.

| **Compound name** | **Biological Activity** | **Assay/Organism** | **Biological Results** | | ***Ref.*** |
| --- | --- | --- | --- | --- | --- |
|  |  |  | **Compound** | **Positive Control** |  |
| Mollicellin C (**92**) | Antibacterial | Broth dilution/*B. cereus* | 2.0 μg/mL (MIC) | Kanamycin 2.0 μg/mL (MIC) | (Promgool et al., 2022) |
|  |  | Broth dilution/*B. subtilis* | 2.0 μg/mL (MIC) | Kanamycin 2.0 μg/mL (MIC) | (Promgool et al., 2022) |
| Mollicellin E (**94**) | Antibacterial | Broth dilution/*B. cereus* | 2.0 μg/mL (MIC) | Kanamycin 2.0 μg/mL (MIC) | (Promgool et al., 2022) |
|  |  | Broth dilution/*B. subtilis* | 8.0 μg/mL (MIC) | Kanamycin 2.0 μg/mL (MIC) | (Promgool et al., 2022) |
| Mollicellin F (**95**) | Antibacterial | Broth dilution/*B. cereus* | 4.0 μg/mL (MIC) | Kanamycin 2.0 μg/mL (MIC) | (Promgool et al., 2022) |
|  |  | Broth dilution/*B. subtilis* | 4.0 μg/mL (MIC) | Kanamycin 2.0 μg/mL (MIC) | (Promgool et al., 2022) |
| Mollicellin H (**97**) | Antibacterial | Broth dilution/*S. aureus* ATCC29213 | 5.14 μg/mL (IC_50_) | Streptomycin sulfate 1.05 μg/mL (IC_50_) | (Ouyang et al., 2018) |
|  |  | Broth dilution/*S. aureus* N50 | 6.21 μg/mL (IC_50_) | Streptomycin sulfate ˃100 μg/mL (IC_50_) | (Ouyang et al., 2018) |
|  |  | Broth microdilution/*S. aureus* | 25.0 μg/mL (MIC) | Vancomycin 1.0 μg/mL (MIC) | (Zhao et al., 2021) |
|  |  | Broth microdilution/MRSA | 25.0 μg/mL (MIC) | Vancomycin 1.0 μg/mL (MIC) | (Zhao et al., 2021) |
|  |  | Broth dilution/*B. cereus* | 4.0 μg/mL (MIC) | Kanamycin 2.0 μg/mL (MIC) | (Promgool et al., 2022) |
|  |  | Broth dilution/*B. subtilis* | 8.0 μg/mL (MIC) | Kanamycin 2.0 μg/mL (MIC) | (Promgool et al., 2022) |
| Mollicellin M (**99**) | Antibacterial | Broth dilution/*B. cereus* | 16.0 μg/mL (MIC) | Kanamycin 2.0 μg/mL (MIC) | (Promgool et al., 2022) |
|  |  | Broth dilution/*B. subtilis* | 16.0 μg/mL (MIC) | Kanamycin 2.0 μg/mL (MIC) | (Promgool et al., 2022) |
| Mollicellin N (**100**) | Antibacterial | Broth dilution/*B. cereus* | 8.0 μg/mL (MIC) | Kanamycin 2.0 μg/mL (MIC) | (Promgool et al., 2022) |
|  |  | Broth dilution/*B. subtilis* | 16.0 μg/mL (MIC) | Kanamycin 2.0 μg/mL (MIC) | (Promgool et al., 2022) |
| Mollicellin S (**105**) | Antibacterial | Broth microdilution/*S. aureus* | 6.25 μg/mL (MIC) | Vancomycin 1.0 μg/mL (MIC) | (Zhao et al., 2021) |
|  |  | Broth microdilution/MRSA | 6.25 μg/mL (MIC) | Vancomycin 1.0 μg/mL (MIC) | (Zhao et al., 2021) |
| Mollicellin T (**106**) | Antibacterial | Broth microdilution/*S. aureus* | 12.5 μg/mL (MIC) | Vancomycin 1.0 μg/mL (MIC) | (Zhao et al., 2021) |
|  |  | Broth microdilution/MRSA | 12.5 μg/mL (MIC) | Vancomycin 1.0 μg/mL (MIC) | (Zhao et al., 2021) |
| Mollicellin U (**107**) | Antibacterial | Broth microdilution/*S. aureus* | 12.5 μg/mL (MIC) | Vancomycin 1.0 μg/mL (MIC) | (Zhao et al., 2021) |
|  |  | Broth microdilution/MRSA | 25.0 μg/mL (MIC) | Vancomycin 1.0 μg/mL (MIC) | (Zhao et al., 2021) |
| Mollicellin V (**108**) | Antibacterial | Broth dilution/*B. cereus* | 4.0 μg/mL (MIC) | Kanamycin 2.0 μg/mL (MIC) | (Promgool et al., 2022) |
|  |  | Broth dilution/*B. subtilis* | 8.0 μg/mL (MIC) | Kanamycin 2.0 μg/mL (MIC) | (Promgool et al., 2022) |
| Mollicellin W (**109**) | Antibacterial | Broth dilution/*B. cereus* | 4.0 μg/mL (MIC) | Kanamycin 2.0 μg/mL (MIC) | (Promgool et al., 2022) |
|  |  | Broth dilution/*B. subtilis* | 4.0 μg/mL (MIC) | Kanamycin 2.0 μg/mL (MIC) | (Promgool et al., 2022) |
| Spiromastixone P1 (**122**) | Antibacterial | Broth microdilution/*S. aureus* | 8.0 μg/mL (MIC) | Chloramphenicol 1.0 μg/mL (MIC) | (Niu et al., 2021a) |
|  |  | Broth microdilution/*B. thuringiensis* | 4.0 μg/mL (MIC) | Chloramphenicol 1.0 μg/mL (MIC) | (Niu et al., 2021a) |
|  |  | Broth microdilution/*B. subtilis* | 8.0 μg/mL (MIC) | Chloramphenicol 1.0 μg/mL (MIC) | (Niu et al., 2021a) |
| Spiromastixone Q (**123**) | Antibacterial | Broth microdilution/*S. aureus* | 4.0 μg/mL (MIC) | Chloramphenicol 1.0 μg/mL (MIC) | (Niu et al., 2021a) |
|  |  | Broth microdilution/*B. thuringiensis* | 2.0 μg/mL (MIC) | Chloramphenicol 1.0 μg/mL (MIC) | (Niu et al., 2021a) |
|  |  | Broth microdilution/*B.subtilis* | 2.0 μg/mL (MIC) | Chloramphenicol 1.0 μg/mL (MIC) | (Niu et al., 2021a) |
| Spiromastixone R (**126**) | Antibacterial | Broth microdilution/*S. aureus* | 1.0 μg/mL (MIC) | Chloramphenicol 1.0 μg/mL (MIC) | (Niu et al., 2021a) |
|  |  | Broth microdilution/*B. thuringiensis* | 1.0 μg/mL (MIC) | Chloramphenicol 1.0 μg/mL (MIC) | (Niu et al., 2021a) |
|  |  | Broth microdilution/*B. subtilis* | 0.5 μg/mL (MIC) | Chloramphenicol 1.0 μg/mL (MIC) | (Niu et al., 2021a) |
| Spiromastixone S1 (**128**) | Antibacterial | Broth microdilution/*S. aureus* | 32.0 μg/mL (MIC) | Chloramphenicol 1.0 μg/mL (MIC) | (Niu et al., 2021a) |
|  |  | Broth microdilution/*B. thuringiensis* | 32.0 μg/mL (MIC) | Chloramphenicol 1.0 μg/mL (MIC) | (Niu et al., 2021a) |
|  |  | Broth microdilution/*B. subtilis* | 16.0 μg/mL (MIC) | Chloramphenicol 1.0 μg/mL (MIC) | (Niu et al., 2021a) |
| Unguinol (**138**) | Antibacterial | Broth microdilution/*S. subtilis* | 3.1 μg/mL (MIC) | Ampicillin 0.2 μg/mL (MIC) | (Morshed et al., 2018) |
|  |  | Broth microdilution/*S. aureus* | 12.5 μg/mL (MIC) | Ampicillin 3.1 μg/mL (MIC) | (Morshed et al., 2018) |
| 2-Chlorounguinol (**139**) | Antibacterial | Broth microdilution/*S. subtilis* | 12.5 μg/mL (MIC) | Ampicillin 0.2 μg/mL (MIC) | (Morshed et al., 2018) |
|  |  | Broth microdilution/*S. aureus* | 25.0 μg/mL (MIC) | Ampicillin 3.1 μg/mL (MIC) | (Morshed et al., 2018) |
|  |  | Broth dilution/*S. aureus* | 4.0 μg/mL (MIC) | Vancomycin 0.25 μg/mL (MIC) | (Phainuphong et al., 2018) |
|  |  | Broth microdilution/*S. aureus* | 8.0 μg/mL (MIC) | Vancomycin 0.5 μg/mL (MIC) | (Saetang et al., 2021) |
|  |  | Broth dilution/ MRSA | 8.0 μg/mL (MIC) | Vancomycin 0.5 μg/mL (MIC) | (Phainuphong et al., 2018) |
|  |  | Broth microdilution/MRSA | 8.0 μg/mL (MIC) | Vancomycin 1.0 μg/mL (MIC) | (Saetang et al., 2021) |
|  |  | Broth microdilution*/B. cereus* | 3.13 μg/mL (MIC) | Vancomycin 2.0 μg/mL (MIC) | (Sadorn et al., 2022) |
|  | Antifungal | Broth microdilution/*S. cerevisiae* | 25.0 μg/mL (MIC) | Clotrimazole 0.4 μg/mL (MIC) | (Morshed et al., 2018) |
|  |  | Broth dilution/ *C. albicans* | 8.0 μg/mL (MIC) | Amphotericin B 0.25 μg/mL (MIC) | (Phainuphong et al., 2018) |
|  |  | Broth dilution/ *C. gypseum* | 8.0 μg/mL (MIC) | Miconazol 1.0 μg/mL (MIC) | (Phainuphong et al., 2018) |
|  |  | Broth microdilution/*C. neoformans* | 32.0 μg/mL (MIC) | Amphotericin B 2.0 μg/mL (MIC) | (Saetang et al., 2021) |
|  |  | Broth microdilution/*C. albicans* | 8.0 μg/mL (MIC) | Amphotericin B 0.5 μg/mL (MIC) | (Saetang et al., 2021) |
|  |  | Broth microdilution/*M. gypseum* | 8.0 μg/mL (MIC) | Clotrimazole 2.0 μg/mL (MIC) | (Saetang et al., 2021) |
|  |  | Broth microdilution/*C. acutatum* | 3.13 μg/mL (MIC) | Amphotericin B 1.56 μg/mL (MIC) | (Sadorn et al., 2022) |
| 2,4-Dichlorounguinol (**140**) | Antibacterial | Broth dilution/*S. aureus* | 2.0 μg/mL (MIC) | Vancomycin 0.25 μg/mL (MIC) | (Phainuphong et al., 2018) |
|  |  | Broth dilution/MRSA | 2.0 μg/mL (MIC) | Vancomycin 0.5 μg/mL (MIC) | (Phainuphong et al., 2018) |
|  | Antifungal | Broth dilution/*C. neoformans* | 8.0 μg/mL (MIC) | Amphotericin B 0.25 μg/mL (MIC) | (Phainuphong et al., 2018) |
| 4,7-Dichlorounguinol (**142**) | Antibacterial | Broth microdilution/*S. subtilis* | 3.1 μg/mL (MIC) | Ampicillin 0.2 μg/mL (MIC) | (Morshed et al., 2018) |
|  |  | Broth microdilution/*S. aureus* | 6.3 μg/mL (MIC) | Ampicillin 3.1 μg/mL (MIC) | (Morshed et al., 2018) |
|  |  | Broth microdilution*/B. cereus* | 6.25 μg/mL (MIC) | Vancomycin 2.0 μg/mL (MIC) | (Sadorn et al., 2022) |
|  | Antifungal | Broth microdilution/*S. cerevisiae* | 6.3 μg/mL (MIC) | Clotrimazole 0.4 μg/mL (MIC) | (Morshed et al., 2018) |
|  |  | Broth microdilution*/B. cereus* | 25.0 μg/mL (MIC) | Vancomycin 2.0 μg/mL (MIC) | (Sadorn et al., 2022) |
| 2-Chloro-7-bromounguinol (**144**) | Antibacterial | Broth microdilution/*S. subtilis* | 2.6 μg/mL (MIC) | Ampicillin 0.2 μg/mL (MIC) | (Morshed et al., 2018) |
|  |  | Broth microdilution/*S. aureus* | 2.6 μg/mL (MIC) | Ampicillin 3.1 μg/mL (MIC) | (Morshed et al., 2018) |
|  | Antifungal | Broth microdilution/*S. cerevisiae* | 2.6 μg/mL (MIC) | Clotrimazole 0.4 μg/mL (MIC) | (Morshed et al., 2018) |
| 7-Bromounguinol (**145**) | Antibacterial | Broth microdilution/*S. subtilis* | 3.1 μg/mL (MIC) | Ampicillin 0.2 μg/mL (MIC) | (Morshed et al., 2018) |
|  |  | Broth microdilution/*S. aureus* | 6.3 μg/mL (MIC) | Ampicillin 3.1 μg/mL (MIC) | (Morshed et al., 2018) |
|  | Antifungal | Broth microdilution/*S. cerevisiae* | 6.3 μg/mL (MIC) | Clotrimazole 0.4 μg/mL (MIC) | (Morshed et al., 2018) |
| Nidulin (**146**) | Antibacterial | Broth microdilution/*S. subtilis* | 0.8 μg/mL (MIC) | Ampicillin 0.2 μg/mL (MIC) | (Morshed et al., 2018) |
|  |  | Broth microdilution/*S. aureus* | 6.3 μg/mL (MIC) | Ampicillin 3.1 μg/mL (MIC) | (Morshed et al., 2018) |
|  |  | Broth dilution/*S. aureus* | 4.0 μg/mL (MIC) | Vancomycin 0.25 μg/mL (MIC) | (Phainuphong et al., 2018) |
|  |  | Broth dilution/MR *S. aureus* | 8.0 μg/mL (MIC) | Vancomycin 0.5 μg/mL (MIC) | (Phainuphong et al., 2018) |
|  |  | Broth microdilution/*S. aureus* | 4.0 μg/mL (MIC) | Vancomycin 0.5 μg/mL (MIC) | (Saetang et al., 2021) |
|  |  | Broth microdilution/MRSA | 8.0 μg/mL (MIC) | Vancomycin 1.0 μg/mL (MIC) | (Saetang et al., 2021) |
|  |  | Broth microdilution*/B. cereus* | 1.56 μg/mL (MIC) | Vancomycin 2.0 μg/mL (MIC) | (Sadorn et al., 2022) |
|  | Antifungal | Broth dilution/ *C. neoformans* | 16.0 μg/mL (MIC) | Amphotericin B 0.25 μg/mL (MIC) | (Phainuphong et al., 2018) |
|  |  | Broth microdilution/*C. neoformans* | 16.0 μg/mL (MIC) | Amphotericin B 2.0 μg/mL (MIC) | (Saetang et al., 2021) |
|  |  | Broth microdilution/*C. acutatum* | 6.25 μg/mL (MIC) | Amphotericin B 1.56 μg/mL (MIC) | (Sadorn et al., 2022) |
| Nornidulin (**147**) | Antibacterial | Broth microdilution/*S. subtilis* | 1.6 μg/mL (MIC) | Ampicillin 0.2 μg/mL (MIC) | (Morshed et al., 2018) |
|  |  | Broth microdilution/*S. aureus* | 6.3 μg/mL (MIC) | Ampicillin 3.1 μg/mL (MIC) | (Morshed et al., 2018) |
|  |  | Broth dilution/*S. aureus* | 2.0 μg/mL (MIC) | Vancomycin 0.25 μg/mL (MIC) | (Phainuphong et al., 2018) |
|  |  | Broth dilution/MR *S. aureus* | 2.0 μg/mL (MIC) | Vancomycin 0.5 μg/mL (MIC) | (Phainuphong et al., 2018) |
|  |  | Broth microdilution/*S. aureus* | 2.0 μg/mL (MIC) | Vancomycin 0.5 μg/mL (MIC) | (Saetang et al., 2021) |
|  |  | Broth microdilution/MRSA | 1.0 μg/mL (MIC) | Vancomycin 1.0 μg/mL (MIC) | (Saetang et al., 2021) |
|  |  | Broth microdilution/MRSA | 2.0 μg/mL (MIC) | Ampicillin 1.0 μg/mL (MIC) | (Zhang et al., 2022) |
|  |  | Broth microdilution/*M. variabilis* | 8.0 μg/mL (MIC) | Nalidixic acid 16.0 μg/mL (MIC) | (Zhang et al., 2022) |
|  |  | Broth microdilution/*M. jannaschii* | 16.0 μg/mL (MIC) | Nalidixic acid 16.0 μg/mL (MIC) | (Zhang et al., 2022) |
|  |  | Broth microdilution*/B. cereus* | 3.13 μg/mL (MIC) | Vancomycin 2.0 μg/mL (MIC) | (Sadorn et al., 2022) |
|  | Antifungal | Broth dilution/ *C. neoformans* | 8.0 μg/mL (MIC) | Amphotericin B 0.25 μg/mL (MIC) | (Phainuphong et al., 2018) |
|  |  | Broth microdilution/*C. neoformans* | 8.0 μg/mL (MIC) | Amphotericin B 2.0 μg/mL (MIC) | (Saetang et al., 2021) |
|  |  | Broth microdilution/*C. acutatum* | 3.13 μg/mL (MIC) | Amphotericin B 1.56 μg/mL (MIC) | (Sadorn et al., 2022) |
| Aspergillusidone A (**148**) | Antibacterial | Broth microdilution/MRSA | 12.8 μM (MIC) | Ampicillin 1.6 μM (MIC) | (Yang et al., 2018) |
|  | Antifungal | Broth microdilution/*C. albicans* | 6.4 μM (MIC) | Ketoconazole 1.9 μM (MIC) | (Yang et al., 2018) |
| Aspergillusidone B (**149**) | Antibacterial | Broth microdilution*/B. cereus* | 3.13 μg/mL (MIC) | Vancomycin 2.0 μg/mL (MIC) | (Sadorn et al., 2022) |
| Aspergillusidone C (**150**) | Antibacterial | Broth microdilution/*S. subtilis* | 3.1 μg/mL (MIC) | Ampicillin 0.2 μg/mL (MIC) | (Morshed et al., 2018) |
|  |  | Broth microdilution/*S. aureus* | 6.3 μg/mL (MIC) | Ampicillin 3.1 μg/mL (MIC) | (Morshed et al., 2018) |
|  |  | Broth dilution/*S. aureus* | 2.0 μg/mL (MIC) | Vancomycin 0.25 μg/mL (MIC) | (Phainuphong et al., 2018) |
|  |  | Broth dilution/MR *S. aureus* | 1.0 μg/mL (MIC) | Vancomycin 0.5 μg/mL (MIC) | (Phainuphong et al., 2018) |
|  |  | Broth microdilution/*S. aureus* | 2.0 μg/mL (MIC) | Vancomycin 0.5 μg/mL (MIC) | (Saetang et al., 2021) |
|  |  | Broth microdilution/MR *S. aureus* | 1.0 μg/mL (MIC) | Vancomycin 1.0 μg/mL (MIC) | (Saetang et al., 2021) |
|  |  | Broth microdilution/MR *S. aureus* | 32.0 μg/mL (MIC) | Ampicillin 1.0 μg/mL (MIC) | (Zhang et al., 2022) |
|  |  | Broth microdilution/*M. variabilis* | 8.0 μg/mL (MIC) | Nalidixic acid 16.0 μg/mL (MIC) | (Zhang et al., 2022) |
|  |  | Broth microdilution/*M. jannaschii* | 32.0 μg/mL (MIC) | Nalidixic acid 16.0 μg/mL (MIC) | (Zhang et al., 2022) |
|  |  | Broth microdilution*/B. cereus* | 3.13 μg/mL (MIC) | Vancomycin 2.0 μg/mL (MIC) | (Sadorn et al., 2022) |
|  | Antifungal | Broth microdilution/*S. cerevisiae* | 6.3 μg/mL (MIC) | Clotrimazole 0.4 μg/mL (MIC) | (Morshed et al., 2018) |
|  |  | Broth dilution/*M. gypseum* | 2.0 μg/mL (MIC) | Miconazol 1.0 μg/mL (MIC) | (Phainuphong et al., 2018) |
|  |  | Broth microdilution/*M. gypseum* | 2.0 μg/mL (MIC) | Clotrimazole 2.0 μg/mL (MIC) | (Saetang et al., 2021) |
|  |  | Broth microdilution/*A. brassicicola* | 6.25 μg/mL (MIC) | Amphotericin B 1.56 μg/mL (MIC) | (Sadorn et al., 2022) |
|  |  | Broth microdilution*/B. cereus* | 3.13 μg/mL (MIC) | Vancomycin 2.0 μg/mL (MIC) | (Sadorn et al., 2022) |
| Aspergillusidone D (**151**) | Antibacterial | Broth microdilution/*S. subtilis* | 2.9 μg/mL (MIC) | Ampicillin 0.2 μg/mL (MIC) | (Morshed et al., 2018) |
|  |  | Broth microdilution/*S. aureus* | 2.9 μg/mL (MIC) | Ampicillin 3.1 μg/mL (MIC) | (Morshed et al., 2018) |
|  | Antifungal | Broth microdilution/*S. cerevisiae* | 2.9 μg/mL (MIC) | Clotrimazole 0.4 μg/mL (MIC) | (Morshed et al., 2018) |
| Aspergillusidone E (**152**) | Antibacterial | Broth microdilution/*S. subtilis* | 3.1 μg/mL (MIC) | Ampicillin 0.2 μg/mL (MIC) | (Morshed et al., 2018) |
|  |  | Broth microdilution/*S. aureus* | 6.3 μg/mL (MIC) | Ampicillin 3.1 μg/mL (MIC) | (Morshed et al., 2018) |
|  | Antifungal | Broth microdilution/*S. cerevisiae* | 6.3 μg/mL (MIC) | Clotrimazole 0.4 μg/mL (MIC) | (Morshed et al., 2018) |
| Aspergillusidone F (**153**) | Antibacterial | Broth microdilution/*S. subtilis* | 2.9 μg/mL (MIC) | Ampicillin 0.2 μg/mL (MIC) | (Morshed et al., 2018) |
|  |  | Broth microdilution/*S. aureus* | 5.8 μg/mL (MIC) | Ampicillin 3.1 μg/mL (MIC) | (Morshed et al., 2018) |
|  |  | Broth microdilution/*P. aeruginosa* | 6.4 μM (MIC) | Ampicillin 1.6 μM (MIC) | (Yang, W. et al., 2018) |
|  |  | Broth microdilution/MRSA | 25.6 μM (MIC) | Ampicillin 6.4 μM (MIC) | (Yang, W. et al., 2018) |
|  | Antifungal | Broth microdilution/*S. cerevisiae* | 2.9 μg/mL (MIC) | Clotrimazole 0.4 μg/mL (MIC) | (Morshed et al., 2018) |
| Aspersidone (**155**) | Antibacterial | Broth dilution/*S. aureus* | 0.5 μg/mL (MIC) | Vancomycin 0.25 μg/mL (MIC) | (Phainuphong et al., 2018) |
|  |  | Broth dilution/MR *S. aureus* | 0.5 μg/mL (MIC) | Vancomycin 0.5 μg/mL (MIC) | (Phainuphong et al., 2018) |
| Aspersidone B (**156**) | Antibacterial | Broth dilution/*B. subtilis* | 10.7 μM (MIC) | Kanamycin 1.0 μM (MIC) | (Anh et al., 2022) |
|  |  | Broth dilution/*Micrococcus luteus* | 10.7 μM (MIC) | Kanamycin 0.5 μM (MIC) | (Anh et al., 2022) |
|  |  | Broth dilution/*S. aureus* | 5.3 μM (MIC) | Kanamycin 1.0 μM (MIC) | (Anh et al., 2022) |
| Folipastatin (**157**) | Antibacterial | Broth microdilution/*S. subtilis* | 0.8 μg/mL (MIC) | Ampicillin 0.2 μg/mL (MIC) | (Morshed et al., 2018) |
|  |  | Broth microdilution/*S. aureus* | 1.6 μg/mL (MIC) | Ampicillin 3.1 μg/mL (MIC) | (Morshed et al., 2018) |
|  |  | Broth dilution/*S. aureus* | 2.0 μg/mL (MIC) | Vancomycin 0.25 μg/mL (MIC) | (Phainuphong et al., 2018) |
|  |  | Broth dilution/MR *S. aureus* | 1.0 μg/mL (MIC) | Vancomycin 0.5 μg/mL (MIC) | (Phainuphong et al., 2018) |
|  |  | Broth microdilution/*S. aureus* | 2.0 μg/mL (MIC) | Vancomycin 0.5 μg/mL (MIC) | (Saetang et al., 2021) |
|  |  | Broth microdilution/MR *S. aureus* | 1.0 μg/mL (MIC) | Vancomycin 1.0 μg/mL (MIC) | (Saetang et al., 2021) |
|  | Antifungal | Broth dilution/ *C. neoformans* | 1.0 μg/mL (MIC) | Amphotericin B 0.25 μg/mL (MIC) | (Phainuphong et al., 2018) |
|  |  | Broth microdilution/*C. neoformans* | 1.0 μg/mL (MIC) | Amphotericin B 2.0 μg/mL (MIC) | (Saetang et al., 2021) |
| 7-Carboxyfolipastatin (**158**) | Antibacterial | Broth microdilution/*S. subtilis* | 20.6 μg/mL (MIC) | Ampicillin 0.2 μg/mL (MIC) | (Morshed et al., 2018) |
|  |  | Broth microdilution/*S. aureus* | 41.1 μg/mL (MIC) | Ampicillin 3.1 μg/mL (MIC) | (Morshed et al., 2018) |
| 7-Bromofolipastatin (**159**) | Antibacterial | Broth microdilution/*S. subtilis* | 1.6 μg/mL (MIC) | Ampicillin 0.2 μg/mL (MIC) | (Morshed et al., 2018) |
|  |  | Broth microdilution/*S. aureus* | 3.1 μg/mL (MIC) | Ampicillin 3.1 μg/mL (MIC) | (Morshed et al., 2018) |
| Emeguisin A (**160**) | Antibacterial | Broth microdilution/*S. subtilis* | 0.8 μg/mL (MIC) | Ampicillin 0.2 μg/mL (MIC) | (Morshed et al., 2018) |
|  |  | Broth microdilution/*S. aureus* | 1.6 μg/mL (MIC) | Ampicillin 3.1 μg/mL (MIC) | (Morshed et al., 2018) |
|  |  | Broth dilution/*S. aureus* | 0.5 μg/mL (MIC) | Vancomycin 0.25 μg/mL (MIC) | (Phainuphong et al., 2018) |
|  |  | Broth dilution/MR *S. aureus* | 0.5 μg/mL (MIC) | Vancomycin 0.5 μg/mL (MIC) | (Phainuphong et al., 2018) |
|  |  | Broth microdilution/*S. aureus* | 0.5 μg/mL (MIC) | Vancomycin 0.5 μg/mL (MIC) | (Saetang et al., 2021) |
|  |  | Broth microdilution/MR *S. aureus* | 0.5 μg/mL (MIC) | Vancomycin 1.0 μg/mL (MIC) | (Saetang et al., 2021) |
|  |  | Broth microdilution*/B. cereus* | 3.13 μg/mL (MIC) | Vancomycin 2.0 μg/mL (MIC) | (Sadorn et al., 2022) |
|  | Antifungal | Broth microdilution/*S. cerevisiae* | 6.3 μg/mL (MIC) | Clotrimazole 0.4 μg/mL (MIC) | (Morshed et al., 2018) |
|  |  | Broth dilution/ *C. neoformans* | 0.5 μg/mL (MIC) | Amphotericin B 0.25 μg/mL (MIC) | (Phainuphong et al., 2018) |
|  |  | Broth microdilution/*C. neoformans* | 0.5 μg/mL (MIC) | Amphotericin B 2.0 μg/mL (MIC) | (Saetang et al., 2021) |
|  |  | Broth microdilution/*C. acutatum* | 3.13 μg/mL (MIC) | Amphotericin B 1.56 μg/mL (MIC) | (Sadorn et al., 2022) |
| Emeguisin C (**162**) | Antibacterial | Broth microdilution*/B. cereus* | 1.56 μg/mL (MIC) | Vancomycin 2.0 μg/mL (MIC) | (Sadorn et al., 2022) |
| Emeguisin D (**163**) | Antibacterial | Broth microdilution*/B. cereus* | 1.56 μg/mL (MIC) | Vancomycin 2.0 μg/mL (MIC) | (Sadorn et al., 2022) |
|  |  | Broth microdilution/*S. aureus* | 1.56 μg/mL (MIC) | Vancomycin 1.0 μg/mL (MIC) | (Sadorn et al., 2022) |

**Table S3.** Reported antimalarial and antituberculosis activities results of naturally occurring depsidones.

| **Compound name** | **Microorganism/Assay** | **Biological Results** | | **Ref.** |
| --- | --- | --- | --- | --- |
|  |  | **Compound** | **Positive Control** |  |
| Aspergillusidone B (**149**) | *Plasmodium falciparum/* Microculture radioisotope | 9.02 μM (IC_50_) | Chloroquine 0.51 μM (IC_50_) | (Sadorn et al., 2022) |
| Folipastatin (**157**) | *Plasmodium falciparum/* Microculture radioisotope | 22.29 μM (IC_50_) | Chloroquine 0.51 μM (IC_50_) | (Sadorn et al., 2022) |
| Emeguisin A (**160**) | *Plasmodium falciparum/* Microculture radioisotope | 7.69 μM (IC_50_) | Chloroquine 0.51 μM (IC_50_) | (Sadorn et al., 2022) |
| Emeguisin A (**160**) | *Mycobacterium tuberculosis* H37Ra/GFPMA | 25.0 μg/mL (MIC) | Isoniazid 0.05 μg/mL (MIC) | (Sadorn et al., 2022) |
| Emeguisin B (**161**) | *Plasmodium falciparum/* Microculture radioisotope | 7.95 μM (IC_50_) | Chloroquine 0.51 μM (IC_50_) | (Sadorn et al., 2022) |
| Emeguisin C (**162**) | *Plasmodium falciparum/* Microculture radioisotope | 7.88 μM (IC_50_) | Chloroquine 0.51 μM (IC_50_) | (Sadorn et al., 2022) |
| Emeguisin D (**163**) | *Plasmodium falciparum/* Microculture radioisotope | 8.55 μM (IC_50_) | Chloroquine 0.51 μM (IC_50_) | (Sadorn et al., 2022) |
| Emeguisin D (**163**) | *Mycobacterium tuberculosis* H37Ra/GFPMA | 25.0 μg/mL (MIC) | Isoniazid 0.05 μg/mL (MIC) | (Sadorn et al., 2022) |
| Aspergisidone (**164**) | *Plasmodium falciparum/* Microculture radioisotope | 8.28 μM (IC_50_) | Chloroquine 0.51 μM (IC_50_) | (Sadorn et al., 2022) |

**Table S4.** Reported cytotoxic activity results of naturally occurring depsidones.

| **Compound name** | **Assay/ Cell Line** | **Biological Results** | | **Ref.** |
| --- | --- | --- | --- | --- |
|  |  | **Compound** | **Positive Control** |  |
| 1H-Dibenzo[b,e][1,4]dioxepin-11-one,3,8-dihydroxy-4-(methoxymethyl)-1,6-dimethyl (**18**) | MTT/L5178Y | 7.3 μM (IC_50_) | Kahalalide F 4.30 μM (IC_50_) | (Umeokoli et al., 2019) |
| Curdepsidone A (**29**) | MTT/BEL7402 | 9.85 μM (IC_50_) | 5-Fluorouracil 14.0 μM (IC_50_) | (An et al., 2018) |
|  | MTT/BEL7402 | 2.46 μM (IC_50_) | 5-Fluorouracil 1630 μM (IC_50_) | (An et al., 2018) |
| Mollicellin F (**95**) | MTT/KB | 4.79 μM (IC_50_) | Doxorubicin 0.073 μM (IC_50_) | (Promgool et al., 2022) |
|  | MTT/HepG2 | 7.10 μM (IC_50_) | Doxorubicin 1.89 μM (IC_50_) | (Promgool et al., 2022) |
|  | MTT/Vero | 6.27 μM (IC_50_) | Doxorubicin 1.42 μM (IC_50_) | (Promgool et al., 2022) |
| Mollicellin G (**96**) | MTT/HepG2 | 19.64 μg/mL (IC_50_) | Camptothecin 3.6 μg/mL (IC_50_) | (Ouyang et al., 2018) |
|  | MTT/HeLa | 13.97 μg/mL (IC_50_) | Camptothecin 6.3 μg/mL (IC_50_) | (Ouyang et al., 2018) |
| Mollicellin H (**97**) | MTT/HepG2 | 6.83 μg/mL (IC_50_) | Camptothecin 3.6 μg/mL (IC_50_) | (Ouyang et al., 2018) |
| Mollicellin I (**98**) | MTT/HeLa | 21.35 μg/mL (IC_50_) | Camptothecin 6.3 μg/mL (IC_50_) | (Ouyang et al., 2018) |
| Mollicellin X (**110**) | MTT/KB | 9.83 μM (IC_50_) | Doxorubicin 0.073 μM (IC_50_) | (Promgool et al., 2022) |
|  | MTT/HepG2 | 11.69 μM (IC_50_) | Doxorubicin 1.89 μM (IC_50_) | (Promgool et al., 2022) |
|  | MTT/Vero | 5.65 μM (IC_50_) | Doxorubicin 1.42 μM (IC_50_) | (Promgool et al., 2022) |
| Auranticin A (**112**) | CCK-8/HeLa | 42.2 μM (IC_50_) | Cisplatin 11.7 μM (IC_50_) | (Zhao et al., 2020) |
|  | CCK-8/A549 | 33.7 μM (IC_50_) | Cisplatin 11.8 μM (IC_50_) | (Zhao et al., 2020) |
|  | CCK-8/HepG-2 | 42.2 μM (IC_50_) | Cisplatin 9.3 μM (IC_50_) | (Zhao et al., 2020) |
|  | CCK-8/HL-60 | 22.8 μM (IC_50_) | Cisplatin 15.7 μM (IC_50_) | (Zhao et al., 2020) |
| Auranticin B (**113**) | CCK-8/PC-3 | 21.8 μM (IC_50_) | Cisplatin 5.6 μM (IC_50_) | (Zhao et al., 2020) |
|  | CCK-8/A549 | 13.0 μM (IC_50_) | Cisplatin 11.8 μM (IC_50_) | (Zhao et al., 2020) |
|  | CCK-8/HepG-2 | 61.2 μM (IC_50_) | Cisplatin 9.3 μM (IC_50_) | (Zhao et al., 2020) |
|  | CCK-8/HL-60 | 21.9 μM (IC_50_) | Cisplatin 15.7 μM (IC_50_) | (Zhao et al., 2020) |
| Pilobolusone C (**114**) | CCK-8/PC-3 | 23.4 μM (IC_50_) | Cisplatin 5.6 μM (IC_50_) | (Zhao et al., 2020) |
|  | CCK-8/A549 | 66.9 μM (IC_50_) | Cisplatin 11.8 μM (IC_50_) | (Zhao et al., 2020) |
|  | CCK-8/HepG-2 | 86.8 μM (IC_50_) | Cisplatin 9.3 μM (IC_50_) | (Zhao et al., 2020) |
| Himantormione A (**130**) | MTS/HCT-116 | 1.11 μM (EC_50_) | 5-Fluouracil 9.4 μM (EC_50_) | (Hyung Koo et al., 2022) |
| Unguinol (**138**) | MTT/NS-1 | 12.5 μg/mL (MIC) | 5-Fluorouracil 0.1 μg/mL (MIC) | (Morshed et al., 2018) |
|  | SRB/ACHN | 5.0 μM (IC_50_) | Adriamycin 0.15 μM (IC_50_) | (Anh et al., 2022) |
|  | SRB/NCI-H23 | 4.4 μM (IC_50_) | Adriamycin 0.12 μM (IC_50_) | (Anh et al., 2022) |
|  | SRB/PC-3 | 4.4 μM (IC_50_) | Adriamycin 0.15 μM (IC_50_) | (Anh et al., 2022) |
|  | SRB/NUGC-3 | 3.4 μM (IC_50_) | Adriamycin 0.15 μM (IC_50_) | (Anh et al., 2022) |
|  | SRB/MDA-MB-231 | 6.0 μM (IC_50_) | Adriamycin 0.16 μM (IC_50_) | (Anh et al., 2022) |
|  | SRB/HCT-15 | 6.2 μM (IC_50_) | Adriamycin 0.15 μM (IC_50_) | (Anh et al., 2022) |
| 2-Chlorounguinol (**139**) | MTT/NS-1 | 25.0 μg/mL (MIC) | 5-Fluorouracil 0.1 μg/mL (MIC) | (Morshed et al., 2018) |
|  | MTT/Vero | 86.0 μM (IC_50_) | Ellipticine 4.0 μM (IC_50_) | (Saetang et al., 2021) |
|  | SRB/ACHN | 4.6 μM (IC_50_) | Adriamycin 0.15 μM (IC_50_) | (Anh et al., 2022) |
|  | SRB/NCI-H23 | 4.0 μM (IC_50_) | Adriamycin 0.12 μM (IC_50_) | (Anh et al., 2022) |
|  | SRB/PC-3 | 3.7 μM (IC_50_) | Adriamycin 0.15 μM (IC_50_) | (Anh et al., 2022) |
|  | SRB/NUGC-3 | 3.4 μM (IC_50_) | Adriamycin 0.15 μM (IC_50_) | (Anh et al., 2022) |
|  | SRB/MDA-MB-231 | 4.6 μM (IC_50_) | Adriamycin 0.16 μM (IC_50_) | (Anh et al., 2022) |
|  | SRB/HCT-15 | 3.9 μM (IC_50_) | Adriamycin 0.15 μM (IC_50_) | (Anh et al., 2022) |
|  | Brine Shrimp/*Artemia salina* larva | 75.9 μM (LC_50_) | Hg(NO3)_2_ 77.0 μM (LC_50_) | (Yang, W. et al., 2018) |
| 4,7-Dichlorounguinol (**142**) | MTT/NS-1 | 50.0 μg/mL (MIC) | 5-Fluorouracil 0.1 μg/mL (MIC) | (Morshed et al., 2018) |
| 3,1*`*-Dichlorounguinol (**143**) | SRB/ACHN | 4.8 μM (IC_50_) | Adriamycin 0.15 μM (IC_50_) | (Anh et al., 2022) |
|  | SRB/NCI-H23 | 4.7 μM (IC_50_) | Adriamycin 0.12 μM (IC_50_) | (Anh et al., 2022) |
|  | SRB/PC-3 | 4.8 μM (IC_50_) | Adriamycin 0.15 μM (IC_50_) | (Anh et al., 2022) |
|  | SRB/NUGC-3 | 3.8 μM (IC_50_) | Adriamycin 0.15 μM (IC_50_) | (Anh et al., 2022) |
|  | SRB/MDA-MB-231 | 5.5 μM (IC_50_) | Adriamycin 0.16 μM (IC_50_) | (Anh et al., 2022) |
|  | SRB/HCT-15 | 5.2 μM (IC_50_) | Adriamycin 0.15 μM (IC_50_) | (Anh et al., 2022) |
| 2-Chloro-7-bromounguinol (**144**) | MTT/NS-1 | 42.2 μg/mL (MIC) | 5-Fluorouracil 0.1 μg/mL (MIC) | (Morshed et al., 2018) |
| 7-Bromounguinol (**145**) | MTT/NS-1 | 50.0 μg/mL (MIC) | 5-Fluorouracil 0.1 μg/mL (MIC) | (Morshed et al., 2018) |
| Nidulin (**146**) | MTT/NS-1 | 27.2 μg/mL (MIC) | 5-Fluorouracil 0.1 μg/mL (MIC) | (Morshed et al., 2018) |
|  | MTT/Vero | 57.36 μM (IC_50_) | Ellipticine 4.55 μM (IC_50_) | (Phainuphong et al., 2018) |
|  | MTT/Vero | 57.0 μM (IC_50_) | Ellipticine 4.0 μM (IC_50_) | (Saetang et al., 2021) |
|  | SRB/ACHN | 27.7 μM (IC_50_) | Adriamycin 0.15 μM (IC_50_) | (Anh et al., 2022) |
|  | SRB/NCI-H23 | 16.1 μM (IC_50_) | Adriamycin 0.12 μM (IC_50_) | (Anh et al., 2022) |
|  | SRB/PC-3 | 26.6 μM (IC_50_) | Adriamycin 0.15 μM (IC_50_) | (Anh et al., 2022) |
|  | SRB/NUGC-3 | 18.9 μM (IC_50_) | Adriamycin 0.15 μM (IC_50_) | (Anh et al., 2022) |
|  | SRB/MDA-MB-231 | 24.3 μM (IC_50_) | Adriamycin 0.16 μM (IC_50_) | (Anh et al., 2022) |
|  | SRB/HCT-15 | 24.6 μM (IC_50_) | Adriamycin 0.15 μM (IC_50_) | (Anh et al., 2022) |
|  | Brine Shrimp/*Artemia salina* larva | 4.5 μM (LC_50_) | Hg(NO3)_2_ 77.0 μM (LC_50_) | (Yang et al., 2018) |
| Nornidulin (**147**) | SRB/ACHN | 7.3 μM (IC_50_) | Adriamycin 0.15 μM (IC_50_) | (Anh et al., 2022) |
|  | SRB/NCI-H23 | 9.8 μM (IC_50_) | Adriamycin 0.12 μM (IC_50_) | (Anh et al., 2022) |
|  | SRB/PC-3 | 7.5 μM (IC_50_) | Adriamycin 0.15 μM (IC_50_) | (Anh et al., 2022) |
|  | SRB/NUGC-3 | 4.3 μM (IC_50_) | Adriamycin 0.15 μM (IC_50_) | (Anh et al., 2022) |
|  | SRB/MDA-MB-231 | 13.3 μM (IC_50_) | Adriamycin 0.16 μM (IC_50_) | (Anh et al., 2022) |
|  | SRB/HCT-15 | 10.5 μM (IC_50_) | Adriamycin 0.15 μM (IC_50_) | (Anh et al., 2022) |
|  | Brine Shrimp/*Artemia salina* larva | 12.8 μM (LC_50_) | Hg(NO3)_2_ 77.0 μM (LC_50_) | (Yang et al., 2018) |
| Aspergillusidone A (**148**) | Brine Shrimp/*Artemia salina* larva | 51.2 μM (LC_50_) | Hg(NO3)_2_ 77.0 μM (LC_50_) | (Yang et al., 2018) |
| Aspergillusidone C (**150**) | MTT/NS-1 | 25.0 μg/mL (MIC) | 5-Fluorouracil 0.1 μg/mL (MIC) | (Morshed et al., 2018) |
|  | MTT/KB | 17.23 μM (IC_50_) | Ellipticine 10.56 μM (IC_50_) | (Phainuphong et al., 2018) |
|  | MTT/MCF-7 | 30.41 μM (IC_50_) | Doxorubicin 16.83 μM (IC_50_) | (Phainuphong et al., 2018) |
|  | MTT/Vero | 8.88 μM (IC_50_) | Ellipticine 4.55 μM (IC_50_) | (Phainuphong et al., 2018) |
|  | MTT/Vero | 8.9.0 μM (IC_50_) | Ellipticine 4.0 μM (IC_50_) | (Saetang et al., 2021) |
|  | Brine Shrimp/*Artemia salina* larva | 72.0 μM (LC_50_) | Hg(NO3)_2_ 77.0 μM (LC_50_) | (Yang, W. et al., 2018) |
| Aspergillusidone D (**151**) | MTT/NS-1 | 46.7 μg/mL (MIC) | 5-Fluorouracil 0.1 μg/mL (MIC) | (Morshed et al., 2018) |
| Aspergillusidone E (**152**) | MTT/NS-1 | 25.0 μg/mL (MIC) | 5-Fluorouracil 0.1 μg/mL (MIC) | (Morshed et al., 2018) |
| Aspergillusidone F (**153**) | Brine Shrimp/*Artemia salina* larva | 12.8 μM (LC_50_) | Hg(NO3)_2_ 77.0 μM (LC_50_) | (Yang et al., 2018) |
|  | MTT/NS-1 | 46.6 μg/mL (MIC) | 5-Fluorouracil 0.1 μg/mL (MIC) | (Morshed et al., 2018) |
| Aspersidone (**155**) | MTT/Vero | 16.69 μM (IC_50_) | Ellipticine 4.55 μM (IC_50_) | (Phainuphong et al., 2018) |
|  | SRB/ACHN | 11.8 μM (IC_50_) | Adriamycin 0.15 μM (IC_50_) | (Anh et al., 2022) |
|  | SRB/NCI-H23 | 11.9 μM (IC_50_) | Adriamycin 0.12 μM (IC_50_) | (Anh et al., 2022) |
|  | SRB/PC-3 | 10.2 μM (IC_50_) | Adriamycin 0.15 μM (IC_50_) | (Anh et al., 2022) |
|  | SRB/NUGC-3 | 7.7 μM (IC_50_) | Adriamycin 0.15 μM (IC_50_) | (Anh et al., 2022) |
|  | SRB/MDA-MB-231 | 7.8 μM (IC_50_) | Adriamycin 0.16 μM (IC_50_) | (Anh et al., 2022) |
|  | SRB/HCT-15 | 9.1 μM (IC_50_) | Adriamycin 0.15 μM (IC_50_) | (Anh et al., 2022) |
| Aspersidone B (**156**) | SRB/ACHN | 12.9 μM (IC_50_) | Adriamycin 0.15 μM (IC_50_) | (Anh et al., 2022) |
|  | SRB/NCI-H23 | 14.0 μM (IC_50_) | Adriamycin 0.12 μM (IC_50_) | (Anh et al., 2022) |
|  | SRB/PC-3 | 14.4 μM (IC_50_) | Adriamycin 0.15 μM (IC_50_) | (Anh et al., 2022) |
|  | SRB/NUGC-3 | 8.3 μM (IC_50_) | Adriamycin 0.15 μM (IC_50_) | (Anh et al., 2022) |
|  | SRB/MDA-MB-231 | 15.5 μM (IC_50_) | Adriamycin 0.16 μM (IC_50_) | (Anh et al., 2022) |
|  | SRB/HCT-15 | 12.5 μM (IC_50_) | Adriamycin 0.15 μM (IC_50_) | (Anh et al., 2022) |
| Folipastatin (**157**) | MTT/NS-1 | 6.3 μg/mL (MIC) | 5-Fluorouracil 0.1 μg/mL (MIC) | (Morshed et al., 2018) |
|  | MTT/KB | 47.21 μM (IC_50_) | Ellipticine 10.56 μM (IC_50_) | (Phainuphong et al., 2018) |
|  | MTT/MCF-7 | 65.48 μM (IC_50_) | Doxorubicin 16.83 μM (IC_50_) | (Phainuphong et al., 2018) |
|  | MTT/Vero | 17.27 μM (IC_50_) | Ellipticine 4.55 μM (IC_50_) | (Phainuphong et al., 2018) |
|  | MTT/Vero | 17.0 μM (IC_50_) | Ellipticine 4.0 μM (IC_50_) | (Saetang et al., 2021) |
|  | SRB/ACHN | 14.8 μM (IC_50_) | Adriamycin 0.15 μM (IC_50_) | (Anh et al., 2022) |
|  | SRB/NCI-H23 | 13.8 μM (IC_50_) | Adriamycin 0.12 μM (IC_50_) | (Anh et al., 2022) |
|  | SRB/PC-3 | 16.9 μM (IC_50_) | Adriamycin 0.15 μM (IC_50_) | (Anh et al., 2022) |
|  | SRB/NUGC-3 | 13.9 μM (IC_50_) | Adriamycin 0.15 μM (IC_50_) | (Anh et al., 2022) |
|  | SRB/MDA-MB-231 | 12.9 μM (IC_50_) | Adriamycin 0.16 μM (IC_50_) | (Anh et al., 2022) |
|  | SRB/HCT-15 | 14.3 μM (IC_50_) | Adriamycin 0.15 μM (IC_50_) | (Anh et al., 2022) |
| 7-Bromofolipastatin (**159**) | MTT/NS-1 | 12.5 μg/mL (MIC) | 5-Fluorouracil 0.1 μg/mL (MIC) | (Morshed et al., 2018) |
| Emeguisin A (**160**) | MTT/NS-1 | 12.5 μg/mL (MIC) | 5-Fluorouracil 0.1 μg/mL (MIC) | (Morshed et al., 2018) |
|  | MTT/KB | 14.41 μM (IC_50_) | Ellipticine 10.56 μM (IC_50_) | (Phainuphong et al., 2018) |
|  | MTT/MCF-7 | 30.90 μM (IC_50_) | Doxorubicin 16.83 μM (IC_50_) | (Phainuphong et al., 2018) |
|  | MTT/Vero | 15.65 μM (IC_50_) | Ellipticine 4.55 μM (IC_50_) | (Phainuphong et al., 2018) |
|  | MTT/Vero | 16.0 μM (IC_50_) | Ellipticine 4.0 μM (IC_50_) | (Saetang et al., 2021) |
|  | SRB/ACHN | 16.5 μM (IC_50_) | Adriamycin 0.15 μM (IC_50_) | (Anh et al., 2022) |
|  | SRB/NCI-H23 | 12.4 μM (IC_50_) | Adriamycin 0.12 μM (IC_50_) | (Anh et al., 2022) |
|  | SRB/PC-3 | 14.0 μM (IC_50_) | Adriamycin 0.15 μM (IC_50_) | (Anh et al., 2022) |
|  | SRB/NUGC-3 | 13.2 μM (IC_50_) | Adriamycin 0.15 μM (IC_50_) | (Anh et al., 2022) |
|  | SRB/MDA-MB-231 | 14.6 μM (IC_50_) | Adriamycin 0.16 μM (IC_50_) | (Anh et al., 2022) |
|  | SRB/HCT-15 | 10.5 μM (IC_50_) | Adriamycin 0.15 μM (IC_50_) | (Anh et al., 2022) |
| 4-Formyl-3,8-dihydroxy-9-methyl-1,6-bis(1-methyl-1-propenyl)-11*H*-dibenzo [b,e][1,4]dioxepin-11-one (**165**) | CCK-8/HeLa | 45.3 μM (IC_50_) | Cisplatin 11.7 μM (IC_50_) | (Zhao et al., 2020) |
|  | CCK-8/PC-3 | 22.2 μM (IC_50_) | Cisplatin 5.6 μM (IC_50_) | (Zhao et al., 2020) |
|  | CCK-8/A549 | 11.4 μM (IC_50_) | Cisplatin 11.8 μM (IC_50_) | (Zhao et al., 2020) |
|  | CCK-8/HepG-2 | 15.3 μM (IC_50_) | Cisplatin 9.3 μM (IC_50_) | (Zhao et al., 2020) |
|  | CCK-8/HL-60 | 22.3 μM (IC_50_) | Cisplatin 15.7 μM (IC_50_) | (Zhao et al., 2020) |
| 3,8-Dihydroxy-4-(hydroxymethyl)-9-methyl-1,6-bis(1-methyl-1-propenyl)- 11*H*-dibenzo[b,e][1,4]dioxepin-11-one (**166**) | CCK-8/HeLa | 36.1 μM (IC_50_) | Cisplatin 11.7 μM (IC_50_) | (Zhao et al., 2020) |
|  | CCK-8/A549 | 35.8 μM (IC_50_) | Cisplatin 11.8 μM (IC_50_) | (Zhao et al., 2020) |
| 3,8-Dihydroxy-4-(methoxymethyl)-9-methyl-1,6-bis(1-methyl-1-propenyl)-11*H*-dibenzo[b,e][1,4]dioxepin-11-one (**167**) | CCK-8/HeLa | 53.4 μM (IC_50_) | Cisplatin 11.7 μM (IC_50_) | (Zhao et al., 2020) |
|  | CCK-8/PC-3 | 58.1 μM (IC_50_) | Cisplatin 5.6 μM (IC_50_) | (Zhao et al., 2020) |
|  | CCK-8/A549 | 59.4 μM (IC_50_) | Cisplatin 11.8 μM (IC_50_) | (Zhao et al., 2020) |
|  | CCK-8/HepG-2 | 24.6 μM (IC_50_) | Cisplatin 9.3 μM (IC_50_) | (Zhao et al., 2020) |
|  | CCK-8/HL-60 | 57.6 μM (IC_50_) | Cisplatin 15.7 μM (IC_50_) | (Zhao et al., 2020) |
| Paucinervin Q (**169**) | MTT/HL-60 | 3.11 μM (IC_50_) | 5-Fluorouracil 2.39 μM (IC_50_) | (Jia et al., 2019) |
|  | MTT/PC-3 | 18.57 μM (IC_50_) | 5-Fluorouracil 30.59 μM (IC_50_) | (Jia et al., 2019) |
|  | MTT/CaCo-2 | 6.78 μM (IC_50_) | 5-Fluorouracil 38.77 μM (IC_50_) | (Jia et al., 2019) |

**Table S5**. Reported anti-inflammatory activity of naturally occurring depsidones.

| **Compound name** | **Assay/Cell Line** | **Biological Results** | | **Ref.** |
| --- | --- | --- | --- | --- |
|  |  | **Compound** | **Positive Control** |  |
| Boremexin A (**82**) | Spectrophotometric/No production in LPS-induced RAW264.7 | 30.3 µM (IC_50_) | PDTC 23.1 µM (IC_50_) | (Chen et al., 2020) |
| Boremexin B (**83**) | Spectrophotometric/No production in LPS-induced RAW264.7 | 34.4 µM (IC_50_) | PDTC 23.1 µM (IC_50_) | (Chen et al., 2020) |
| Boremexin C (**84**) | Spectrophotometric/No production in LPS-induced RAW264.7 | 19.9 µM (IC_50_) | PDTC 23.1 µM (IC_50_) | (Chen et al., 2020) |
| Curdepsidone F (**34**) | Spectrophotometric/No production in LPS-induced RAW264.7 | 26.6 µM (IC_50_) | PDTC 23.1 µM (IC_50_) | (Chen et al., 2020) |
| Corynesidone A (**7**) | Spectrophotometric/No production in LPS-induced RAW264.7 | 22.6 µM (IC_50_) | PDTC 23.1 µM (IC_50_) | (Chen et al., 2020) |
| Guanxidone A (**55**) | Spectrophotometric/No production in LPS-induced RAW264.7 | 8.22 µM (IC_50_) | Dexamethasone 5.62 μM (IC_50_) | (Hao et al., 2022) |

**Table 6S.** Reported AG inhibitory activity of naturally occurring depsidones.

| **Compound name** | **Biological Results*** | | **Ref.** |
| --- | --- | --- | --- |
|  | **Compound** | **Positive Control** |  |
| Botryorhodine C (**23**) | 11.2 μM (IC_50_) | Acarbose 703.8 μM (IC_50_) | (Zhang et al., 2018) |
| Botryorhodine D (**24**) | 10.3 μM (IC_50_) | Acarbose 703.8 μM (IC_50_) | (Zhang et al., 2018) |
| Botryorhodine G (**25**) | 54.1 μM (IC_50_) | Acarbose 703.8 μM (IC_50_) | (Zhang et al., 2018) |
| Parmosidone F (**53**) | 11.4 μM (IC_50_) | Acarbose 449.0 μM (IC_50_) | (Duong et al., 2020) |
| Parmosidone F1 (**54**) | 2.2 μM (IC_50_) | Acarbose 449.0 μM (IC_50_) | (Devi et al., 2020) |
| Salazinic acid (**65**) | 34.8 μM (IC_50_) | Acarbose 449.0 μM (IC_50_) | (Devi et al., 2020) |
| Parmosidone I (**77**) | 10.7 μM (IC_50_) | Acarbose 449.0 μM (IC_50_) | (Duong et al., 2020) |
| Parmosidone G1 (**78**) | 4.3 μM (IC_50_) | Acarbose 449.0 μM (IC_50_) | (Devi et al., 2020) |
| Parmosidone H (**79**) | 17.6 μM (IC_50_) | Acarbose 449.0 μM (IC_50_) | (Duong et al., 2020) |
| Parmosidone K (**81**) | 3.12 μM (IC_50_) | Acarbose 162.54 μM (IC_50_) | (Nguyen et al., 2022a) |
| Botryorhodine H (**88**) | 8.1 μM (IC_50_) | Acarbose 703.8 μM (IC_50_) | (Zhang, L. et al., 2018) |

* Spectrophotometric assay

**References**

**Addo EM, Ren Y, Anaya-Eugenio GD, Ninh TN, Rakotondraibe HL, de Blanco, Esperanza J Carcache, Soejarto DD, Kinghorn AD. 2021.** Spermidine alkaloid and glycosidic constituents of vietnamese *Homalium cochinchinensis*. Phytochemistry Letters **43:**154-62.

**An F, Liu W, Wei X, Pan Z, Lu Y. 2018.** Curdepsidone A, a depsidone from the marine-derived endophytic fungus *curvularia* sp. IFB-Z10. Natural Product Communications **13(7)**:1934578X1801300720.

**Anh CV, Kwon J, Kang JS, Lee H, Heo C, Shin HJ. 2022.** Antibacterial and cytotoxic phenolic polyketides from two marine-derived fungal strains of *Aspergillus unguis*. Pharmaceuticals (Basel) **15(1):**74.

**Bai SZ, Yang LG, Bai L. 2021.** Two new depsidones from parmelia saxatilis. Chemistry of Natural Compounds **57(1):**63-65.

**Bay MV, Nam PC, Quang DT, Mechler A, Hien NK, Hoa NT, Vo QV. 2020.** Theoretical study on the antioxidant activity of natural depsidones. ACS Omega **5(14):**7895-902.

**Bui V, Duong T, Chavasiri W, Nguyen K, Huynh B.** 2**022.** A new depsidone from the lichen usnea ceratina arch. Natural Products Research **36(9):**2263-2269.

**Burt SR, Harper JK, Cool LG. 2022.** A new depsidone from the neotricone-rich chemotype of the lichenised fungus *Usnea fulvoreagens*. Natural Products Research:1-7.

**Carpentier C, Barbeau X, Azelmat J, Vaillancourt K, Grenier D, Lagüe P, Voyer N. 2018.** Lobaric acid and pseudodepsidones inhibit NF-κB signaling pathway by activation of PPAR-γ. Bioorganic & medicinal chemistry **26(22):**5845-51.

**Chen Y, Sun L, Yang H, Li Z, Liu J, Ai H, Wang G, Feng T. 2020.** Depsidones and diaryl ethers from potato endophytic fungus *Boeremia exigua*. Fitoterapia **141**:104483.

**Devi AP, Duong T, Ferron S, Beniddir MA, Dinh M, Nguyen V, Mac D, Boustie J, Chavasiri W, Le Pogam P. 2020.** Salazinic acid-derived depsidones and diphenylethers with α-glucosidase inhibitory activity from the lichen *Parmotrema dilatatum*. Planta Medica **86(16):**1216-24.

**Ding Y, An F, Zhu X, Yu H, Hao L, Lu Y. 2019.** Curdepsidones B–G, six depsidones with anti-inflammatory activities from the marine-derived fungus *Curvularia* sp. IFB-Z10. Marine Drugs **17(5):**266.

**Dongmo Zeukang R, Siwe-Noundou X, Tagatsing Fotsing M, Tabopda Kuiate T, Mbafor JT, Krause RW, Choudhary MI, Atchadé AdT. 2019.** Cordidepsine is a potential new anti-HIV depsidone from *Cordia millenii*, baker. Molecules **24(17):**3202.

**Duong HT. 2019.** Phenolic compounds from *Pamotrema dilatatum* growing in Lam Dong province. Science and Technology Development Journal **22(1):**114-119.

**Duong T, Chavasiri W, Boustie J, Nguyen K. 2015.** New meta-depsidones and diphenyl ethers from the lichen *Parmotrema tsavoense* (Krog & Swinscow) Krog & Swinscow, Parmeliaceae. Tetrahedron **71(52):**9684-9691.

**Duong T, Le Pogam P, Tran T, Mac D, Dinh M, Sichaem J. 2020.** Α-glucosidase inhibitory depsidones from the lichen parmotrema tsavoense. Planta Medica **86(11):**776-781.

**Guo Z, Zhu W, Zhao L, Chen Y, Li S, Cheng P, Ge H, Tan R, Jiao R. 2022.** New antibacterial depsidones from an ant-derived fungus *Spiromastix* sp. MY-1. Chinese Journal of Natural Medicines **20(8):**627-632.

**Hao L, Zhou D, Qin X, Zhang W, Yang R, Li J, Huang X. 2022.** A new depsidone derivative from mangrove endophytic fungus *Aspergillus* sp. GXNU-A9. Natural Product Research **36(7):**1878-1882.

**Hartati S, Megawati M, Antika LD. 2022.** Parvidepsidone, a novel depsidone from the barks of *Garcinia parvifolia* Miq. Natural Product Sciences **28(1):**13-17.

**He R, Wang Y, Yang B, Liu Z, Li D, Zou B, Huang Y. 2022.** Structural characterization and assessment of anti-inflammatory activities of polyphenols and depsidone derivatives from *Melastoma malabathricum* subsp. *normale*. Molecules **27(5):**1521.

**Hyung Koo M, Shin M, Ju Kim M, Lee S, Eun So J, Hee Kim J, Hyuck Lee J, Suh S, Joung Youn U. 2022.** Bioactive secondary metabolites isolated from the Antarctic lichen *Himantormia lugubris*. Chemistry & Biodiversity **19(10):**e202200374.

**Ismed F, Putra HE, Arifa N, Putra DP. 2021.** Phytochemical profiling and antibacterial activities of extracts from five species of Sumatran lichen genus *Stereocaulon*. Jordan Journal of Pharmaceutical Sciences 14(2):189-202.

**Jia C, Xue J, Li X, Li D, Li Z, Hua H. 2019.** New depsidone and dichromone from the stems of *Garcinia paucinervis* with antiproliferative activity. Journal of Natural Medicines **73(1):**278-282.

**Jin Y, Ma Y, Xie W, Hou L, Xu H, Zhang K, Zhang L, Du Y. 2018.** UHPLC-Q-TOF-MS/MS-oriented characteristic components dataset and multivariate statistical techniques for the holistic quality control of usnea. RSC Advances **8(28):**15487-15500.

**Lage TC, Maciel TMS, Mota YC, Sisto F, Sabino JR, Santos JC, Figueiredo IM, Masia C, de Fátima Â, Fernandes SA. 2018.** In vitro inhibition of helicobacter pylori and interaction studies of lichen natural products with jack bean urease. New Journal of Chemistry **42(7):**5356-66.

**Lannang AM, Sema DK, Tatsimo SJ, Tankeu VT, Tegha HF, Wansi JD, Shiono Y, Sewald N. 2018.** A new depsidone derivative from the leaves of *Garcinia polyantha*. Natural Product Research **32(9):**1033-1038.

**Leal A, Rojas JL, Valencia-Islas NA, Castellanos L. 2018.** New β-orcinol depsides from *Hypotrachyna caraccensis*, a lichen from the páramo ecosystem and their free radical scavenging activity. Natural Product Research **32(12):**1375-1382.

**Liang Y, Zheng Y, Shen Y, Li Q, Lu Y, Ye S, Li X, Li D, Chen C, Zhu H, Zhang, Y. 2022.** Two new depsidones from the fungus *Lasiodiplodia pseudotheobromae*. Tetrahedron Letters **105:**154046.

**Mathioudaki A, Berzesta A, Kypriotakis Z, Skaltsa H, Heilmann J. 2018.** Phenolic metabolites from *Hypericum kelleri* Bald., an endemic species of Crete (Greece). Phytochemistry **146:**1-7.

**Morshed MT, Vuong D, Crombie A, Lacey AE, Karuso P, Lacey E, Piggott AM. 2018.** Expanding antibiotic chemical space around the nidulin pharmacophore. Organic & Biomolecular Chemistry **16(16):**3038-3051.

**Nakashima K, Tomida J, Kamiya T, Hirai T, Morita Y, Hara H, Kawamura Y, Adachi T, Inoue M. 2018.** Diaporthols A and B: Bioactive diphenyl ether derivatives from an endophytic fungus *Diaporthe* sp. Tetrahedron Letters **59(13):**1212-1215.

**Nguyen T, Nguyen T, Nguyen T, Nguyen H, Nguyen N, Mai D, Huynh B, Tran C, Duong T. 2022a.** Parmosidone K, a new meta-depsidone from the lichen *Parmotrema tsavoense*. Natural Products Research **36**(8):2037-42.

**Nguyen V, Genta-Jouve G, Duong T, Beniddir MA, Gallard J, Ferron S, Boustie J, Mouray E, Grellier P, Chavasiri W. 2020.** Eumitrins CE: Structurally diverse xanthone dimers from the Vietnamese lichen *Usnea baileyi*. Fitoterapia **141:**104449.

**Niu S, Liu D, Shao Z, Huang J, Fan A, Lin W. 2021.** Chlorinated metabolites with antibacterial activities from a deep-sea-derived *Spiromastix* fungus. RSC Advances **11(47):**29661-29667.

**Nugraha AS, Dayli IR, Sukrisno Putri, Chintya Permata Zahky, Firli LN, Widhi Pratama AN, Triatmoko B, Untari LF, Wongso H, Keller PA, Wangchuk P. 2022.** Isolation of antibacterial depside constituents from Indonesian folious lichen, *Candelaria fibrosa*. Journal of Biologically Active Products from Nature **12(1):**24-32.

**Olivon F, Nothias L, Dumontet V, Retailleau P, Berger S, Ferry G, Cohen W, Pfeiffer B, Boutin JA, Scalbert E. 2018.** Natural inhibitors of the RhoA–p115 complex from the bark of Meiogyne baillonii. Journal of Natural Products **81(7):**1610-1618.

**Ouyang J, Mao Z, Guo H, Xie Y, Cui Z, Sun J, Wu H, Wen X, Wang J, Shan T. 2018.** Mollicellins O–R, four new depsidones isolated from the endophytic fungus *Chaetomium* sp. eef-10. Molecules **23(12):**3218.

**Pavan Kumar P, Siva B, Anand A, Tiwari AK, Vekata Rao C, Boustie J, Suresh Babu K. 2020.** Isolation, semi-synthesis, free-radicals scavenging, and advanced glycation end products formation inhibitory constituents from *Parmotrema tinctorum*. Journal of Asian Natural Products Research **22(10):**976-988.

**Phainuphong P, Rukachaisirikul V, Phongpaichit S, Sakayaroj J, Kanjanasirirat P, Borwornpinyo S, Akrimajirachoote N, Yimnual C, Muanprasat C. 2018.** Depsides and depsidones from the soil-derived fungus *Aspergillus unguis* PSU-RSPG204. Tetrahedron **74(39):**5691-5699.

**Promgool T, Kanokmedhakul K, Leewijit T, Song J, Soytong K, Yahuafai J, Kudera T, Kokoska L, Kanokmedhakul S. 2022.** Cytotoxic and antibacterial depsidones from the endophytic fungus *Chaetomium brasiliense* isolated from Thai rice. Natural Product Research **36(18):**4605-4613.

**Rukachaisirikul V, Chinpha S, Saetang P, Phongpaichit S, Jungsuttiwong S, Hadsadee S, Sakayaroj J, Preedanon S, Temkitthawon P, Ingkaninan K. 2019.** Depsidones and a dihydroxanthenone from the endophytic fungi *Simplicillium lanosoniveum* (JFH beyma) zare & W. gams PSU-H168 and PSU-H261. Fitoterapia **138:**104286.

**Sadorn K, Saepua S, Bunbamrung N, Boonyuen N, Komwijit S, Rachtawee P, Pittayakhajonwut P. 2022.** Diphenyl ethers and depsidones from the endophytic fungus *Aspergillus unguis* BCC54176. Tetrahedron **105:**132612.

**Saetang P, Rukachaisirikul V, Phongpaichit S, Preedanon S, Sakayaroj J, Hadsadee S, Jungsuttiwong S. 2021.** Antibacterial and antifungal polyketides from the fungus *Aspergillus unguis* PSU-MF16. Journal of Natural Products **84(5):**1498-1506.

**Sanjaya A, Avidlyandi A, Adfa M, Ninomiya M, Koketsu M. 2020.** A new depsidone from *Teloschistes flavicans* and the antileukemic activity. Journal of Oleo Science:ess20209.

**Sedrpoushan A, Haghi H, Sohrabi M. 2022.** A new secondary metabolite profiling of the lichen *Diploschistes diacapsis* using liquid chromatography electrospray ionization tandem mass spectrometry. Inorganic Chemistry Communications **145:**110006.

**Sepúlveda B, Cornejo A, Bárcenas-Pérez D, Cheel J, Areche C. 2022.** Two new fumarprotocetraric acid lactones identified and characterized by UHPLC-PDA/ESI/ORBITRAP/MS/MS from the Antarctic lichen *Cladonia metacorallifera*. Separations **9(2):**41.

**Shiono Y, Koseki T, Koyama H. 2018.** A bioactive depsidone from *Lachnum virgineum* (Hyaloscyphaceae). Natural Product Sciences **24(2):**79-81.

**Shukla I, Azmi L, Gupta SS, Upreti DK, Rao CV. 2019.** Amelioration of anti-hepatotoxic effect by *Lichen rangiferinus* against alcohol induced liver damage in rats. Journal of Ayurveda and Integrative Medicine **10(3):**171-177.

**Singh G, Armaleo D, Dal Grande F, Schmitt I. 2021.** Depside and depsidone synthesis in lichenized fungi comes into focus through a genome-wide comparison of the olivetoric acid and physodic acid chemotypes of *Pseudevernia furfuracea*. Biomolecules **11(10):**1445.

**Studzińska-Sroka E, Majchrzak-Celińska A, Zalewski P, Szwajgier D, Baranowska-Wójcik E, Żarowski M, Plech T, Cielecka-Piontek J. 2021.** Permeability of hypogymnia physodes extract Component—Physodic acid through the Blood–Brain barrier as an important argument for its anticancer and neuroprotective activity within the central nervous system. Cancers **13(7):**1717.

**Tatipamula VB, Annam SS. 2022.** Antimycobacterial activity of acetone extract and isolated metabolites from folklore medicinal lichen *Usnea laevis* Nyl. against drug-sensitive and multidrug-resistant tuberculosis strains. Journal of Ethnopharmacology **282**:114641.

**Umeokoli BO, Ebrahim W, El-Neketi M, Müller WE, Kalscheuer R, Lin W, Liu Z, Proksch P. 2019.** A new depsidone derivative from mangrove sediment derived fungus *Lasiodiplodia theobromae*. Natural Product Research **33(15):**2215-2222.

**Van Nguyen K, Duong T, Nguyen KPP, Sangvichien E, Wonganan P, Chavasiri W. 2018.** Chemical constituents of the lichen usnea baileyi (stirt.) zahlbr. Tetrahedron Letters **59(14):**1348-1351.

**Yang L, Wang Z, Wang Y, Li R, Wang F, Wang K. 2019.** Phenolic constituents with neuroprotective activities from *Hypericum wightianum*. Phytochemistry **165:**112049.

**Yang W, Bao H, Liu Y, Nie Y, Yang J, Hong P, Zhang Y. 2018.** Depsidone derivatives and a cyclopeptide produced by marine fungus *Aspergillus unguis* under chemical induction and by its plasma induced mutant. Molecules **23(9):**2245.

**Zhang L, Niaz SI, Wang Z, Zhu Y, Lin Y, Li J, Liu L. 2018.** α-Glucosidase inhibitory and cytotoxic botryorhodines from mangrove endophytic fungus *Trichoderma* sp. 307. Natural Product Research **32(24):**2887-2892.

**Zhang Y, Li Z, Huang B, Liu K, Peng S, Liu X, Gao C, Liu Y, Tan Y, Luo X. 2022.** Anti-osteoclastogenic and antibacterial effects of chlorinated polyketides from the Beibu Gulf coral-derived fungus *Aspergillus unguis* GXIMD 02505. Mar Drugs **20(3):**178.

**Zhao C, Liu G, Liu X, Zhang L, Li L, Liu L. 2020.** Pycnidiophorones A–D, four new cytochalasans from the wetland derived fungus *Pycnidiophora dispersa*. RSC Advances **10(66):**40384-40390.

**Zhao P, Yang M, Zhu G, Zhao B, Wang H, Liu H, Wang X, Qi J, Yin X, Yu L, Meng Y, Li Z, Zhang L, Xia X. 2021.** Mollicellins S-U, three new depsidones from *Chaetomium brasiliense* SD-596 with anti-MRSA activities. The Journal of Antibiotics (Tokyo) **74(5):**317-323.
